# New Triazole-Based Potent Inhibitors of Human Factor XIIa as Anticoagulants

**DOI:** 10.1021/acsomega.3c09335

**Published:** 2024-02-22

**Authors:** Ma’Lik D. Woodland, Anthony Thompson, Amanda Lipford, Navneet Goyal, John C. Schexnaildre, Madhusoodanan Mottamal, Daniel K. Afosah, Rami A. Al-Horani

**Affiliations:** † Division of Basic Pharmaceutical Sciences, College of Pharmacy, Xavier University of Louisiana, New Orleans, Louisiana 70125, United States; ‡ Department of Chemistry, Xavier University of Louisiana, New Orleans, Louisiana 70125, United States; § Department of Medicinal Chemistry, School of Pharmacy, Virginia Commonwealth University, Richmond, Virginia 23219, United States

## Abstract

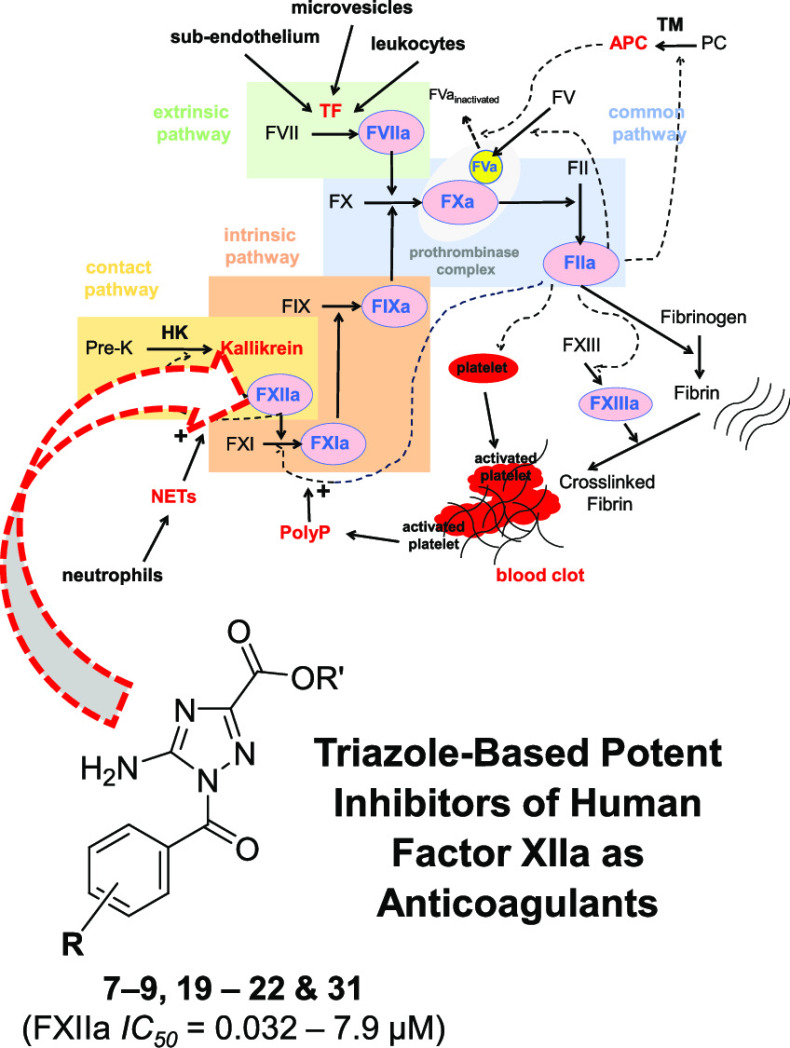

Factor XIIa (FXIIa) functions as a plasma serine protease within the contact activation pathway. Various animal models have indicated a substantial role for FXIIa in thromboembolic diseases. Interestingly, individuals and animals with FXII deficiency seem to maintain normal hemostasis. Consequently, inhibiting FXIIa could potentially offer a viable therapeutic approach for achieving effective and safer anticoagulation without the bleeding risks associated with the existing anticoagulants. Despite the potential, only a limited number of small molecule inhibitors targeting human FXIIa have been documented. Thus, we combined a small library of 32 triazole and triazole-like molecules to be evaluated for FXIIa inhibition by using a chromogenic substrate hydrolysis assay under physiological conditions. Initial screening at 200 μM involved 18 small molecules, revealing that 4 molecules inhibited FXIIa more than 20%. In addition to being the most potent inhibitor identified in the first round, inhibitor **8** also exhibited a substantial margin of selectivity against related serine proteases, including factors XIa, Xa, and IXa. However, the molecule also inhibited thrombin with a similar potency. It also prolonged the clotting time of human plasma, as was determined in the activated partial thromboplastin time and prothrombin time assays. Subsequent structure–activity relationship studies led to the identification of several inhibitors with submicromolar activity, among which inhibitor **22** appears to demonstrate significant selectivity not only over factors IXa, Xa, and XIa, but also over thrombin. In summary, this study introduces novel triazole-based small molecules, specifically compounds **8** and **22**, identified as potent and selective inhibitors of human FXIIa. The aim is to advance these inhibitors for further development as anticoagulants to provide a more effective and safer approach to preventing and/or treating thromboembolic diseases.

## Introduction

**Figure 1:**
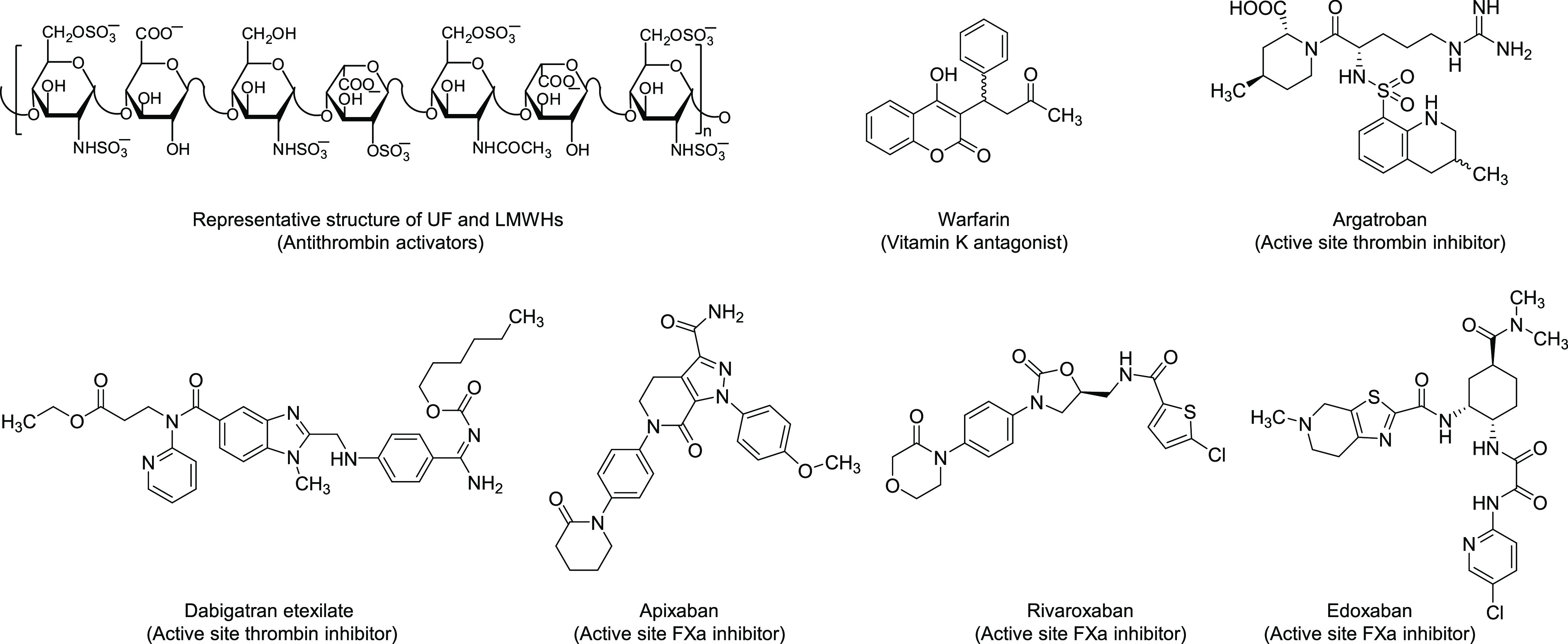
Chemical structures of current anticoagulants.

Thrombosis continues to be a significant global cause of mortality with conditions arising from both arterial and venous sources. Major arterial thrombotic events include ischemic heart disease and stroke, while venous thromboembolism (VTE) encompasses deep-vein thrombosis and pulmonary embolism. In the United States, cardiovascular diseases, particularly heart disease and stroke, are the primary contributors to mortality, with heart disease ranking as the leading cause of death.^1^ VTE is the third leading vascular diagnosis of cardiovascular-related deaths.^2^ Anticoagulant treatments are commonly employed for managing and preventing VTE and stroke in nonvalvular atrial fibrillation (AF). The historical trajectory of anticoagulants (Figure 1) has been prominently influenced by nonspecific agents like vitamin K antagonists (VKA, such as warfarin) and heparins [unfractionated heparin (UFH) and low molecular weight heparin (LMWH)], at least up until the past two decades. However, these conventional anticoagulants are linked to significant drawbacks.^3−5^ UFH suffers from significant intra- and interpatient response variation, necessitating frequent laboratory monitoring. Heparin-induced thrombocytopenia is a lethal complication of heparin therapy and may be associated with thrombosis. Other limitations encompass osteoporosis in individuals subjected to high-dose therapy over an extended duration and the heightened risk of contamination with other glycosaminoglycans, potentially leading to fatal hypersensitivity reactions. The introduction of LMWHs has mitigated many of these drawbacks.^3^ Warfarin, on the other hand, grapples with a narrow therapeutic index and numerous interactions with other drugs and dietary components.^4^

Subsequently, traditional anticoagulants have been supplanted by newer medications targeting specific coagulation factors. Examples include argatroban and bivalirudin (parenteral thrombin inhibitors), fondaparinux (a parenteral antithrombin-binding pentasaccharide, FPX), and direct oral anticoagulants (DOACs) (Figure 1). Since their initial approval in 2010, DOACs such as dabigatran (thrombin inhibitor - factor IIa, FIIa), apixaban, edoxaban, and rivaroxaban (inhibitors of factor Xa, FXa) have gained widespread usage for treating and preventing VTE and in nonvalvular AF. Due to their user-friendly nature, favorable pharmacological profiles, and not requiring constant monitoring, DOACs have largely replaced warfarin for many indications.^6^ Nevertheless, even though they exhibit a superior safety profile compared to heparins and warfarin, significant challenges arise from the restricted availability of standardized assays for measuring these drugs in biological fluids, their elevated cost, and potential contraindications in patients with severe renal dysfunction.^7,8^ Many DOACs are also P-glycoprotein substrates that carry significant drug–drug interaction issues. Nearly all direct anticoagulants require hepatic metabolism for elimination, which affects their use in patients with hepatic dysfunction.^9^

**Figure 2:**
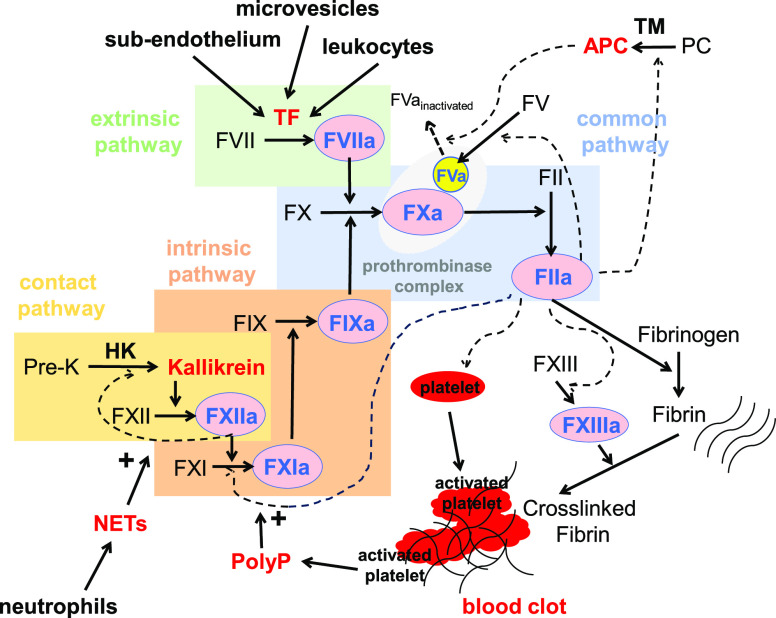
Coagulation processes. The extrinsic pathway is activated when tissue factor (TF) is exposed to subendothelium, circulating microvesicles, or leukocytes. TF activates factor VII (FVII) to FVIIa. The TF-FVIIa complex activates factor X (FX) to FXa, which forms the prothrombinase complex with cofactor Va (FVa) on negatively charged membrane surfaces. The prothrombinase complex converts prothrombin (FII) to thrombin (FIIa). The intrinsic pathway is initiated by activation of the contact pathway, whereby factor XII (FXII) and prekallikrein (Pre-K) engage in reciprocal activation by their activated forms, FXIIa and kallikrein, respectively. FXIIa subsequently activates factor XI (FXI) to FXIa, which subsequently activates factor IX (FIX) and then FX. Upon stimulation, platelets and neutrophils release procoagulant molecules, such as polyphosphate (PolyP) and neutrophil extracellular traps (NETs), respectively. These polyanions play a role in coagulation by enhancing the contact system and promoting thrombin-mediated activation of FXI. Ultimately, thrombin cleaves fibrinogen to produce fibrin, which undergoes cross-linking to ensnare activated platelets and other cells. High molecular weight kininogen (HK) is important for the activation of Pre-K and FXII. The clotting process is modulated by the activation of protein C (PC) through thrombin in the presence of thrombomodulin (TM). Activated PC (APC) suppresses coagulation by transforming FVa to its inactive state.

Importantly, all the conventional as well as the newer anticoagulants are associated with a significant risk of internal bleeding.^10−15^ Certainly, the risk of major bleeding during DOAC therapy is approximately 2% per year, with a case fatality rate of major bleeding standing at 8%.^16^ Consequently, there has been a concerted effort in developing and deploying specific antidotes for DOACs, especially following the FDA approval of two antidotes: idarucizumab for dabigatran and andexanet alfa for anti-FXa DOACs. The latter, owing to its steep cost and debatable effectiveness and safety, is not widely adopted. It is crucial to note that due to the heightened risk of bleeding, there are specific clinical scenarios where the use of DOACs is not advisable. These include frail patients (such as the elderly, those with low body weight, and individuals with renal impairment), patients with end-stage renal disease (ESRD) undergoing hemodialysis, and those with genitourinary and gastrointestinal cancers. Furthermore, DOACs may not be effective in certain clinical conditions, such as cases involving mechanical heart valves or antiphospholipid syndrome.^16^ Hence, a critical necessity exists for the advancement of new anticoagulants. Despite their structural diversity, all currently utilized anticoagulants either directly or indirectly target thrombin and/or FXa, which are two serine proteases belonging to the common pathway of coagulation (Figure 2).^3−5^ While this characteristic renders these molecules effective anticoagulants, it also serves as the primary cause of internal bleeding. The present study focuses on the development of novel anticoagulants that are both effective and safer by directly inhibiting FXIIa within the contact system of coagulation (Figure 2).

Human FXIIa is a serine protease belonging to the contact system, which is composed of factor XII (FXII), prekallikrein, and high molecular weight kininogen. The contact system requires an autoactivation step for initiation. FXII undergoes autoactivation in the presence of polyanions. FXIIa then activates prekallikrein bound to high molecular weight kininogen, generating kallikrein. Kallikrein engages in reciprocal activation with FXII, completing a cyclic amplification loop. Subsequently, FXIIa activates factor XI (FXI), initiating the intrinsic coagulation pathway.^17^ Notably, individuals with an inherited deficiency of FXII do not experience bleeding, even in the context of extensive surgery.^18,19^ Additionally, they exhibit a prolonged activated partial thromboplastin clotting time (APTT) with no impact on prothrombin time (PT).^20^ Due to the rarity of FXII deficiency in humans, the interest in targeting FXIIa for anticoagulation primarily stems from findings in FXII knockout mice. These mice exhibited protection against chemically and mechanically induced arterial and venous thromboses, coupled with normal bleeding times.^20−22^ Similar to humans, these mice displayed significantly prolonged activated partial thromboplastin clotting times without a propensity for bleeding.^20−22^ Notably, FXII null mice consistently demonstrated protection from thromboembolic diseases, including ischemic stroke^21^ and pulmonary embolism.^22^ These observations prompted the development of antibodies targeting FXII/FXIIa,^23,24^ recombinant proteins,^25−28^ and antisense oligonucleotides.^29,30^ These interventions showcased effective anticoagulant activity across various arterial and venous thrombosis models in mice, rabbits, and nonhuman primates, with no associated bleeding complications.^24−31^ Moreover, the pharmacological inhibition of FXIIa mitigated the severity of thrombo-inflammation-driven cardiovascular diseases in three distinct mouse models. In particular, a specific monoclonal antibody that inhibits FXIIa reduces the severity of abdominal aortic aneurysms, hinders the progression of atherosclerosis, and stabilizes vulnerable plaques.^32^ Overall, inhibition of FXIIa is a safe and efficient way of thrombosis prevention without bleeding.

**Figure 3:**
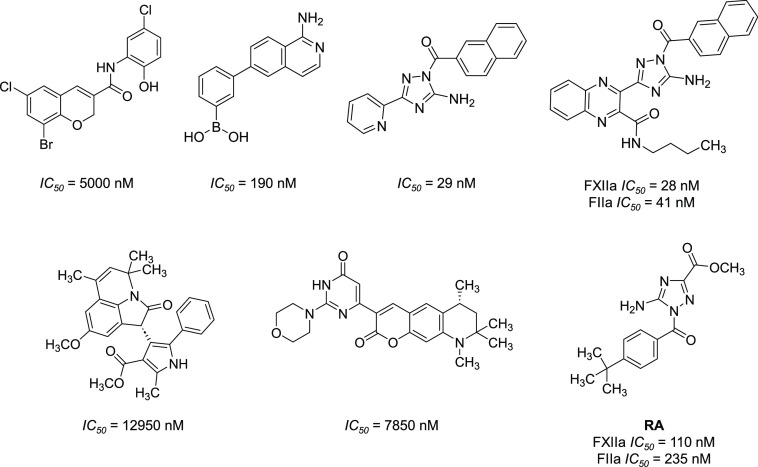
Chemical structures of previously reported FXIIa inhibitors.

Very few potent and selective small molecule inhibitors of FXIIa are in development (Figure 3).^33−38^ We previously reported inhibitor “**RA**” as a potent and selective inhibitor of FXIIa.^39^ In this study, we combined a small library of 32 triazole and triazole-like analogs of inhibitor **RA** to be evaluated for FXIIa inhibition using a chromogenic substrate hydrolysis assay under physiological conditions. Initial enzyme inhibition screening, subsequent structure–activity relationship studies, human plasma clotting assays, and molecular modeling studies revealed molecules **8** and **22** as potent and selective inhibitors of human FXIIa that can be further considered for the development of effective and safer anticoagulants.

## Results and Discussion

### Constructing the Library of Triazole and Triazole-Like Molecules and the Initial Screening for Human FXIIa Inhibitors

**Figure 4:**
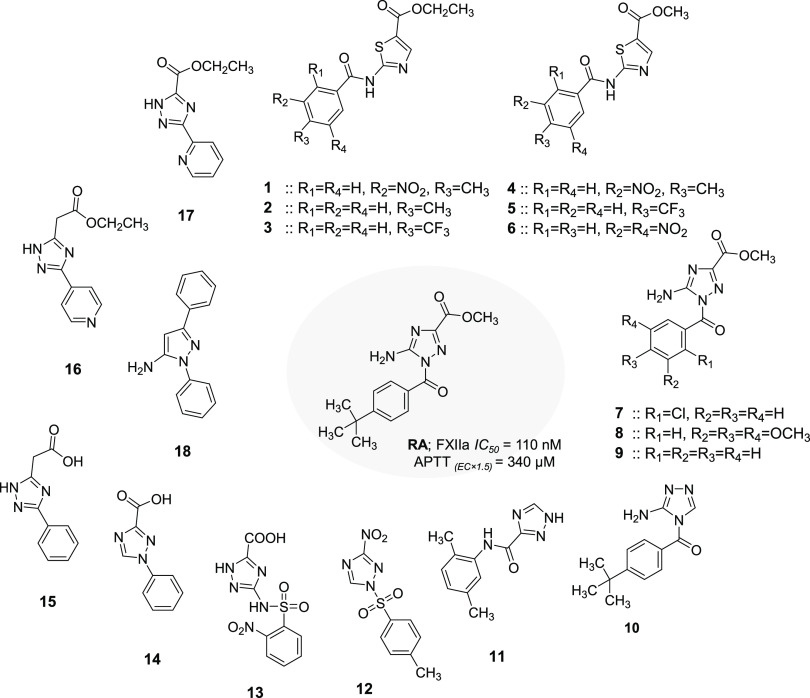
Chemical structures of 18 triazole and triazole-like molecules are evaluated for FXIIa inhibition by using a chromogenic substrate hydrolysis assay under physiological conditions. At the center is a previously reported FXIIa inhibitor, **RA**. Some compounds in the library were synthesized (**1**–**10**), and others were purchased from chemical vendors.

**Scheme 1:**
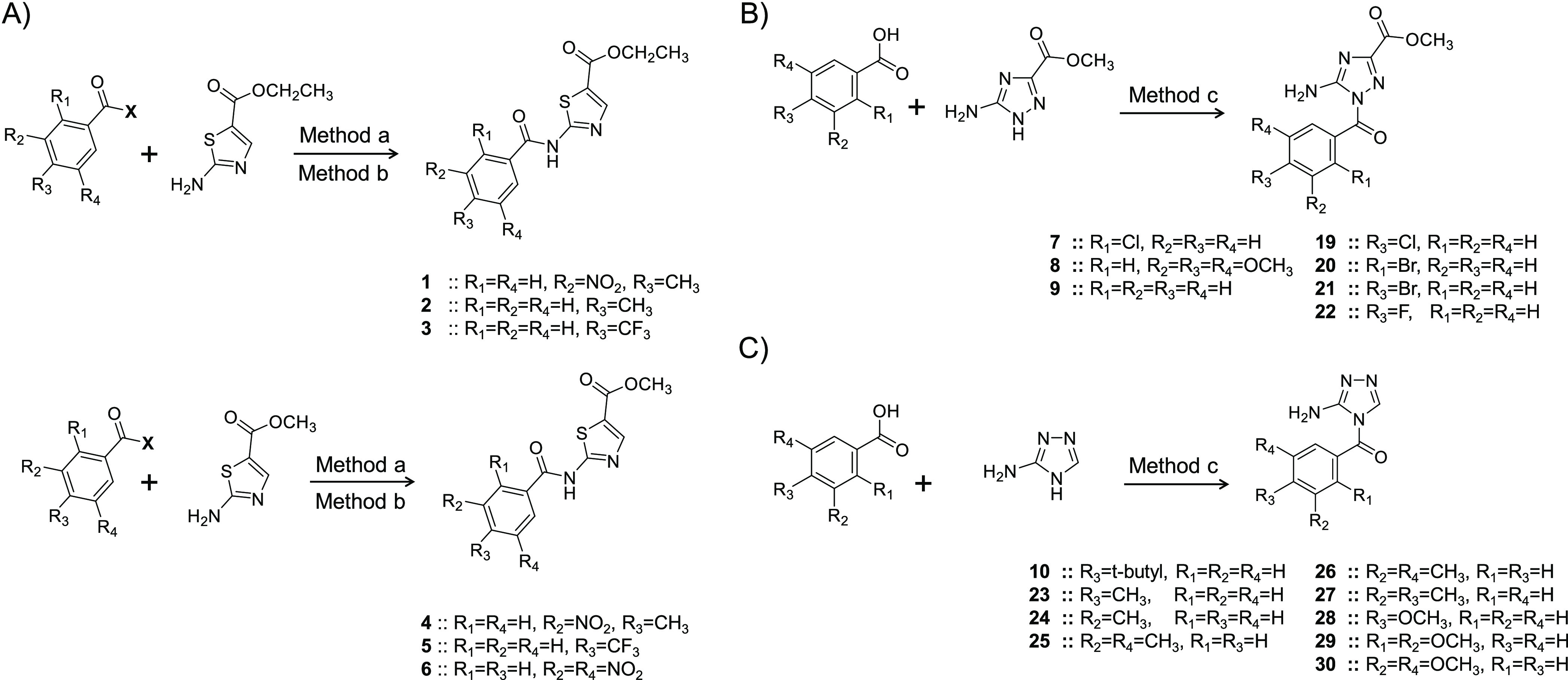
Synthesis of Molecules **1**–**10** and **19**–**30** (A) Synthesis of ethyl (or methyl) 2-aminothiazole-5-carboxylate-based molecules (**1**–**6**) was carried out overnight in dichloromethane under basic conditions (triethylamine/n-methyl morpholine): Method a: Reaction is between the thiazole and benzoic acid derivatives in the presence of EDCI and HOBt. Method b: Reaction is between the thiazole and benzoyl chlorides. (B) Synthesis of methyl 5-amino-1H-1,2,4-triazole-3-carboxylate-based molecules (**7** and **8**). (C) Synthesis of 1H-1,2,4-triazol-5-amine molecules (**9** and **10**). Method c: Reaction was carried out overnight in dichloromethane under basic conditions (DMAP). It involves a reaction between the triazole and benzoic acid derivatives in the presence of EDCI.

Given our previous results in identifying the potent and selective FXIIa inhibitor **RA,**(39) our current efforts were unfolded at two levels (Figure 4). The first level was (i) to synthesize analogs in which the amino-triazole moiety was replaced with an amino-thiazole moiety (**1**–**6**); (ii) to synthesize analogs with amino-triazole moiety, yet with different substitution patterns from inhibitor **RA** (**7**–**10**); and last (iii) to obtain commercially available analogs of inhibitor **RA** with various modifications (**11**–**18**). The second level was to chemically synthesize new analogs of the best inhibitor identified in the first level above, which led to the synthesis of inhibitors **19**–**32** (Scheme 1B,C).

In the first level, we decided to use the amino-thiazole moiety to engineer a hydrophobic characteristic that can potentially enhance the selectivity of target inhibition. Following chemical methods “a” and “b” (Scheme 1A), we were able to chemically synthesize six molecules (**1**–**6**). The molecules had either ethyl-ester substituent (**1**–**3**) or methyl-ester substituent (**4**–**6**). To ensure structural diversity, the molecules’ benzoyl moiety was variably monosubstituted and disubstituted with electron-withdrawing groups (such as CF_3_ and NO_2_) and/or electron-releasing groups (such as CH_3_). Substituents were introduced at either the *meta*-position or *para*-position or both. The preparation of these molecules entailed one step of EDCI-mediated amidation or acylation using benzoyl chlorides under basic conditions (Scheme 1A). The amino-triazole analogs of inhibitor RA synthesized (method “c” in Scheme 1B,C) at this phase had different substitution patterns. Inhibitor **7** is monosubstituted at the ortho-position with an electron-withdrawing group, i.e., Cl. Inhibitor **8** is trisubstituted at meta- and para-positions with electron-releasing groups, i.e., OCH_3._ Inhibitor **9** is an unsubstituted analog. Inhibitor **10** lacks the methyl-carboxylate ester of inhibitor RA. Important to mention here is that the choice of all analogs was largely determined based on the commercial availability of the benzoic acid or benzoyl chloride precursors. Lastly, a majority of the commercially available analogs (**11**–**18**) of inhibitor **RA**, which were acquired for this study, had a triazole central domain, yet its methyl-ester was either eliminated (**11**) or replaced with nitro group (**12**), carboxylic acid group (**13** and **14**), acetic acid (**15**), ethyl-acetate group (**16**), ethyl-ester group (**17**), or a phenyl ring (**18**). The amide linkage in inhibitor **RA** was also replaced by a sulfonamide linkage in molecules **12** and **13**. The phenyl group was also replaced with pyridine in molecules **16** and **17**. Those commercially available molecules with the phenyl ring were either monosubstituted (**11**–**13**) or unsubstituted (**14**, **15**, and **18**).

In the second level, we synthesized, following method “c” in Scheme 1B,C, 14 inhibitors (**19**–**32**) with the amino-triazole as the central moiety. Molecules **19**–**22, 31**, and **32** do have ester functionality, whereas molecules **10** and **23**–**30** do not have that functionality. Inhibitors **19**, **21**, and **22** are the para-position halogenated (Cl, Br, and F, respectively) congeners of lead inhibitor **RA**. Inhibitor **20** is an analog of inhibitor **21**, for which the bromine substituent is replaced from the *para*-position to the *ortho*-position. Likewise, inhibitor **19** is an analog of inhibitor **7**, where the chlorine substituent is at the para-position in the former molecule and at the *ortho*-position in the latter. Inhibitor **31** possesses ethyl-ester, whereas inhibitor **9** is a methyl ester-containing analog. Inhibitor **32** is the acetylated analog of inhibitor **9**. Inhibitors **23**–**30** are the methylated and methoxylated, mono- and disubstituted analogs of inhibitor **10**. Overall, we have constructed a diverse library of 32 small molecule potential FXIIa inhibitors to establish a meaningful structure–activity relationship that can further drive our efforts to design clinically relevant potent and selective inhibitors of human FXIIa.

### New Analogs and Structure–Activity Relationship Studies

**Figure 5:**
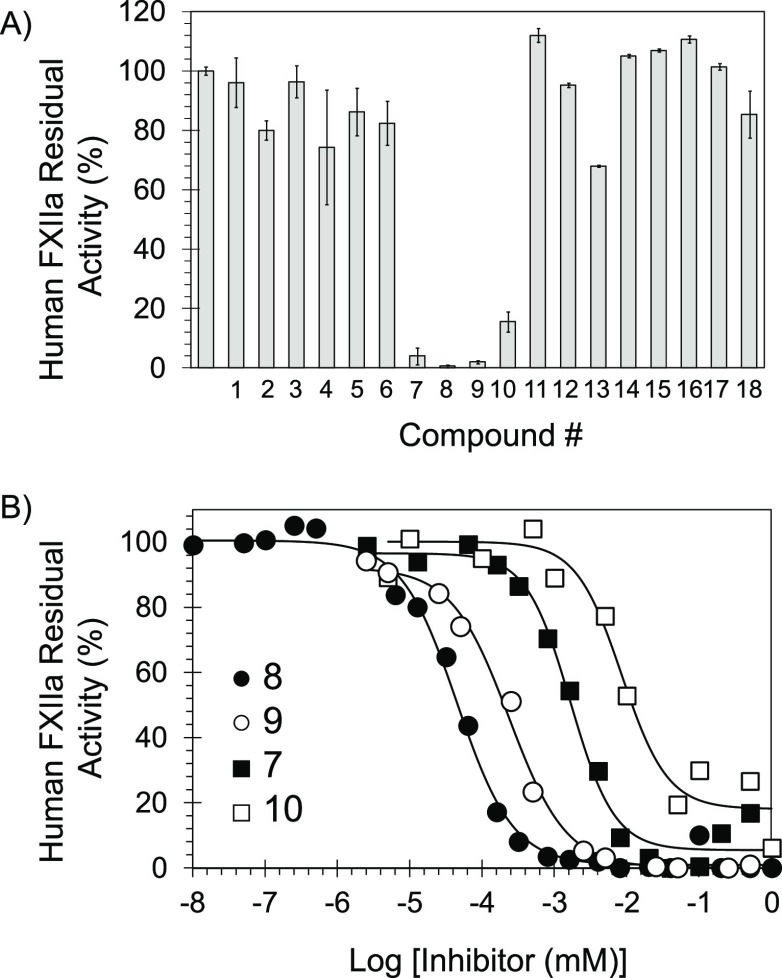
(A) Screening of the library of 18 compounds for FXIIa inhibition at 200 μM. (B) Direct inhibition of FXIIa by molecules **7**–**10** was studied using a chromogenic substrate assay under physiological conditions as described in Methods and Materials. Solid lines are the sigmoidal dose–response fits (eq 1) to the data to obtain the inhibition parameters.

The direct inhibition of FXIIa was assessed using a chromogenic substrate hydrolysis assay conducted under physiological conditions, specifically in a pH 7.4 Tris-HCl buffer at 37 °C, as previously documented.^40−47^ In this assay, the hydrolysis of the substrate by FXIIa results in a linear increase in absorbance at a wavelength of 405 nm. The slope of the resulting line corresponds to the residual enzyme activity, and the change in residual enzyme activity, relative to the concentration of the inhibitor, is plotted and fitted using the logistic eq 1. This analysis allows for the determination of potency (IC_50_), efficacy (Δ*Y* = *Y*_M_ – *Y*_0_), and Hill Slope (HS) of inhibition.^40−47^Figure 5A shows the inhibition of FXIIa at 200 μM by molecules **1**–**18**. Only four molecules **7**–**10** significantly inhibited human FXIIa. Thus, we measured the inhibition of FXIIa at different concentrations for each of the four molecules. Figure 5B shows the inhibition profiles for all four inhibitors. The IC_50_ values of these inhibitors were in the low micromolar and nanomolar ranges (Table 1) with efficacy (Δ*Y*%) values of more than 82%. The most potent among them were inhibitors **8** and **9** with IC_50_ values of 45 ± 3 and 240 ± 10 nM, respectively. Interestingly, none of the amino-thiazole-containing analogs (**1**–**6**), sulfonamide-containing analogs (**12** and **13**), or the free carboxylic acid-containing analogs (**14** and **15**) demonstrated any significant inhibitory activity at 200 μM (Figure 5A), potentially establishing the significance of the amino-triazole moiety and the central amide linkage as well as the detrimental effect of anionic group at position-3.

**Table 1:** Inhibition Parameters of Four Human FXIIa Inhibitors (**7**–**10**)^a^

inhibitor	IC_50_ (μM)	HS	Δ*Y* (%)
**RA**	0.11 ± 0.01^b^	1.7 ± 0.4	105 ± 8
**7**	1.7 ± 0.2	1.4 ± 0.2	91 ± 4
**8**	0.045 ± 0.003	1.1 ± 0.1	100 ± 2
**9**	0.24 ± 0.01	1.1 ± 0.1	92 ± 3
**10**	8.5 ± 1.2	1.4 ± 0.7	82 ± 8

a The inhibition parameters were acquired through nonlinear regression analysis of the direct inhibition of human FXIIa, conducted in suitable Tris–HCl buffers with a pH of 7.4 at 37 °C. Spectrophotometric monitoring was employed for tracking inhibition.

b The reported errors indicate ±1 standard error (S.E.).

We decided next to investigate the effect of the phenyl substituent on the overall effect of FXIIa inhibition potency. A series of halogenated analogs (**19**–**22**, Table 2) was synthesized. It was found that moving the chlorine substituent from the *ortho*-position (inhibitor **7**) to the *para*-position (inhibitor **19**) resulted in a significant decrease in the inhibition potency (∼4.6-fold). The IC_50_ values increased from 1.7 ± 0.2 to 7.8 ± 0.5 μM, respectively. Likewise, it was found that moving the bromine substituent from the *ortho*-position (inhibitor **20**) to the *para*-position (inhibitor **21**) resulted in a significant decrease in the inhibition potency (∼11.3-fold). The IC_50_ values increased from 0.08 ± 0.01 to 0.9 ± 0.1 μM, respectively. Comparing the inhibition potency of three *para*-substituted analogs [4-F (**22**), 4-Cl (**19**), and 4-Br (**21**)] suggests that the fluorine substituent demonstrates an optimal size and lipophilicity, resulting in an IC_50_ value of 32 ± 8 nM. Another way of looking at these results is that the para-position (position-4) favors small but lipophilic substituents. Altogether, inhibitor **22** is 3.4-fold more potent than our previously reported FXIIa inhibitor **RA.**(39) Furthermore, relative to inhibitor **9**, adding the fluorine substituent (similar in size to H, but more lipophilic) at the *para*-position of the phenyl ring resulted in a 7.5-fold increase in FXIIa inhibition potency.

**Table 2:**
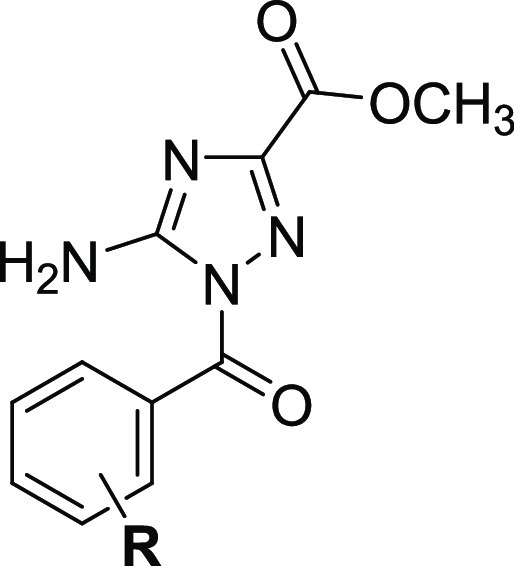
Inhibition Parameters of Human FXIIa Inhibitors (**19**–**22**)^a^

inhibitor	R	IC_50_ (μM)	HS	Δ*Y* (%)
**7**	2-Cl	1.7 ± 0.2^b^	1.4 ± 0.2	91 ± 4
**19**	4-Cl	7.8 ± 0.5	0.8 ± 0.1	102 ± 5
**20**	2-Br	0.08 ± 0.01	0.8 ± 0.1	76 ± 5
**21**	4-Br	0.9 ± 0.1	0.5 ± 0.02	109 ± 2
**22**	4-F	0.032 ± 0.008	0.8 ± 0.1	74 ± 7

a The inhibition parameters were acquired through nonlinear regression analysis of the direct inhibition of human FXIIa, conducted in suitable Tris–HCl buffers with a pH of 7.4 at 37 °C. Spectrophotometric monitoring was employed for tracking inhibition.

b The reported errors indicate ±1 SE.

To simplify our studies, we shifted our attention to the nonester analogs (**23**–**30**, Table 3). Although we previously established that the anionic carboxylate is detrimental to the activity, it was very surprising to identify that the ester functionality itself is not absolutely essential. This was well demonstrated by inhibitor **10,** which inhibited FXIIa with an IC_50_ value of 8.5 ± 1.2 μM, 77.3-fold less potent than inhibitor **RA**. As indicated in the ester-containing series, large substituents at the *para*-position (position-4) are not favorable; analogs with 4-methyl (inhibitor **23**), 3,4-dimethyl (inhibitor **27**), or 4-methoxy (inhibitor **28**) substituents demonstrated IC_50_ values of 7.7 ± 2.5, 2.3 ± 0.2, and 7.0 ± 1.6 μM, respectively. Furthermore, analogs with substituents at the *ortho*-position (position-2) also tend to be relatively weaker inhibitors: analogs with 2,5-dimethyl (inhibitor **25**) and 2,3-dimethoxy (inhibitor **29**) substituents demonstrated IC_50_ values of 9.0 ± 1.9 and 4.3 ± 0.3 μM, respectively. Accordingly, the most optimal positions that can tolerate substituents appear to be the *meta*-positions (position-3 and position-5) as demonstrated by inhibitor **24** (3-methyl substituent, IC_50_ = 0.6 ± 0.1 μM). In fact, adding another methyl substituent at the other meta-position (position-5) only decreased the potency by 2-fold as shown by inhibitor **26** (3,5-dimethyl substituents, IC_50_ = 1.2 ± 0.1 μM). Inhibitor **30**, which has two methoxy substituents at the *meta*-positions (3,5-dimethoxy substituents), further establishes the significant contribution of the meta-positions’ substituents to the potency of these molecules since it possesses an IC_50_ value of 0.34 ± 0.02 μM. Moreover, it appears that the methoxy substituents at the *meta*-positions are more favorable than the corresponding methyl substituents: inhibitor **30** is ∼3.5-fold more potent than inhibitor **26**. Considering these results (particularly, inhibitor **24**), an analog with one methoxy substituent may even possess better-estimated potency of 0.17 μM, but this remains to be tested.

**Table 3:**
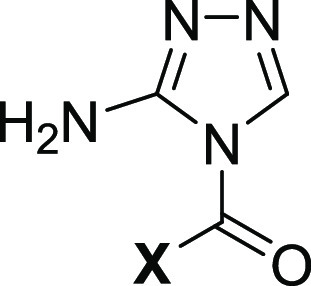
Inhibition Parameters of Human FXIIa Inhibitors (**23**–**30**)^a^

inhibitor	X	IC_50_ (μM)	HS	Δ*Y* (%)
**10**	4-*t*-butylphenyl	8.5 ± 1.2^b^	1.4 ± 0.7	82 ± 8
**23**	4-methylphenyl	7.7 ± 2.5	0.9 ± 0.2	94 ± 13
**24**	3-methylphenyl	0.6 ± 0.1	1.1 ± 0.2	70 ± 3
**25**	2,5-dimethylphenyl	9.0 ± 1.9	1.1 ± 0.2	75 ± 8
**26**	3,5-dimethylphenyl	1.2 ± 0.1	0.7 ± 0.1	104 ± 4
**27**	3,4-dimethylphenyl	2.3 ± 0.2	0.8 ± 0.1	104 ± 3
**28**	4-methoxyphenyl	7.0 ± 1.6	1.0 ± 0.2	97 ± 9
**29**	2,3-dimethoxylphenyl	4.3 ± 0.3	0.7 ± 0.1	110 ± 9
**30**	3,5-dimethoxyphenyl	0.34 ± 0.02	0.7 ± 0.0	94 ± 2

a The inhibition parameters were acquired through nonlinear regression analysis of the direct inhibition of human FXIIa, conducted in suitable Tris–HCl buffers with a pH of 7.4 at 37 °C. Spectrophotometric monitoring was employed for tracking inhibition.

b The reported errors indicate ±1 SE.

Lastly, analog **31** was synthesized to study the size effect of the ester functionality on the inhibition potency (Table 4). Analog **31**, which has ethyl ester, inhibited FXIIa with an IC_50_ value of 7.9 ± 1.9 μM, 33-fold less potent than analog **9**, which has methyl ester. Furthermore, analog **32** was synthesized to examine the significance of the aromatic ring for the inhibition potency (Table 4). Analog **33**, which has an acetyl group, inhibited FXIIa with an IC_50_ value of 6.3 ± 1.9 μM, 26.3-fold less potent than analog **9**, which has a benzoyl group. Overall, an amino-triazole core moiety is essential for FXIIa inhibition and is optimally required to be substituted with benzoyl moiety at position-1 and methyl ester at position-3. The benzoyl moiety is to be substituted only at one *meta*-position (preferably by methoxy) and at the *para*-position (preferably by fluorine).

**Table 4:**
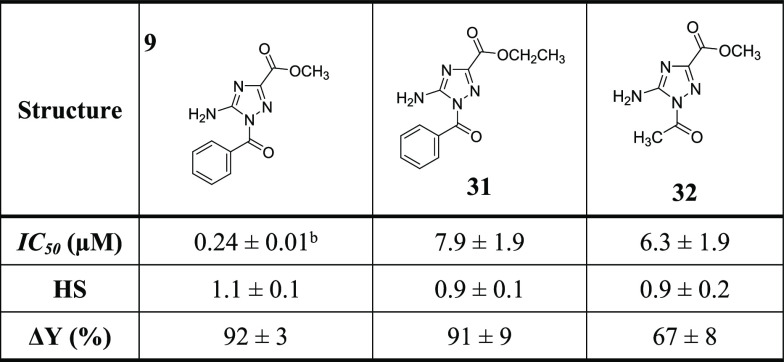
Inhibition Parameters of Human FXIIa Inhibitors (**31** and **32**)^a^

a The inhibition parameters were acquired through nonlinear regression analysis of the direct inhibition of human FXIIa, conducted in suitable Tris–HCl buffers with a pH of 7.4 at 37 °C. Spectrophotometric monitoring was employed for tracking inhibition.

b The reported errors indicate ±1 SE.

### Selectivity against Other Clotting Factors

Inhibitors **7**–**9**, **20**–**22**, **24**, **26**, and **30** were investigated for their potential to inhibit other serine proteases under physiological conditions using the corresponding chromogenic substrate hydrolysis assays, as documented earlier.^40−47^ The serine proteases included in the selectivity studies are listed in Table 5. The selectivity was evaluated against thrombin and factors IXa, Xa, and XIa. Four subclasses of inhibitors have emerged based on selectivity studies. Some of these molecules, such as **7**–**9**, **20**, and **21,** appear to be dual inhibitors of FXIIa as well as thrombin. Interestingly, inhibitors **22** and **24** are more specific inhibitors of FXIIa. Particularly, inhibitor **22** exhibits a selectivity index of 9.38 over thrombin and more than 1562 over FXa. Inhibitor **26**, however, is a dual inhibitor of FXIIa and FXIa. Inhibitor **30** was identified as a nonspecific inhibitor. Given the potency and selectivity, we believe that inhibitors **8** and **22** represent excellent lead inhibitors to be further developed as relatively safer anticoagulants, given that FXII(a)-targeting agents are linked to low bleeding risk.^1−4^

**Table 5:** Inhibition Profiles of Triazole-Based Inhibitors^a^

inhibitor	protease	IC_50_ (μM)	HS	Δ*Y* (%)	selectivity index (IC_50_ enzyme/IC_50_ FXIIa)
**7**	thrombin	0.44 ± 0.08^b^	1.2 ± 0.3	96.9 ± 4.4	0.26
	FXIIa	1.7 ± 0.2	1.4 ± 0.2	91.1 ± 3.8	1.00
	FXIa	25.9 ± 4.4	1.2 ± 0.2	95.8 ± 7.7	15.24
	FIXa	>200	ND^c^	ND	>117.64
	FXa	>200	ND	ND	>117.64
**8**	thrombin	0.019 ± 0.004	0.9 ± 0.2	102.0 ± 4.5	0.42
	FXIIa	0.045 ± 0.003	1.1 ± 0.1	99.7 ± 2.0	1.00
	FXIa	14.9 ± 1.4	0.9 ± 0.1	100.3 ± 3.1	331.11
	FIXa	>180	ND	ND	>4000
	FXa	47.6 ± 5.8	1.2 ± 0.2	100.8 ± 2.7	1057.78
**9**	thrombin	0.054 ± 0.008	1.1 ± 0.2	89 ± 5	0.23
	FXIIa	0.24 ± 0.01	1.1 ± 0.1	92 ± 3	1.00
	FXIa	>50	ND	ND	208.33
	FIXa	NA^d^	NA	NA	NA
	FXa	NA	NA	NA	NA
**20**	thrombin	0.09 ± 0.02	0.9 ± 0.1	113 ± 8	1.13
	FXIIa	0.08 ± 0.01	0.8 ± 0.1	76 ± 5	1.00
	FXIa	>50	ND	ND	*>*625
	FIXa	NA	NA	NA	NA
	FXa	NA	NA	NA	NA
**21**	thrombin	0.9 ± 0.2	0.5 ± 0.02	112 ± 10	1.00
	FXIIa	0.9 ± 0.1	0.5 ± 0.02	109 ± 2	1.00
	FXIa	>50	ND	ND	>55.56
	FIXa	NA	NA	NA	NA
	FXa	NA	NA	NA	NA
**22**	thrombin	0.30 ± 0.05	1.2 ± 0.2	118 ± 7	9.38
	FXIIa	0.032 ± 0.008	0.8 ± 0.1	74 ± 7	1.00
	FXIa	>50	ND	ND	>1562.50
	FIXa	NA	NA	NA	NA
	FXa	NA	NA	NA	NA
**24**	thrombin	43% at 50 μM	ND	ND	ND
	FXIIa	0.6 ± 0.1	1.1 ± 0.2	70 ± 3	1.00
	FXIa	>50	ND	ND	>83.33
	FIXa	NA	NA	NA	NA
	FXa	NA	NA	NA	NA
**26**	thrombin	>50	ND	ND	41.67
	FXIIa	1.2 ± 0.1	0.7 ± 0.1	104 ± 4	1.00
	FXIa	2.2 ± 0.7	0.6	73 ± 10	1.83
	FIXa	NA	NA	NA	NA
	FXa	NA	NA	NA	NA
**30**	thrombin	0.25 ± 0.14	1.0 ± 0.3	53 ± 16	0.74
	FXIIa	0.34 ± 0.02	0.7 ± 0.0	94 ± 2	1.00
	FXIa	2.4 ± 0.3	0.7	82 ± 5	7.06
	FIXa	NA	NA	NA	NA
	FXa	NA	NA	NA	NA

a The inhibition parameters were obtained following nonlinear regression analysis of direct inhibition of human enzyme in appropriate Tris–HCl buffers of pH 7.4 at 37 °C. Inhibition was monitored spectrophotometrically.

b Errors represent ±1 SE.

c Not determined.

d Not available.

### Anticoagulant Activity of New Triazole-Based Analogs in Human Plasma

**Figure 6:**
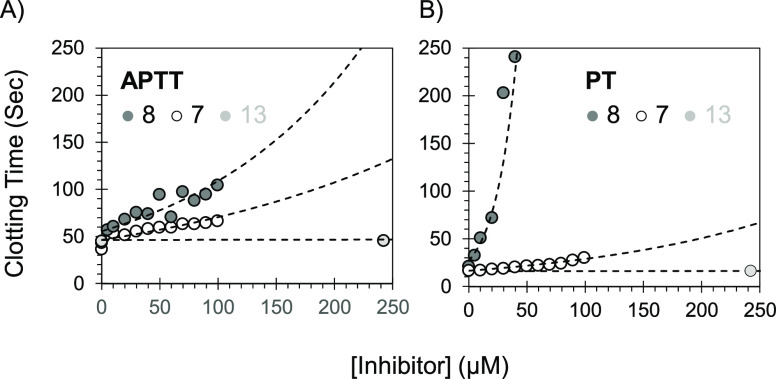
Effect of **7**, **8**, and **13** on APTT (A) and PT (B) in human plasma. Error bars represent ±1 SE (smaller than the symbol size). Clotting assays are described in the Methods and Materials.

Human plasma clotting assays are routinely exploited to investigate the anticoagulant activity of new enzyme inhibitors in an in vitro setting. While the APTT studies the effect of an inhibitor on the intrinsic coagulation pathway, the PT studies the effect on the extrinsic pathway of coagulation. Given the available quantities, the effects of molecules **7**, **8**, **13**, **26**, and **30** on APTT and PT were measured, as previously reported. Results are reported in Table 6 and Figure 6. Inhibitor **7** doubled the APTT at a concentration of ∼96.5 μM and the PT at ∼72.4 μM. Inhibitor **8** also doubled the APTT at a concentration of ∼58.8 μM and the PT at ∼7 μM. Nevertheless, molecule **13** did not affect the APTT or PT at the highest concentration of 2500 μM, further demonstrating the inactivity of the thiazole-based molecules. Several molecules were used as positive controls, including our previously reported FXIIa inhibitor **RA**. Relative to **RA**, inhibitor **8** demonstrated an increased potency (∼6-fold) regarding the effect on APTT. Interestingly, inhibitor **22** demonstrated selective yet variable effects in the APTT assay with concentrations in the range of 25–75 μM to double the APTT of human plasma (results are not shown).

**Table 6:** Effects of Clinical and Experimental Anticoagulants on APTT and PT in Human Plasma

anticoagulant	APTT _(EC×1.5)_^a^	PT_(EC×1.5)_^a^
**7**	∼96.5 μM^b^	∼72.4 μM
**8**	∼58.8 μM	∼7 μM
**13**	>2500 μM	>2500 μM
**26**	213.15 μM	>587 μM
**30**	301.4 μM	ND^c^
argatroban HCl	∼0.19 μM	∼0.13 μM
rivaroxaban	∼0.06 μM	∼0.08 μM
UFH	∼0.47 μg/mL	∼2 μg/mL
C6B7	∼0.06 μg/mL	NE^d^
RA	∼340 μM	NE

a The effective concentration to double the clotting time in the corresponding assay.

b Standard errors are less than 10% based on multiple measurements.

c Not determined.

d No effect at the highest concentration tested. EC: Effective concentration; AT: Antithrombin; APTT: Activated partial thromboplastin time; PT: Prothrombin time.

### Molecular Modeling Studies

**Figure 7:**
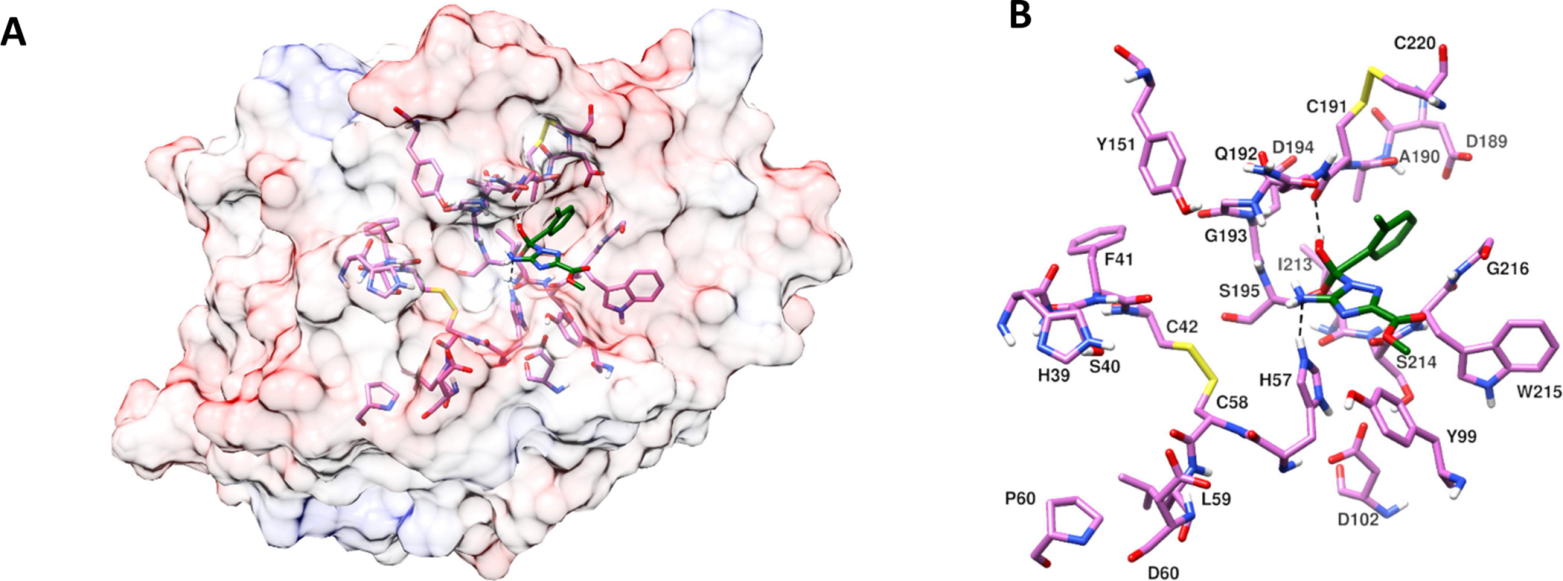
Predicted binding mode of inhibitor **7** in the active site of FXIIa. Amino acids in the binding pocket and nearby important amino acids are shown in stick model and H-bond interactions are shown with a black dashed line. (A) FXIIa in semitransparent surface representation; (B) FXIIa amino acids in the binding pocket and nearby important amino acids.

**Figure 8:**
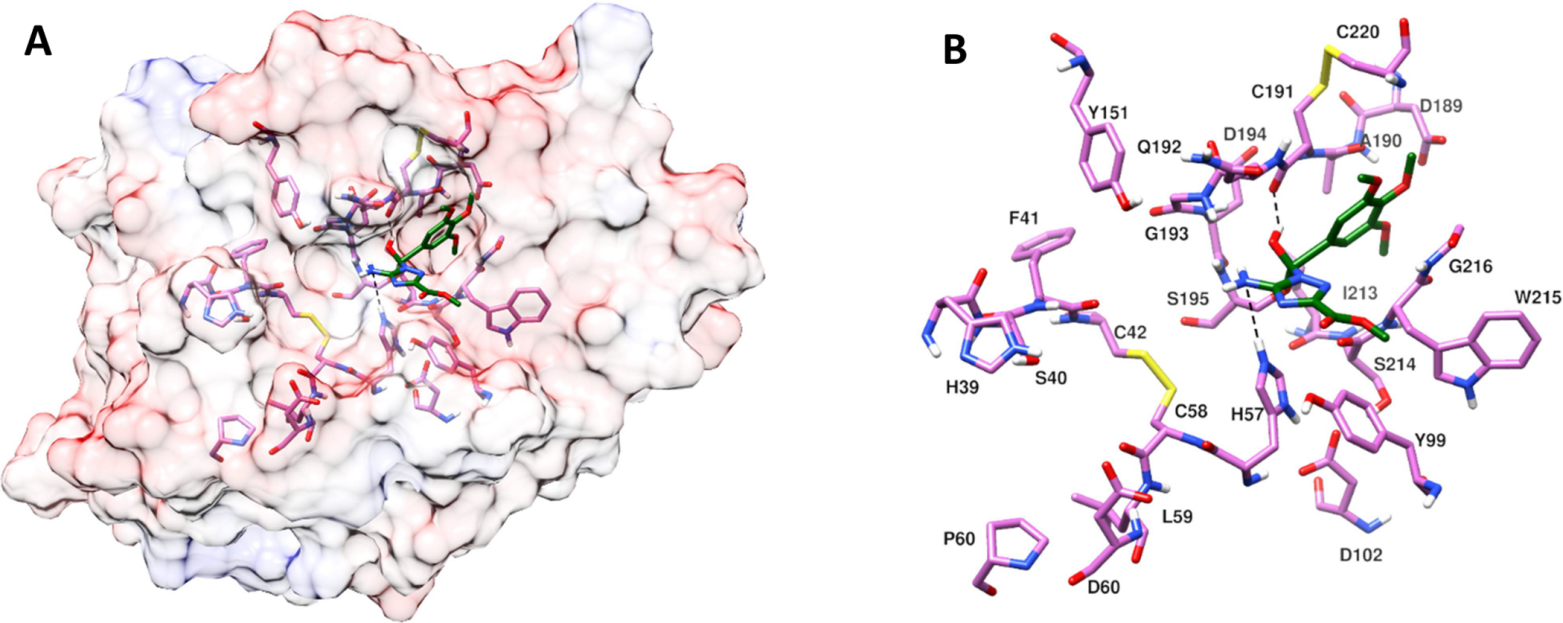
Predicted binding mode of inhibitor **8** in the active site of FXIIa. Amino acids in the binding pocket and nearby important amino acids are shown in the stick model, and H-bond interactions are shown with a black dashed line. (A) FXIIa in semitransparent surface representation; (B) FXIIa amino acids in the binding pocket and nearby important amino acids.

**Figure 9:**
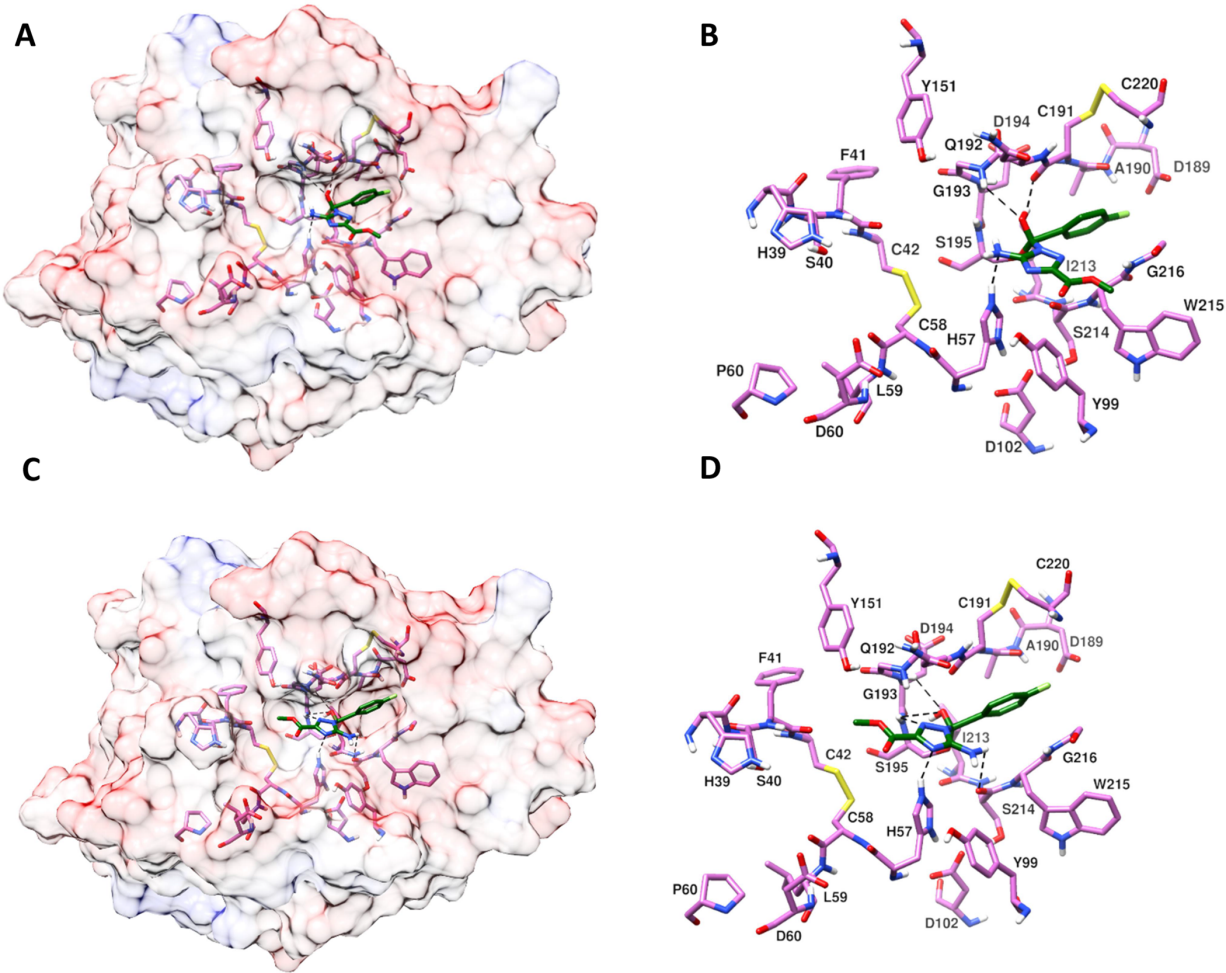
Predicted binding modes of inhibitor **22** in the active site of FXIIa. Amino acids in the binding pocket and nearby important amino acids are shown in stick model and H-bond interactions are shown with a black dashed line. (A and B) Triazole binding is similar to the triazole binding in **8**, where the amine attached to the triazole is oriented inward. (C and D) Triazole binding is different from **8**, where the amine attached to the triazole is oriented outward. (A and C) FXIIa in semitransparent surface representation; (B and D) FXIIa amino acids in the binding pocket and nearby important amino acids.

To identify the plausible binding mode of the selected inhibitors (**7**, **8,** and **22**), and their selectivity toward different proteases (FXIIa, FXIa, FXa, and Thrombin), we performed covalent docking studies using S195 as the reactive residue on the protein. All the calculations were done using the Schrödinger covalent docking procedure with nucleophilic addition to a double bond protocol. Binding modes of protease inhibitors in the active site of FXIIa comprising the catalytic triad residues H57, D102, and S195 for inhibitors **7**, **8,** and **22** are shown in Figures 7–9, respectively. Docking studies revealed that these compounds can potentially bind to FXIIa. Similarly, these inhibitors can also bind to other proteases, like FXIa, FXa, and thrombin (figures are not shown). In all the cases, the central amide carbonyl of these inhibitors was able to covalently bind to the reactive residue S195, and the phenyl ring bearing the ortho, meta, or para substitutions was found to bind to the same pocket on the protein. Likewise, the binding of the triazole group bearing the amine and methyl acetate groups is similar for all three inhibitors where the amine group is oriented inward and the methyl acetate group is oriented outward. In this conformation, the amine group on the triazole of all three inhibitors (**7**, **8,** and **22**) was found to make H-bond interactions with H57 and the hydroxyl group of **7**, **8,** and **22** resulted from the nucleophilic addition was found to make H-bond with the backbone carbonyl oxygen of C191 (See Figures 7–9: A and B). This hydroxyl group in **22** also makes an H-bond with the backbone carbonyl oxygen of G193. However, in the triazole group, which is in the flipped conformation of **22** (Figure 9: C and D), the amine group is oriented outward, and the methyl acetate group is oriented inward; the triazole of **22** was found to make H-bond interactions with H57 and the backbone NH of S195. In addition, the outward-oriented amine was found to make H-bond interactions with the backbone carbonyl oxygen of S214, and the hydroxyl group (resulting from nucleophilic addition) was found to make H-bond interactions with the amide NH of S195 and D194, making **22** in this conformation bind strongly to FXIIa.

Considering thrombin, inhibitor **8** was found to bind strongly when the triazole group was in the flipped conformation, and the nucleophilic addition was to the carbonyl of methyl acetate. Table 7 shows the experimental IC_50_ values and the binding free energy values obtained by the MM-GBSA calculations for inhibitors **7**, **8,** and **22** against four proteases, FXIIa, FXIa, FXa, and thrombin. Binding energy values show that for FXIIa, **22** is more potent than **7** and **8**, when the amine group attached to the triazole is oriented outward from the pocket by flipping the triazole group. Likewise, the binding energies for thrombin show that **8** is more potent than **22** and **7** when the triazole group is in the flipped conformation. The specificity of **22** against FXIIa and **8** against thrombin observed by the molecular modeling studies is broadly consistent with the experimental findings. Overall, these studies shed light on the binding mode of **8** which is more specific for thrombin and the binding mode of **22** which is more specific for FXIIa and subsequently can be used to further improve the design of the next triazole-based inhibitors of FXIIa.

**Table 7:** IC_50_ and Binding Free Energy Values of **7**, **8**, and **22** against FXIIa, FXIa, FXa, and Thrombin

protease	inhibitors					
	7	8	22	7	8	22
	IC_50_ (μM)			binding free energy (kcal/mol)		
FXIIa	1.7	0.045	0.032	–26.97	–38.88	–30.26 (−43.68)^a^
FXIa	25.9	14.9	>50	–28.9	–35.54	–24.00
FXa	>200	47.6	NA	–27.49	–24.51	–22.98
Thrombin	0.44	0.019	0.30	–30.31	–32.66 (−48.23)^a^	–36.58

a Triazole group is in the flipped conformation.

## Conclusions

Thrombosis accounts for one out of every four deaths globally, and the prevalence of this condition is expected to rise with the aging population.^17^ Anticoagulants play a crucial role in preventing and treating thrombosis, emphasizing the worldwide demand for agents that are both effective and safe. Studies indicate that the clotting process is always initiated with the FVIIa/TF complex of the extrinsic pathway (Figure 2). Given that human FXIIa belongs to the contact/intrinsic pathways interface, it primarily contributes to clotting amplification but not initiation. In addition, human FXIIa appears to primarily contribute to pathological clotting, i.e., thrombosis, and less so to physiological clotting, i.e., hemostasis. Thus, inhibition of FXIIa may fulfill the goal of anticoagulation without bleeding. Furthermore, although FXIIa is being considered in a few drug development programs, very few potent and selective small molecule inhibitors of FXIIa are in development. Therefore, the creation of small molecule inhibitors targeting FXIIa is anticipated to meet various unmet medical needs where current therapies exhibit limitations in terms of both efficacy and safety.

Despite the benefits of the existing anticoagulants, they are plagued with significant issues. These challenges encompass increased bleeding from specific anatomical sites, uncertain effectiveness of DOACs in certain patient groups, and ineffectiveness in others. Notably, gastrointestinal and genitourinary bleeding is a common occurrence with some DOACs. Additionally, while the risk of major bleeding is lower with DOACs than with warfarin, bleeding remains a prominent side effect. The apprehension about bleeding often results in the underutilization of DOACs in patients with AF. Patients with renal impairment face a higher risk of bleeding with both DOACs and warfarin compared to those with normal renal function. Unlike warfarin, DOACs undergo renal clearance, raising the potential for drug accumulation and bleeding complications in individuals with severe renal dysfunction. Additionally, a retrospective study based on claims data reported a lower incidence of major bleeding with apixaban compared to that with warfarin in patients with AF. However, no reduction in the risk of intracranial or gastrointestinal bleeding was observed with apixaban. Notably, the lower risk of stroke with apixaban compared to warfarin was identified only in patients receiving the higher dose apixaban regimen.^48^ Due to the lack of more comprehensive data on efficacy and safety, clinicians hesitate to prescribe DOACs for stroke prevention in AF patients with ESRD. Special populations where DOACs are contraindicated include individuals with mechanical heart valves^49^ and those diagnosed with antiphospholipid syndrome.^50,51^ Thus, there is a pressing need for safer anticoagulants, particularly those with minimal renal clearance and the capability to mitigate clotting induced by medical devices such as central venous catheters, heart valves, and extracorporeal circuits. Other potential indications include the prevention of major adverse events in patients with coronary or peripheral artery diseases, secondary stroke prevention in patients with noncardioembolic stroke, and vascular protection in ESRD patients.^52^

In this report, we identified a few FXIIa inhibitors, particularly **8** and **22**, to be considered in subsequent in vivo studies (venous thrombosis and tail bleeding in animal models) and efforts to develop effective and safer anticoagulants. They inhibited FXIIa with IC_50_ values of 45 and 32 nM, respectively. Inhibitor **22** also exhibited significant selectivity against thrombin as well as factors IXa, Xa, and XIa.

## Materials and Methods

### Chemicals, Reagents, and Analytical Chemistry

Anhydrous organic solvents (dichloromethane, hexanes, and ethyl acetate) were obtained from Fisher Scientific (Pittsburgh, PA) and used as they were received. 1-Ethyl-3-(3-(dimethylamino)propyl)carbodiimide (EDCI), 4-dimethylaminopyridine (DMAP), 1-hydroxy benzotriazole (HOBt), and molecules **11**–**18** were obtained from Milipore-Sigma (Burlington, MA). For analytical TLC, UNIPLATETM silica gel GHLF 250 μm precoated plates from ANALTECH, Newark, DE, were employed. Column chromatography utilized Sigma-Aldrich’s silica gel (200–400 mesh, 60 Å). All reactions were conducted in oven-dried glassware. Flash chromatography was carried out using Teledyne ISCO’s Combiflash RF system and disposable normal silica cartridges with a particle size of 30–50 μm, mesh size of 230–400, and pore size of 60 Å. The flow rate of the mobile phase was in the range of 18–35 mL/min, and mobile phase gradients of ethyl acetate/hexanes were used to elute inhibitors. Human plasmas were purchased from George King Bio-Medical, Inc. (Overland Park, KS). Reagents for clotting assays, including APTT reagent, thromboplastin D, and CaCl_2_ solution, were all from Fisher Scientific. UFHs were from Milipore-Sigma, whereas argatroban HCl, rivaroxaban, and C6B7 were from Fisher Scientific.

### Chemical Characterization of Synthesized Molecules

^1^H and ^13^C NMR spectra were recorded on a 500 MHz Bruker NMR spectrometer in DMSO-*d*_6_. Signals, in parts per million (ppm), are relative to the residual peak of the solvent. The NMR data are reported as chemical shift (ppm), multiplicity of signal (s = singlet, d = doublet, t = triplet, q = quartet, dd = doublet of doublet, m = multiplet), coupling constants (Hz), and integration. Mass profiles of synthesized molecules were obtained using a PerkinElmer PE-SCIEX API-150 mass spectrometer equipped with an electrospray ionization source. Positive and negative modes were both used. The elemental analysis was performed using PerkinElmer PE2400-Series II, CHNS/O analyzer for elemental analysis. The purity of synthesized molecules was greater than 95% based on NMR data as well as mass spectroscopy data.

### Proteins, Substrates, and Buffers

Human plasma serine proteases including thrombin, FXa, FXIa, and FIXa were obtained from Haematologic Technologies (Essex Junction, VT). FXIIa was purchased from Enzyme Research Laboratories (South Bend, IN). The substrates Spectrozyme TH, Spectrozyme FXa, and Spectrozyme FIXa were obtained from Biomedica-Diagnostics (Windsor, NS Canada). Factor XIIa (Chromogenix S-2302) and factor XIa (S-2366) substrates were obtained from Diapharma (West Chester, OH). All enzymes and substrates were prepared in 20–50 mM TrisHCl buffer, pH 7.4, containing 100–150 mM NaCl, 0.1% PEG8000, 2.5 mM CaCl_2_, and 0.02% Tween80. For FIXa buffer, 33% (v/v) ethylene glycol was also added.

### Synthesis of Ethyl (or Methyl) 2-Aminothiazole-5-carboxylate-Based Molecules (**1**–**6**)

The synthesis was carried out overnight in dichloromethane under basic conditions (triethylamine/n-methyl morpholine) according to either “method a” or “method b”. In “method a”, the reaction is between the thiazole (1 equiv) and benzoic acid derivatives (1 equiv) in the presence of EDCI (1.1 equiv) and HOBt (Scheme 1A). In this method, a round-bottom flask was dried with a heat gun, and a nitrogen balloon was placed on the flask through a rubber cap using syringe techniques. The flask was then charged with 7 mL of CH_2_CL_2_. The benzoic acid derivative was then added to the flask and stirred with a spin bar until it was completely dissolved. The acid derivative may not dissolve until the base catalyst has been added. The base catalyst was added to the solution dropwise by using a syringe. EDCI was then added to the reaction solution and was followed by the addition of HOBt. The thiazole-amine is last added, and the reaction is left to run overnight while stirring at room temperature. To work up the reaction, the reaction mixture was diluted with about 5 mL of CH_2_CL_2_, and a TLC was performed with a 50/50 mixture of ethyl acetate and hexanes. The solution was extracted with H_2_O followed by 1 M HCl, and another water wash was performed. Then, the solution was treated with a saturated NaHCO_3_ solution, followed by a final water wash. The solution was then dried over anhydrous sodium sulfate, and gravity filtration was performed to separate the drying agent. The solution was then concentrated using a rotary evaporator and purified using flash chromatography with solvents ethyl acetate and hexanes.

In “method b”, the reaction is between the thiazole (1 equiv) and benzoyl chlorides (1 equiv). In this method, a round-bottom flask was dried with a heat gun, and a nitrogen balloon was connected using a syringe pierced through a rubber stopper. Then, about 7 mL of CH_2_CL_2_ was charged to the flask. The thiazole-amine was then added to the reaction flask and stirred until it was completely dissolved. The dissolved catalyst was added dropwise with the use of a syringe. The addition of a catalyst may help dissolve stubborn reagents. The solution was cooled with an ice bath, and the benzoyl chloride was added slowly dropwise. If the benzoyl chloride was a solid, it was dissolved in a minimal amount of dichloromethane and added dropwise as stated before. The solution was then allowed to warm to room temperature and run overnight. To work up the reaction, the reaction mixture is diluted with 5 mL of CH_2_CL_2_ and extracted with an initial water wash. Then, the reaction mixture was treated with 10 mL of 1 M HCl, followed by another water wash. The reaction solution was treated using a saturated NaHCO_3_ solution, followed by a final water wash. The solution was dried using anhydrous sodium sulfate, and gravity filtration was used to separate the wet–drying agent. The solution was loaded onto a silica gel column for purification by flash chromatography using solvents ethyl acetate/hexanes. Generally, the abovementioned methods resulted in isolated yields of 40–70%.

### Synthesis of Methyl 5-Amino-1*H*-1,2,4-triazole-3-carboxylate-Based Molecules (**7** and **8**) and 1*H*-1,2,4-Triazol-5-amine Molecules (**9** and **10**)

The synthesis of **7** and **8** is depicted in Scheme 1B, whereas the synthesis of **9** and **10** is depicted in Scheme 1C. The synthesis was carried out following “method c”. In this method, the synthesis was carried out overnight in dichloromethane under basic conditions (DMAP). It involves a reaction between the triazole and benzoic acid derivatives in the presence of EDCI, as was mentioned in an earlier report.^39^

Following are the chemical characterizations of the synthesized molecules.

#### Ethyl 2-(4-Methyl-3-nitrobenzamido)thiazole-5-carboxylate (**1**)

^1^H NMR (DMSO-*d*_6_): 8.69 (s, 1H), 8.29 (d, 1H), 8.10 (s, 1H), 7.59 (d, 1H), 4.32 (q, 2H), 2.51 (s, 3H), 1.38 (t, 3H). ^13^C NMR (DMSO-*d*_6_): 164.10, 161.57, 159.03, 149.24, 141.64, 138.17, 133.87, 132.94, 131.14, 124.65, 123.86, 61.27, 20.13, 14.60.

#### Ethyl 2-(4-Methylbenzamido)thiazole-5-carboxylate (**2**)

^1^H NMR (DMSO-*d*_6_): 8.07 (s, 1H), 7.99 (d, 2H), 7.35 (d, 2H), 4.33 (q, 2H), 2.25 (s, 3H), 1.38 (t, 3H). ^13^C NMR (DMSO): 166.01, 161.65, 159.27, 141.54, 137.3, 131.10, 129.72, 129.11, 128.64, 123.59, 61.23, 21.51, 14.60.

#### Ethyl 2-(4-(Trifluoromethyl)benzamido)thiazole-5-carboxylate (**3**)

^1^H NMR (DMSO-*d*_6_): 13.29 (s, 1H), 8.29 (d, 2H), 8.15 (s, 1H), 7.95 (d, 2H), 4.32 (q, 2H), 1.38 (t, 3H). ^13^C NMR (DMSO-*d*_6_): 164.45, 161.41, 158.53, 140.90, 135.41, 129.15, 125.59, 123.44, 61.23, 14.60.

#### Methyl 2-(4-Methyl-3-nitrobenzamido)thiazole-5-carboxylate (**4**)

^1^H NMR (DMSO-*d*_6_): 13.31 (s, 1H), 8.73 (s, 1H), 8.31 (d, 1H), 8.14 (s, 1 H), 7.67 (d, 1 H), 3.84 (s, 3 H), 2.59 (s, 3H). ^13^C NMR (DMSO-*d*_6_): 163.38, 161.38, 158.47, 148.78, 140.86, 137.66, 133.34, 132.42, 130.61, 124.26, 123.41, 51.91, 19.71. Calculated MS [M + Na]^+^ = 344.03. Found MS [M + Na]^+^ = 344.10. Calculated MS [M – H]^−^ = 320.03. Found MS [M – H]^−^ = 319.80. Elemental Analysis: (Calculated) C, 48.60; H, 3.45; N, 13.08; S, 9.98; (found) C, 48.15; H, 3.33; N, 12.89; S, 9.35.

#### Methyl 2-(4-(Trifluoromethyl)benzamido)thiazole-5-carboxylate (**5**)

^1^H NMR (DMSO-*d*_6_): 13.29 (s, 1H), 8.29 (d, 2H), 8.15 (s, 1H), 7.95 (d, 2H), 3.83 (s, 3 H). ^13^C NMR (DMSO-*d*_6_): 164.45, 161.41, 158.53, 140.90, 135.41, 129.15, 125.59, 123.44, 51.91. Calculated MS [M + H]^+^ = 331.04. Found MS [M + H]^+^ = 330.90. Calculated MS [M – H]^−^ = 329.02. Found MS [M – H]^−^ = 328.80. Elemental Analysis: (Calculated) C, 47.28; H, 2.75; N, 8.48; S, 9.71; (found) C, 47.14; H, 2.38; N, 8.03; S, 8.33.

#### Methyl 2-(3,5-Dinitrobenzamido)thiazole-5-carboxylate (**6**)

^1^H NMR (DMSO-*d*_6_): 8.45 (s, 1H), 8.33 (d, 1H), 8.33 (d, 1H), 8.18 (d, 1H), 7.95(d, 1H), 3.81 (s, 3H). ^13^C NMR (DMSO-*d*_6_):164.91, 161.87, 158.99, 141.35, 135.87, 130.53, 129.59, 126.04, 123.87, 122.84, 52.35.

#### Methyl 5-Amino-1-(2-chlorobenzoyl)-1H-1,2,4-triazole-3-carboxylate (**7**)

^1^H NMR (DMSO-*d*_6_): 8.01 (br, s, 2H), 7.73 (dd, 1H), 7.64–7.59 (m, 2H), 7.53–7.50 (m, H), 3.77 (s, 3H). ^13^C NMR (DMSO-*d*_6_): 166.81, 159.83, 158.07, 153.09, 132.96, 132.49, 129.79, 129.51, 127.18, 52.43. Calculated MS [M]^+^ = 280.04. Found MS [M]^+^ = 280.80. Calculated MS [M + OH]^−^ = 297.04. Found MS [M + OH]^−^ = 296.9. Elemental Analysis: (Calculated) Elemental Analysis: C, 47.07; H, 3.23; N, 19.96; (found) C, 46.64; H, 2.78; N, 19.65. See Supporting Information for spectra.

#### Methyl 5-Amino-1-(3,4,5-trimethoxybenzoyl)-1H-1,2,4-triazole-3-carboxylate (**8**)

^1^H NMR (DMSO-*d*_6_): 7.85 (br, s, 2H), 7.44 (s, 2H), 3.83 (s, 3H), 3.82 (s, 6H), 3.79 (s, 3H). ^13^C NMR (DMSO-*d*_6_): 166.98, 160.06, 158.93, 152.27, 152.15, 141.99, 126.01, 109.02, 60.24, 56.14, 52.43. Calculated MS [M + H]^+^ = 337.11. Found MS [M + H]^+^ = 337.20. Calculated MS [M – H]^−^ = 335.10. Found MS [M – H]^−^ = 334.90. Elemental Analysis: (Calculated) C, 50.00; H, 4.80; N, 16.66; (found) C, 49.33; H, 4.17; N, 16.43. See Supporting Information for spectra.

#### Methyl 5-Amino-1-benzoyl-1H-1,2,4-triazole-3-carboxylate (**9**)

^1^H NMR (400 MHz, DMSO-*d*_6_): 7.98 (d, 2H, *J* = 7.4 Hz), 7.69–7.67 (m, 1H), 7.57–7.54 (m, 2H), 3.81 (s, 3 H).^13^C NMR (100 MHz, DMSO-*d*_6_): 168.08,160.02, 158.63, 152.31, 133.33, 131.27, 130.50, 128.17, 52.47. Calculated MS [M + Na]^+^ = 269.07. Found MS [M + Na]^+^ = 269.05.

#### (5-Amino-1H-1,2,4-triazol-1-yl)(4-(*tert*-butyl)phenyl)methanone (**10**)

^1^H NMR (400 MHz, DMSO-*d*_6_): 8.02 (d, 2H, *J* = 8.48 Hz), 7.67 (s, 1H), 7.63 (s, 1H), 7.58 (d, 2H, *J* = 8.48 Hz), 1.33 (s, 9H). ^13^C NMR (100 MHz, DMSO-*d*_6_): 167.59, 158.32, 156.13, 151.24, 130.72, 129.12, 124.85, 34.84, 30.76. Calculated MS [M + Na]^+^ = 267.12. Found MS [M + Na]^+^ = 267.09.

#### Methyl 5-Amino-1-(4-chlorobenzoyl)-1H-1,2,4-triazole-3-carboxylate (**19**)

^1^H NMR (DMSO-*d*_6_, 400 MHz): 8.04 (d, *J* = 8.6 Hz, 2H), 7.89 (br s, 2H, D_2_O exchangeable), 7.66 (d, *J* = 8.6 Hz, 2H), 3.82 (s, 3H); EI-MS *m*/*z* (M + H^+^) = 280.96.

#### (3-Amino-4H-1,2,4-triazol-4-yl)(2,5-dimethylphenyl)methanone (**25**)

^1^H NMR (DMSO-*d*_6_, 400 MHz): 7.69 (br, s, 2H), 7.52 (s, 1H), 7.29 (s, 1 H), 7.26 (d, 1 H), 7.20 (d, 1H), 2.3 (s, 3H), 2.2 (s, 3H). ^13^C NMR (DMSO-*d*_6_): 170.23, 158.15, 152.09, 134.84, 133.91, 132.77, 131.77, 130.62, 128.86, 20.80, 19.15. EI-MS *m*/*z* (M + Na^+^) = 239.1.

#### (3-Amino-4H-1,2,4-triazol-4-yl)(3,4-dimethylphenyl)methanone (**27**)

^1^H NMR (DMSO-*d*_6_, 400 MHz): 7.85 (s, 1H), 7.82 (d, 1H), 7.65 (br, s, 2 H), 7.62 (s, 1 H), 7.30 (d, 1H), 2.31 (s, 3 H), 2.30 (s, 3H). ^13^C NMR (DMSO-*d*_6_): 168.18, 158.82, 151.71, 142.83, 136.56, 132.02, 129.86, 129.55, 129.01, 20.05, 19.80. EI-MS *m*/*z* (M + Na^+^) = 239.1.

#### Synthesis of Methyl 1-Acetyl-5-amino-1H-[1,2,4]triazole-3-carboxylate (**32**)

A suspension of methyl 5-amino-1H-[1,2,4]triazole-3-carboxylate (1 mmol) in acetic anhydride (11 mmol) was stirred for 5 h, and volatiles were evaporated to afford the corresponding product. ^1^H NMR (DMSO-*d*_6_): 7.69 (s, 2H, NH2), 3.87 (s, 3H), 2.60 (s, 3H). ^13^C NMR (DMSO-*d*_6_): 171.4, 159.7, 157.1, 151.8, 52.0, 22.7. EI-MS *m*/*z* (M + H^+^) = 185.1.

### Direct Inhibition of Human FXIIa

The direct inhibition of human FXIIa was assessed through a chromogenic substrate hydrolysis assay, employing a microplate reader under physiological conditions of 37 °C and pH 7.4, consistent with our previous reports.^40−47^ In this setup, each well of the 96-well microplate contained 175 μL of pH 7.4 TrisHCl buffer, to which 5 μL of potential inhibitors (or vehicle) and 5 μL of human FXIIa (stock concentration: 200 nM) were added sequentially. After a 5 min incubation, 5 μL of the FXIIa substrate (stock: 5 mM) was rapidly introduced, and the residual FXIIa activity was gauged based on the initial rate of absorbance increase at a wavelength of 405 nm. Potential FXIIa inhibitors were prepared in different concentrations in the wells, ranging from 500 to 0.0025 μM. The relative residual FXIIa activity at each inhibitor concentration was computed by comparing the FXIIa activity in the absence and presence of the inhibitor. To obtain the potency (IC_50_) and efficacy (Δ*Y*) of inhibition, the dose dependence of residual FXIIa activity was fitted to the logistic eq 1.
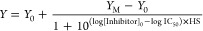
1

In the provided equation, *Y* is the ratio of residual FXIIa activity in the presence of inhibitors to that in its absence, *Y*_0_ and *Y*_M_ are the minimum and maximum possible values of the fractional residual proteinase activity, IC_50_ is the concentration of the inhibitor that results in 50% inhibition of enzyme activity, and HS is the Hill slope. Nonlinear curve fitting resulted in the IC_50_, HS, *Y*_0_, and *Y*_M_ values.

### Inhibition of Other Serine Proteases

The inhibition profiles of several inhibitors against clotting serine proteases including factors IIa, IXa, Xa, and XIa were determined using the corresponding chromogenic substrate hydrolysis assays, as documented in our previous studies.^40−47^ These assays were performed by using substrates and conditions appropriate for the enzyme being studied. To conduct selectivity analysis, various concentrations of the inhibitors were utilized, and the residual enzyme activity was assessed at each concentration. If the enzyme exhibited concentration-dependent inhibition, a profile of concentration versus relative residual enzyme activity (%) was generated using logistic eq 1. This allowed for the determination of the corresponding IC_50_ and efficacy of the enzyme–inhibitor complex. The *K*_M_ of the chromogenic substrate with its enzyme was employed to ascertain the concentration of the chromogenic substrate used in the inhibition studies. The concentrations of enzymes and substrates in microplate cells were about 6 and 50 μM for thrombin, 89 and 850 μM for FIXa, and 1.09 and 125 μM for FXa.

### Effects on Clotting Times of Normal Human Plasma

Enzyme inhibitors’ anticoagulant activity is frequently examined through plasma clotting assays. The APTT assay is employed to assess the impact of a new potential anticoagulant on clotting driven by the contact/intrinsic pathway, involving FIXa, FXIa, and FXIIa. Conversely, the PT assay is utilized to gauge the impact of the new potential anticoagulant on the extrinsic pathway of coagulation, which includes FVIIa. These experiments were performed using the BBL Fibrosystem fibrometer from Becton–Dickinson, Sparles, MD, USA, as detailed in our previous studies.^40−47^ In the PT assay, thromboplastin-D was created by adding 4 mL of distilled water, and the resulting mixture was warmed to 37 °C. Subsequently, a 90 μL volume of citrated human plasma was combined with 10 μL of the molecule of interest (or the vehicle) and incubated for 30 s at 37 °C. Upon the addition of 200 μL of prewarmed thromboplastin-D reagent, the clotting time was recorded. In the APTT assay, 90 μL of citrated human plasma was combined with 10 μL of the molecule of interest (or the vehicle) and 100 μL of prewarmed 0.2% ellagic acid. Following a 4 min incubation at 37 °C, clotting was initiated by introducing 100 μL of prewarmed 0.025 M CaCl_2_, and the clotting time was recorded. In both assays, approximately nine or more concentrations of molecules were employed to generate concentration vs effect profiles. The data were then plotted against a quadratic trendline, enabling the estimation of the concentration required to double the clotting time. Clotting times were similarly measured using 10 μL of highly purified water (negative control). The positive controls used in APTT and PT assays were (1) UFH, antithrombin activator; (2) argatroban, a clinically used thrombin inhibitor; (3) rivaroxaban, a clinically used FXa inhibitor; and (4) C6B7, a monoclonal antibody FXIIa inhibitor.

### Molecular Docking Studies

Crystal structures of the proteases used in the modeling studies were obtained from the RSCB database (PDB IDs: 6B77/FXIIa, 2FDA/FXIa, 1FAX/FXa, and 1D3T/thrombin). All the structures were processed with the Protein Preparation Wizard in the Schrödinger Suite 2023–4 by removing all the ions and crystallographic water molecules, followed by adding hydrogen atoms consistent with a physiological pH of 7.^53^ Then, the protein–ligand complexes were energy-minimized with an RMSD cutoff value of 0.3 Å for all heavy atoms. Structures of inhibitors **7**, **8,** and **22** were prepared using the Builder module of Schrödinger followed by energy minimization.^55^ The active site, made up of the catalytic triad H57, D102, and S195, backed up by S214, was selected as the ligand binding site. The center of the receptor grids for each target protein (FXIIa, FXIa, FXa, or thrombin) was placed at the centroid of active site residues in a cubic grid box. Covalent docking simulations were carried out using the Schrödinger covalent docking procedure with the nucleophilic addition to a double bond protocol.^54^ In all cases, Ser195 was selected as the reactive residue on the protein to which ligands are attached. Finally, the binding free energies of the docked complexes were obtained by performing MM-GBSA calculations.

## Supporting Information Available

Chemical characterization of the synthesized molecules and NMR and mass spectra (PDF)

## Abbreviations

APC: Activated protein C
APTT: Activated partial thromboplastin time
DMAP: 4-dimethylaminopyridine
EDCI: 1-Ethyl-3-(3-(dimethylamino)propyl)carbodiimide
FXa: Factor Xa
FXIa: Factor XIa
FIXa: Factor IXa
FXIIa: Factor XIIa
FVa: Factor Va
HK: High molecular weight kininogen
NETs: Neutrophil extracellular traps
PolyP: Polyphosphate
PT: Prothrombin time
TF: Tissue factor
TM: Thrombomodulin
