# Supplementary material for: New Triazole-Based Potent Inhibitors of Human Factor XIIa as Anticoagulants
Source: ACS Omega. 2024 Feb 22;9(9):10694–708. doi: 10.1021/acsomega.3c09335 (PMC10918664; doi:10.1021/acsomega.3c09335)
Supplement: Supplementary file 1 — ao3c09335_si_001.pdf [file ao3c09335_si_001.pdf]

Supplementary Information

New Triazole-Based Potent Inhibitors of Human Factor XIIa as Anticoagulants

Ma'Lik D Woodland,[1] Anthony Thompson,[2] Amanda Lipford,[2] Navneet Goyal,[2] John C. Schexnaildre,[1] Madhusoodanan Mottamal,[2] Daniel K. Afosah,[3] Rami A. Al-Horani[1*]

[1]*Division of Basic Pharmaceutical Sciences, College of Pharmacy, Xavier University of Louisiana, New Orleans LA 70125;* mwoodlan@xula.edu, ralhoran@xula.edu

[2]*Department of Chemistry, Xavier University of Louisiana, New Orleans, LA 70125;* ngoyal@xula.edu

[3]*Department of Medicinal Chemistry, School of Pharmacy, Virginia Commonwealth University, Richmond VA 23219;* afosahd@vcu.edu

* Address for correspondence: Dr. Rami A. Al-Horani, 1 Drexel Drive, College of Pharmacy, New Orleans, LA 70125-1089. Phone: (504) 520-7603, Fax: (504) 520-7954, Email: ralhoran@xula.edu

Figures

Figure	Page
**Figure S1.** ^{1}H NMR of Molecule (4).	S4
**Figure S2.** ^{13}C NMR of Molecule (4).	S6
**Figure S3.** Mass Spectroscopy of Molecule (4).	S7
**Figure S4.** ^{1}H NMR of Molecule (5).	S9
**Figure S5.** ^{13}C NMR of Molecule (5).	S11
**Figure S6.** Mass Spectroscopy of Molecule (5).	S12
**Figure S7.** ^{1}H NMR of Molecule (7).	S14
**Figure S8.** ^{13}C NMR of Molecule (7).	S16
**Figure S9.** Mass Spectroscopy of Molecule (7).	S17
**Figure S10.** ^{1}H NMR of Molecule (8).	S19
**Figure S11.** ^{13}C NMR of Molecule (8).	S21
**Figure S12.** Mass Spectroscopy of Molecule (8).	S22
**Figure S13.** ^{1}H NMR of Molecule (19).	S23
**Figure S14.** Mass Spectroscopy of Molecule (19).	S23
**Figure S15.** ^{1}H NMR of Molecule (25).	S24
**Figure S16.** ^{13}C NMR of Molecule (25).	S25
**Figure S17.** ^{1}H NMR of Molecule (27).	S26
**Figure S18.** ^{13}C NMR of Molecule (27).	S27

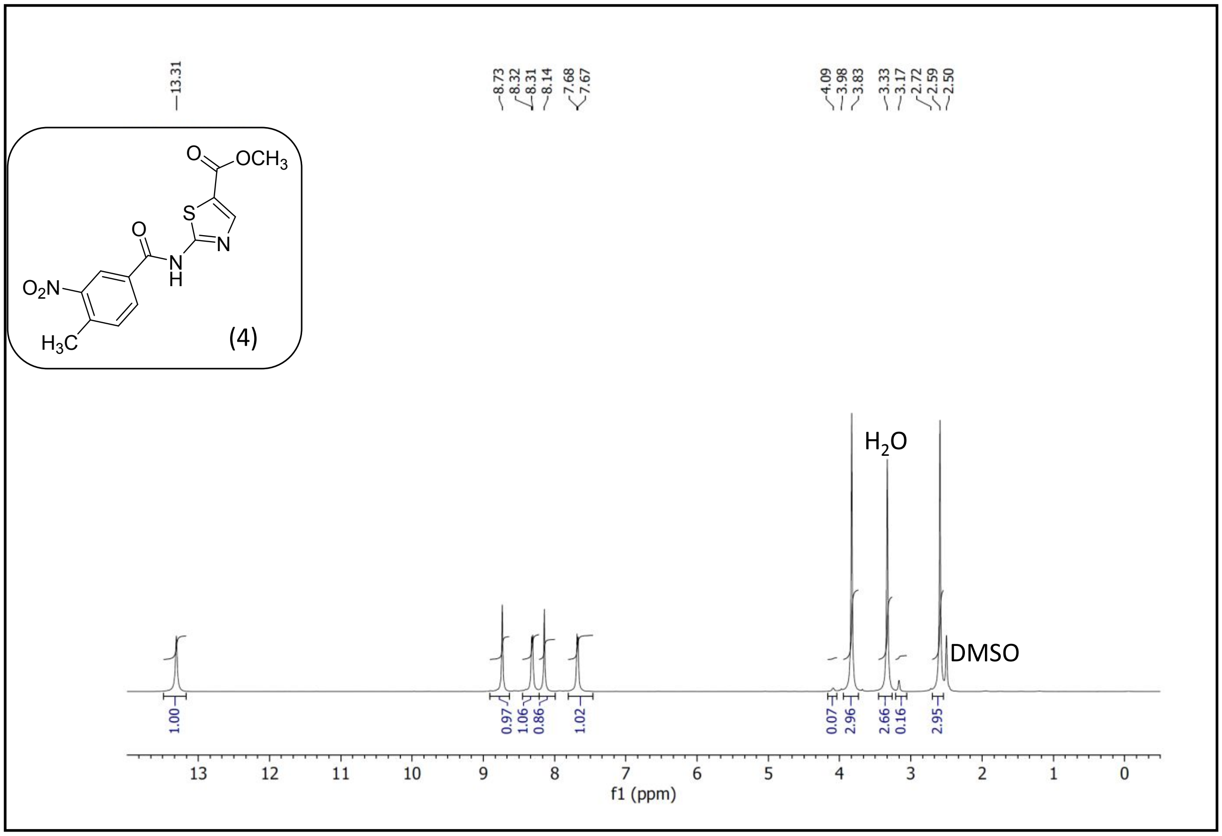
13.31
8.73
8.32
8.31
8.14
7.68
7.67
4.09
3.98
3.83
3.33
3.17
2.72
2.59
2.50
OCH3
S
N
O
NH
O2N
H3C
(4)
H2O
DMSO
1.00
0.97
1.06
0.86
1.02
0.07
2.96
2.66
0.16
2.95
f1 (ppm)

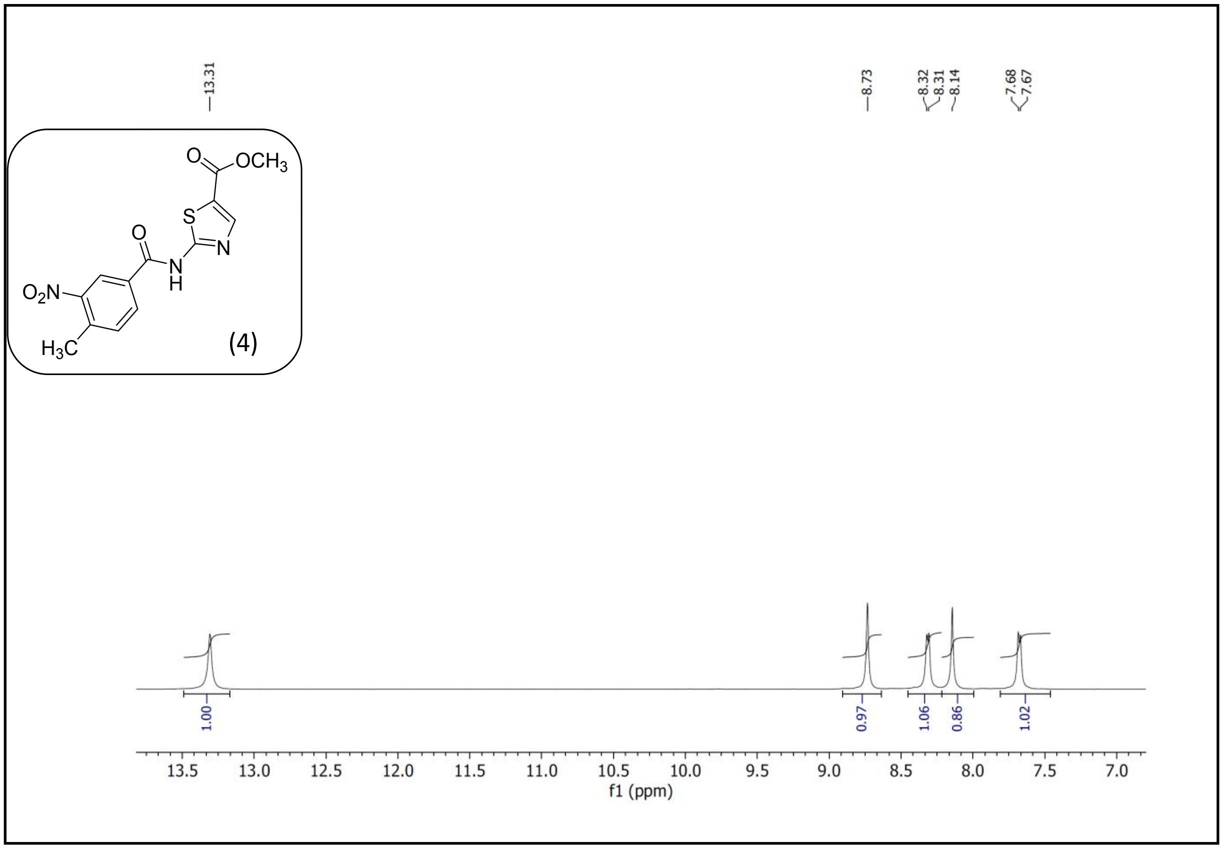
13.31
8.73
8.32
8.31
8.14
7.68
7.67
OCH3
S
N
O
NH
O2N
H3C
(4)
1.00
0.97
1.06
0.86
1.02
f1 (ppm)

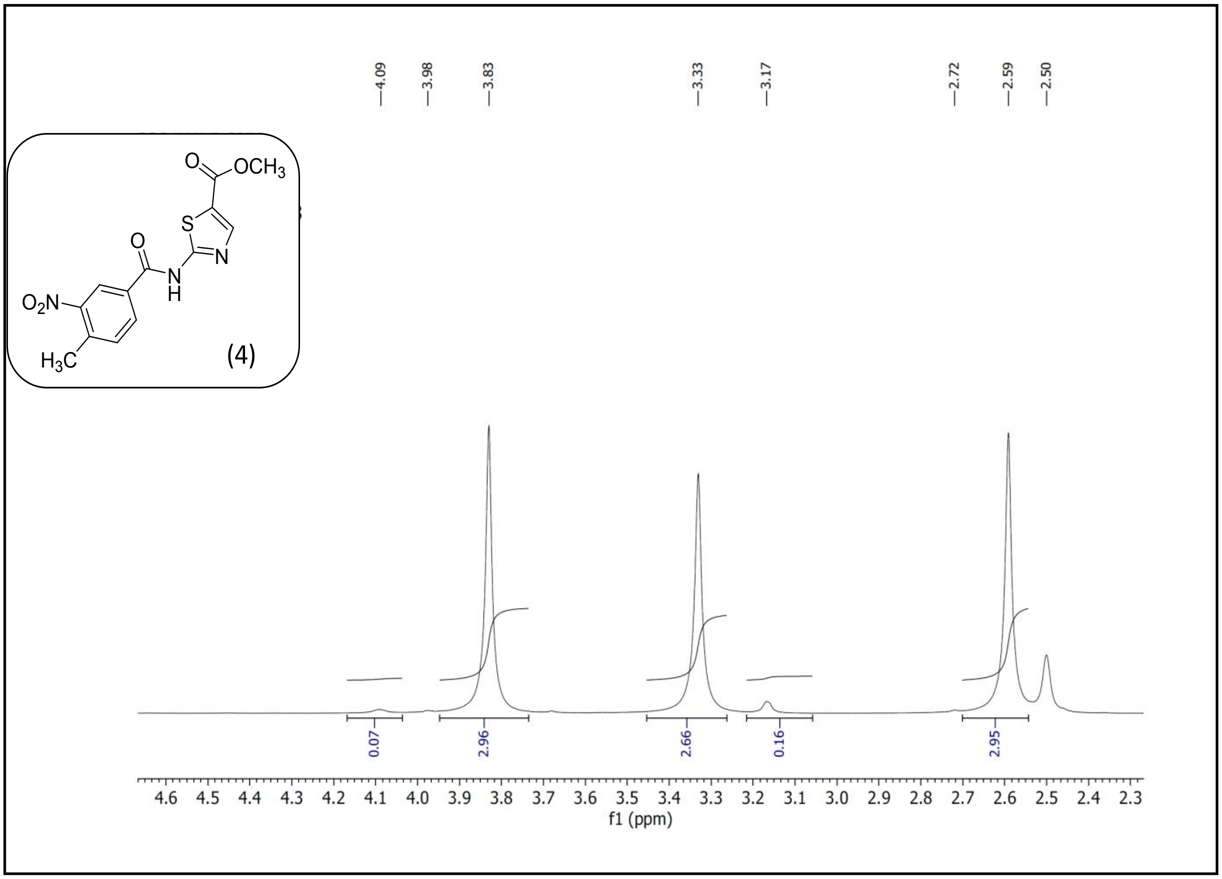

Figure S1. ^{1}H NMR of Molecule (4).

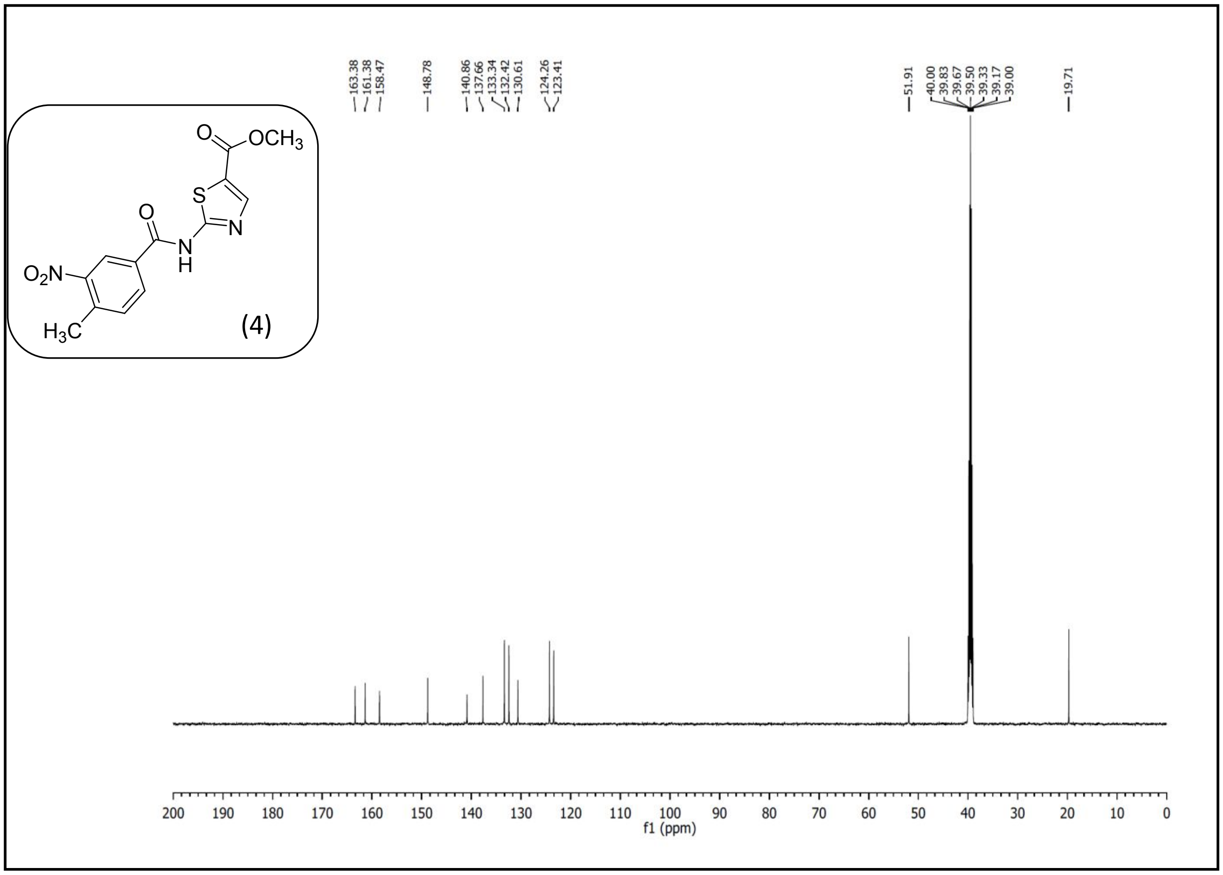
163.38
161.38
158.47
148.78
140.86
137.66
133.34
132.42
130.61
124.26
123.41
51.91
40.00
39.83
39.67
39.50
39.33
39.17
39.00
19.71
OCH3
O2N
H3C
(4)
f1 (ppm)

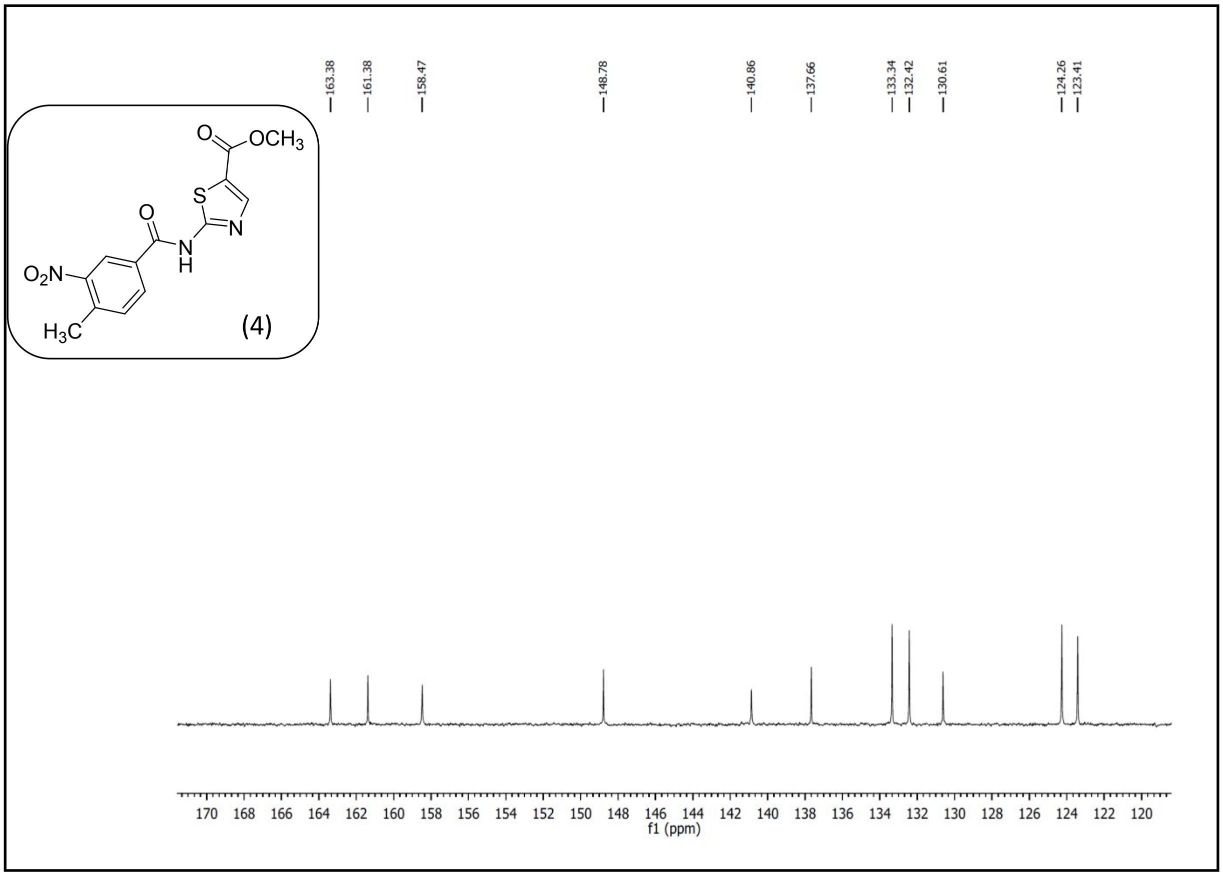
163.38
161.38
158.47
148.78
140.86
137.66
133.34
132.42
130.61
124.26
123.41
OCH3
O2N
H3C
(4)
f1 (ppm)

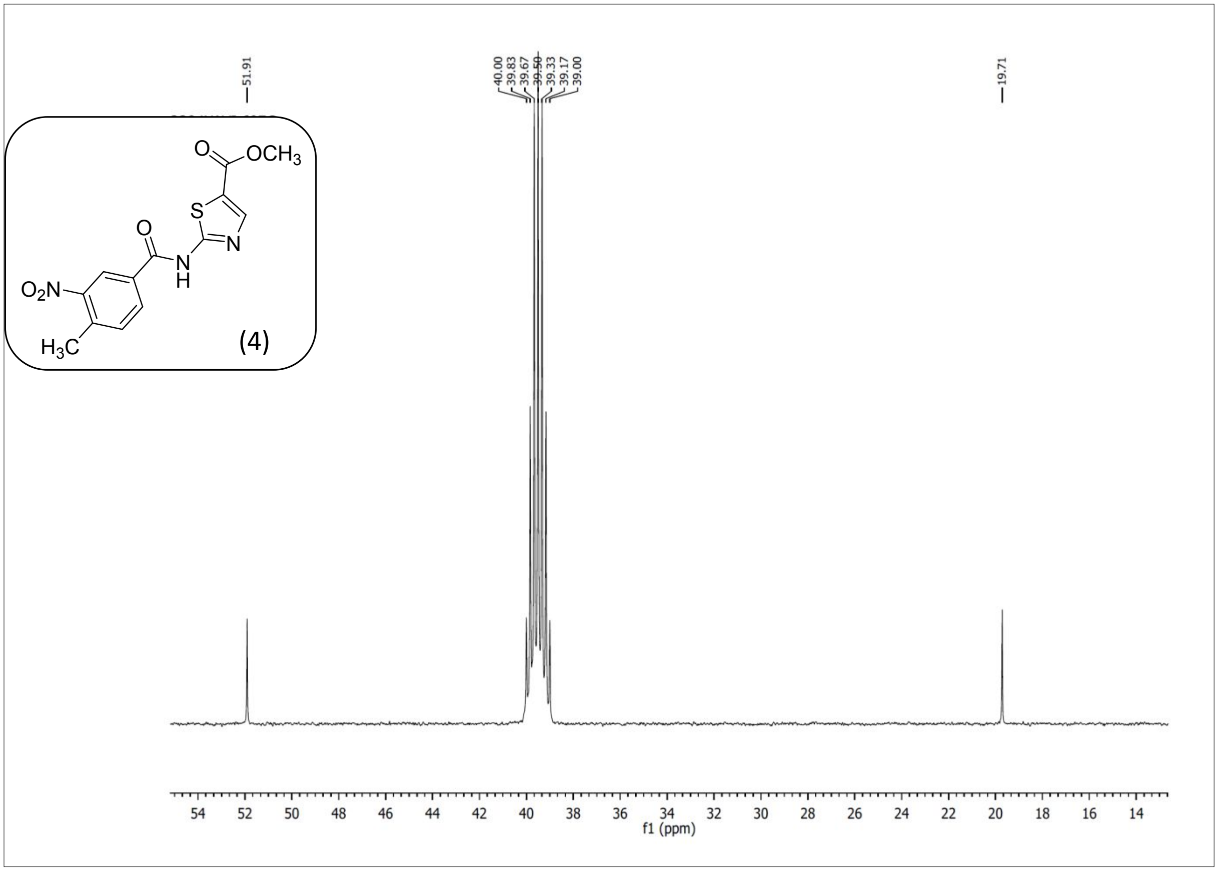

Figure S2. ^{13}C NMR of Molecule (4).

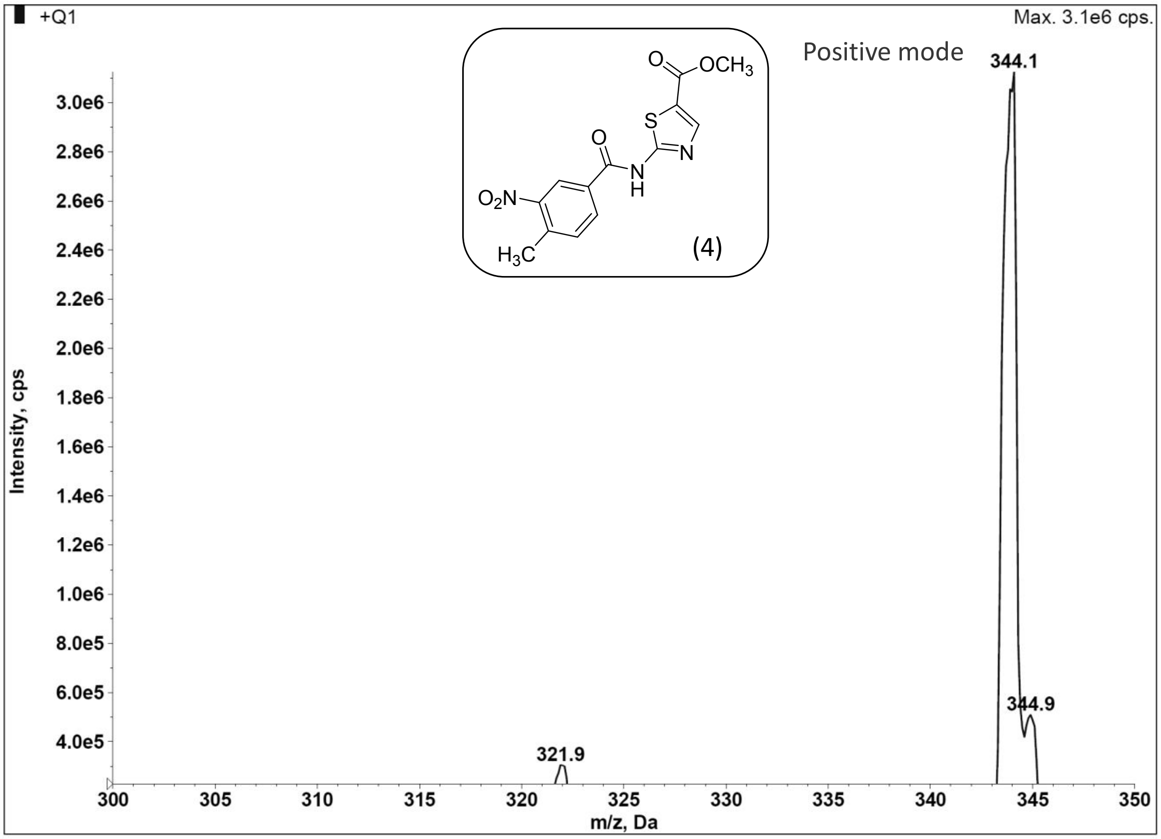

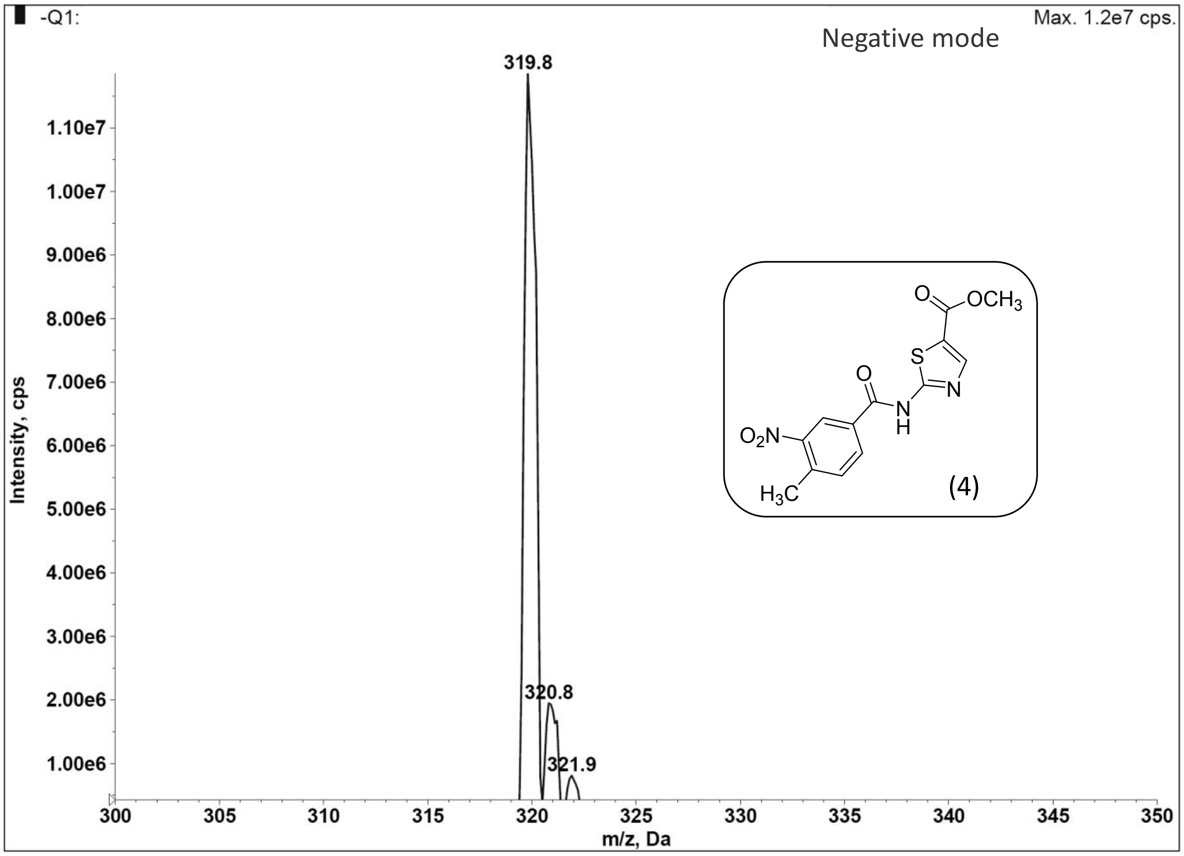

Figure S3. Mass Spectroscopy of Molecule (4).

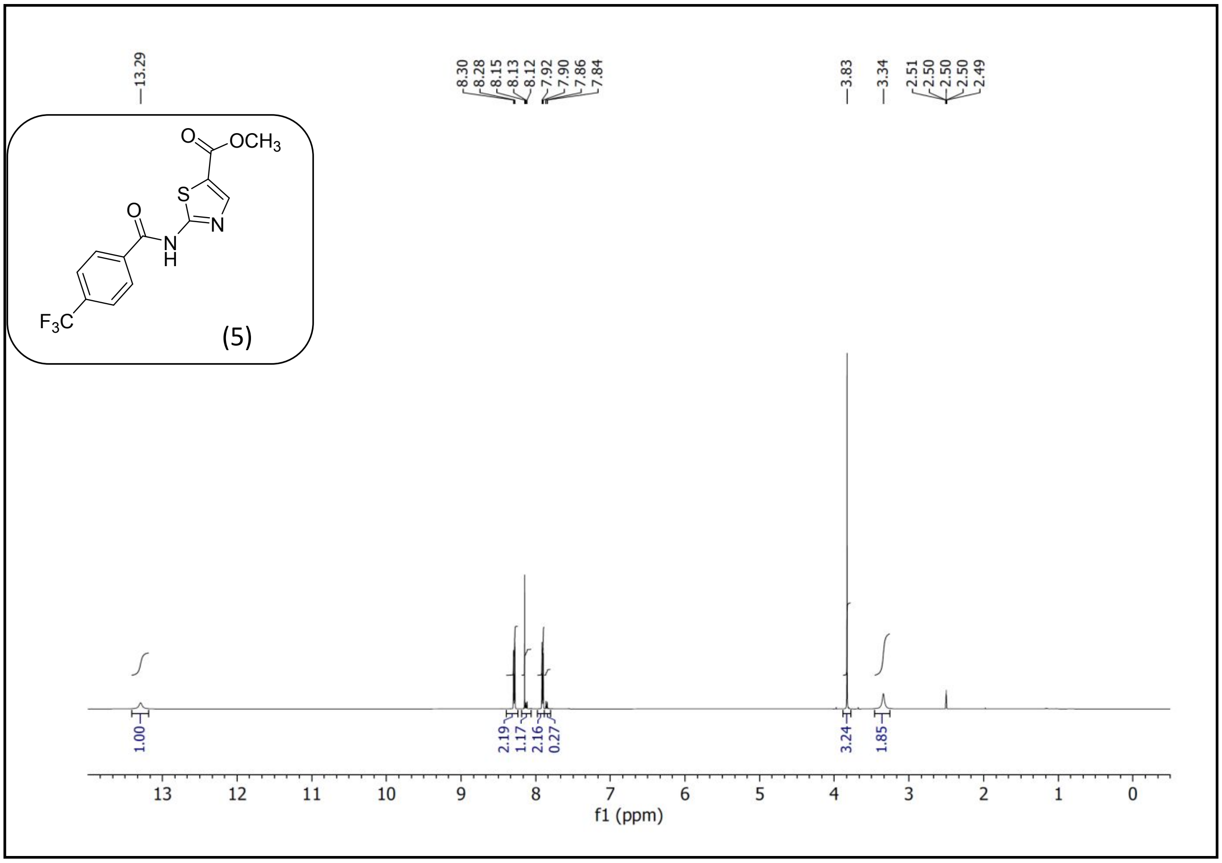
13.29
8.30
8.28
8.15
8.13
8.12
7.92
7.90
7.86
7.84
3.83
3.34
2.51
2.50
2.50
2.50
2.49
O
OCH3
S
N
O
N
H
F3C
(5)
1.00
2.19
1.17
2.16
0.27
3.24
1.85
13
12
11
10
9
8
7
6
5
4
3
2
1
0
f1 (ppm)

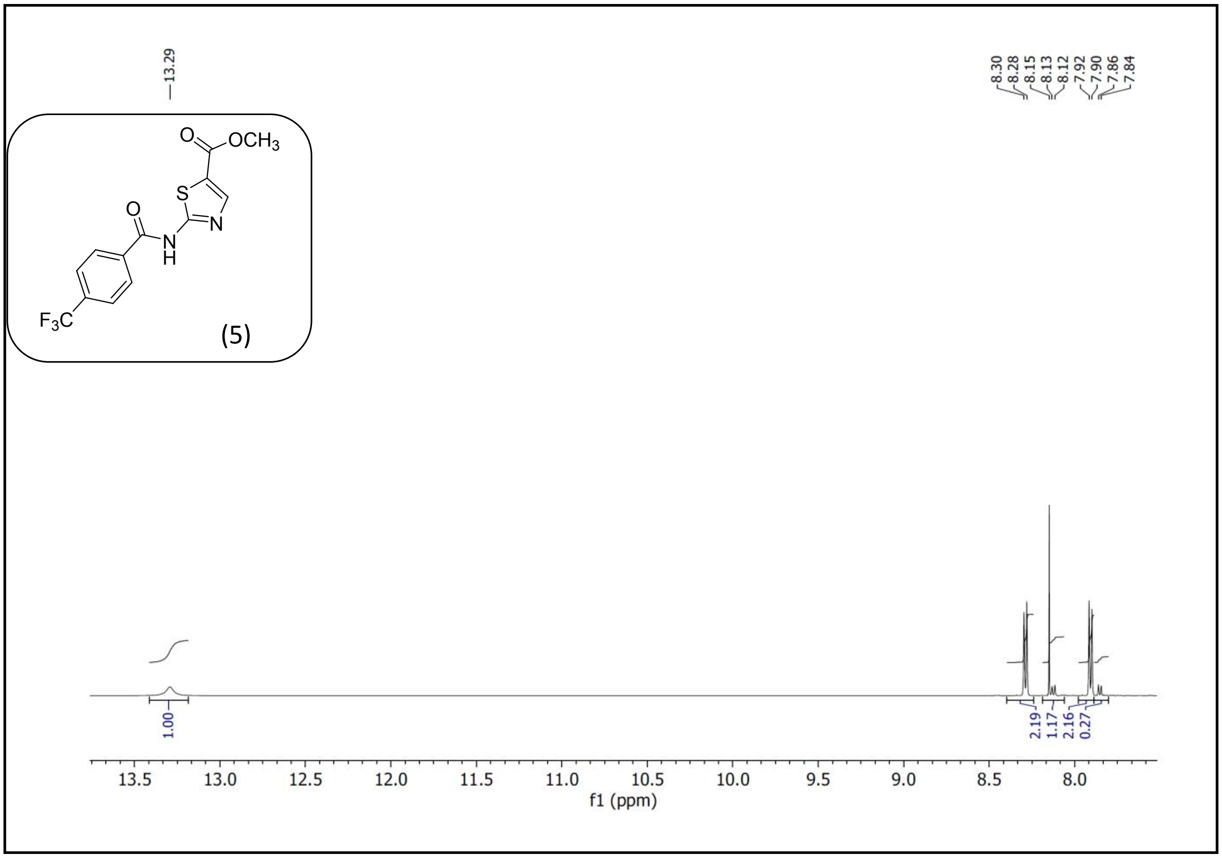
13.29
8.30
8.28
8.15
8.13
8.12
7.92
7.90
7.86
7.84
O
OCH3
S
N
O
N
H
F3C
(5)
1.00
2.19
1.17
2.16
0.27
13.5
13.0
12.5
12.0
11.5
11.0
10.5
10.0
9.5
9.0
8.5
8.0
f1 (ppm)

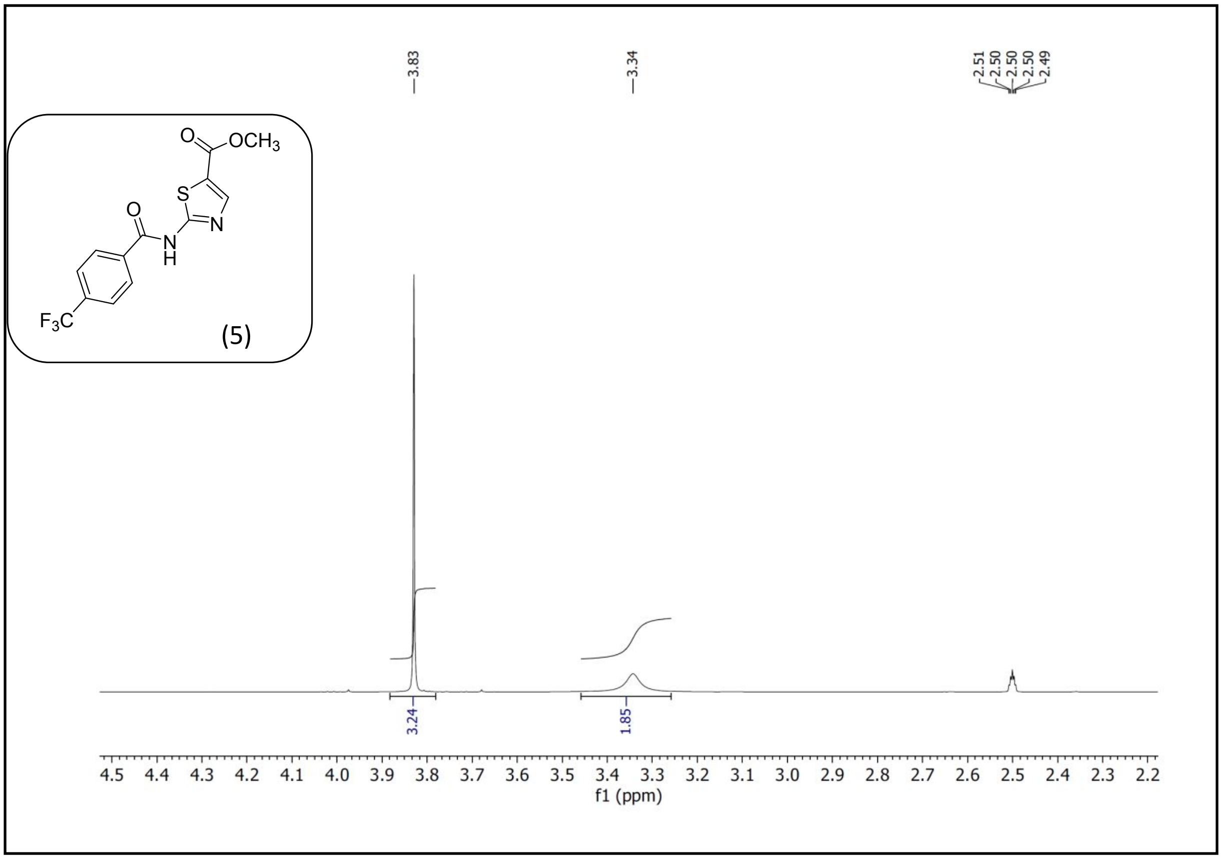

Figure S4. ^{1}H NMR of Molecule (5).

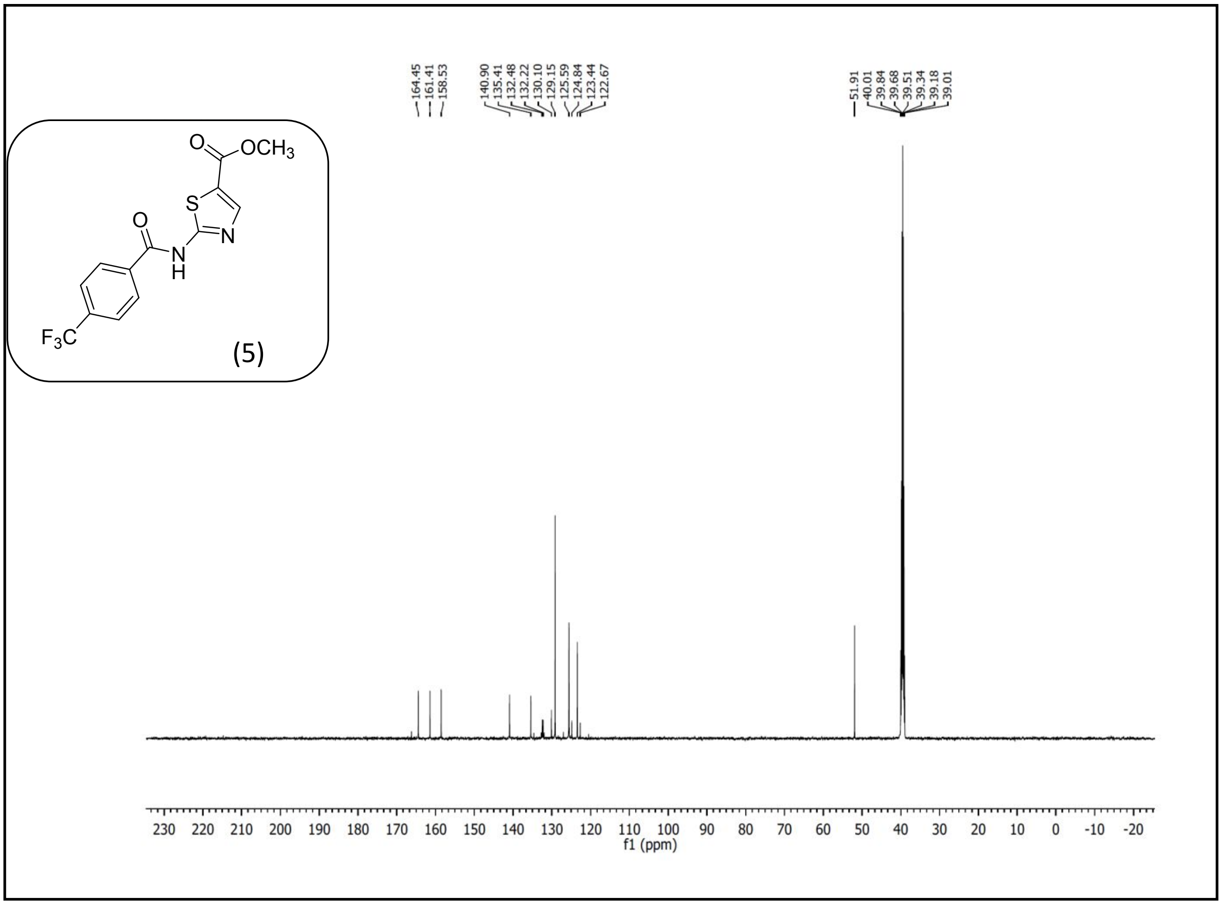
164.45
161.41
158.53
140.90
135.41
132.48
132.22
130.10
129.15
125.59
124.84
123.44
122.67
51.91
40.01
39.84
39.68
39.51
39.34
39.18
39.01
OCH3
O
S
N
O
N
H
F3C
(5)
230 220 210 200 190 180 170 160 150 140 130 120 110 100 90 80 70 60 50 40 30 20 10 0 -10 -20
f1 (ppm)

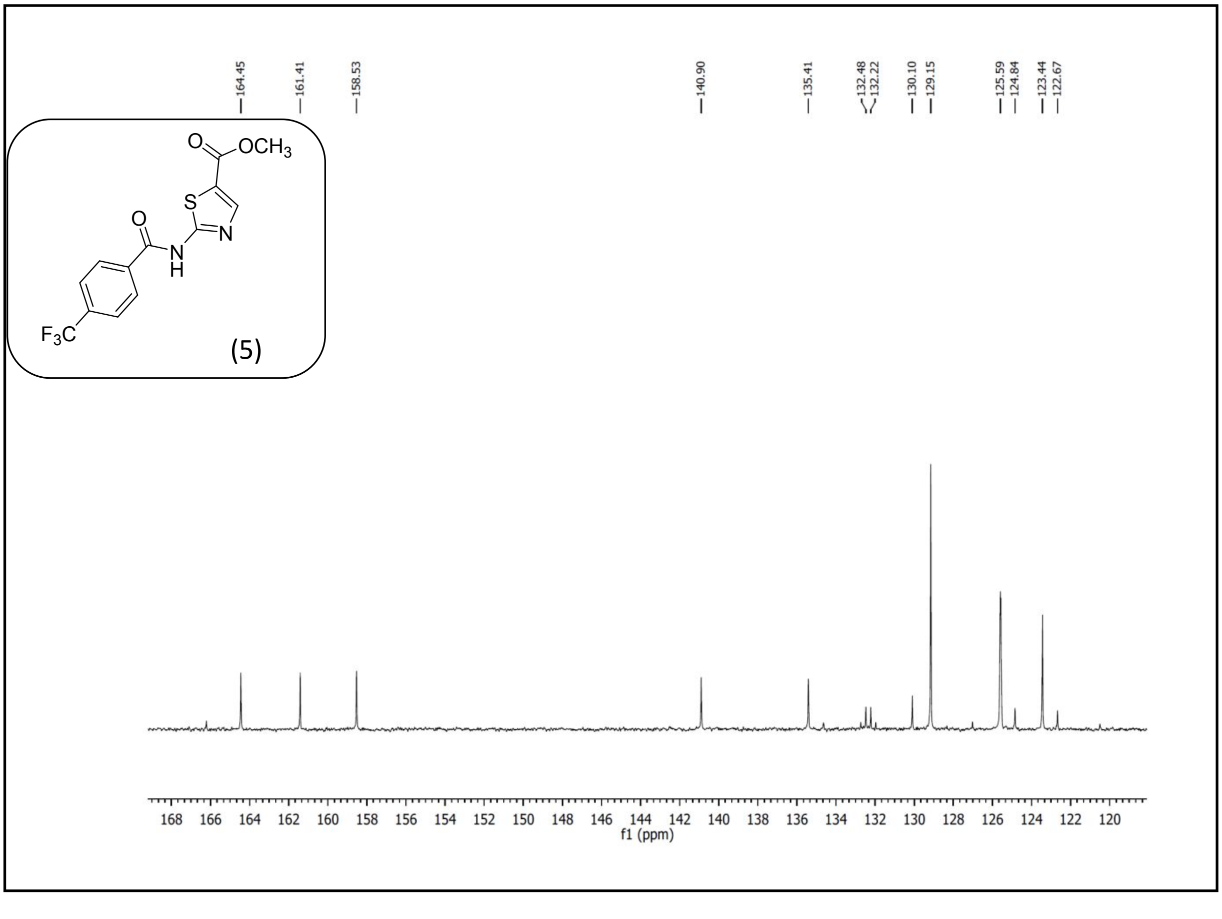
164.45
161.41
158.53
140.90
135.41
132.48
132.22
130.10
129.15
125.59
124.84
123.44
122.67
OCH3
O
S
N
O
N
H
F3C
(5)
168 166 164 162 160 158 156 154 152 150 148 146 144 142 140 138 136 134 132 130 128 126 124 122 120
f1 (ppm)

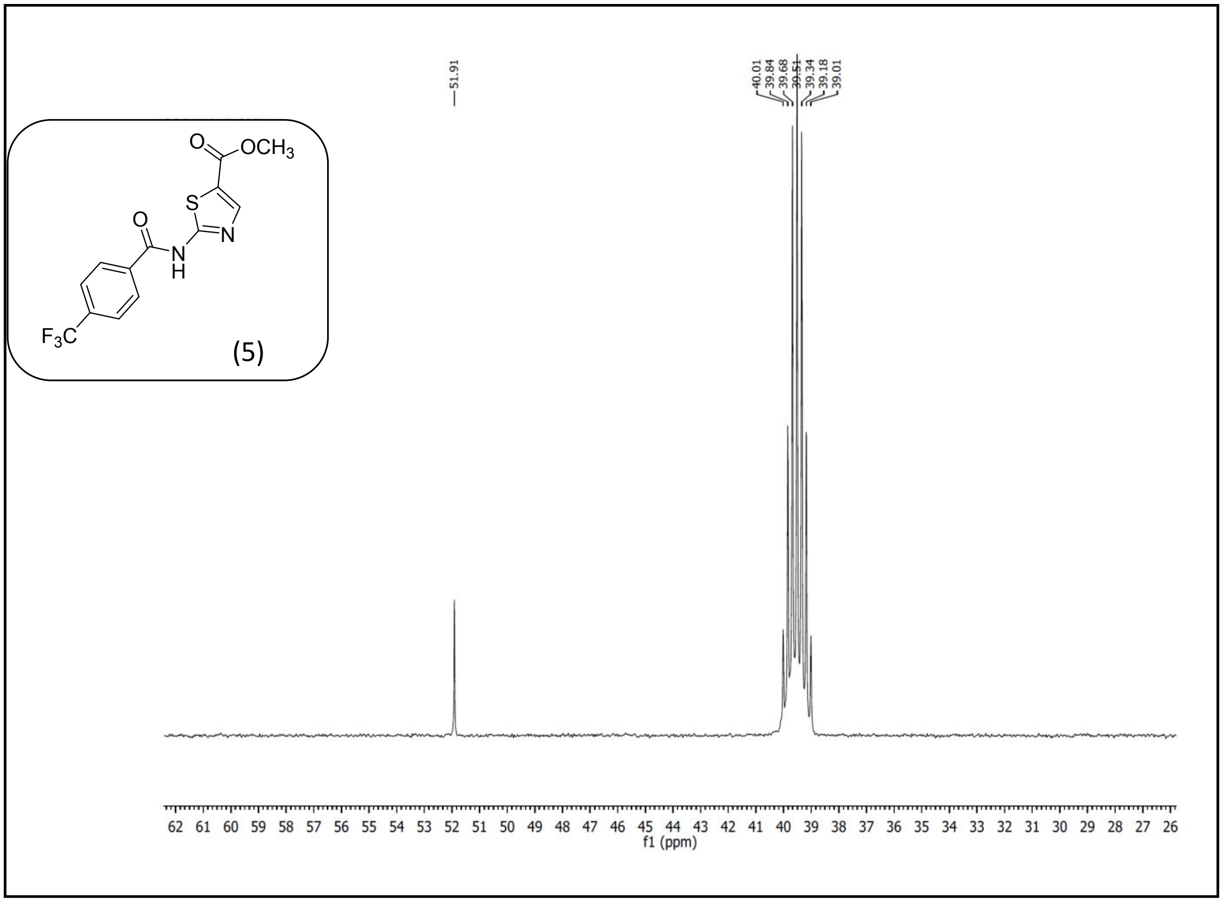

Figure S5. ^{13}C NMR of Molecule (5).

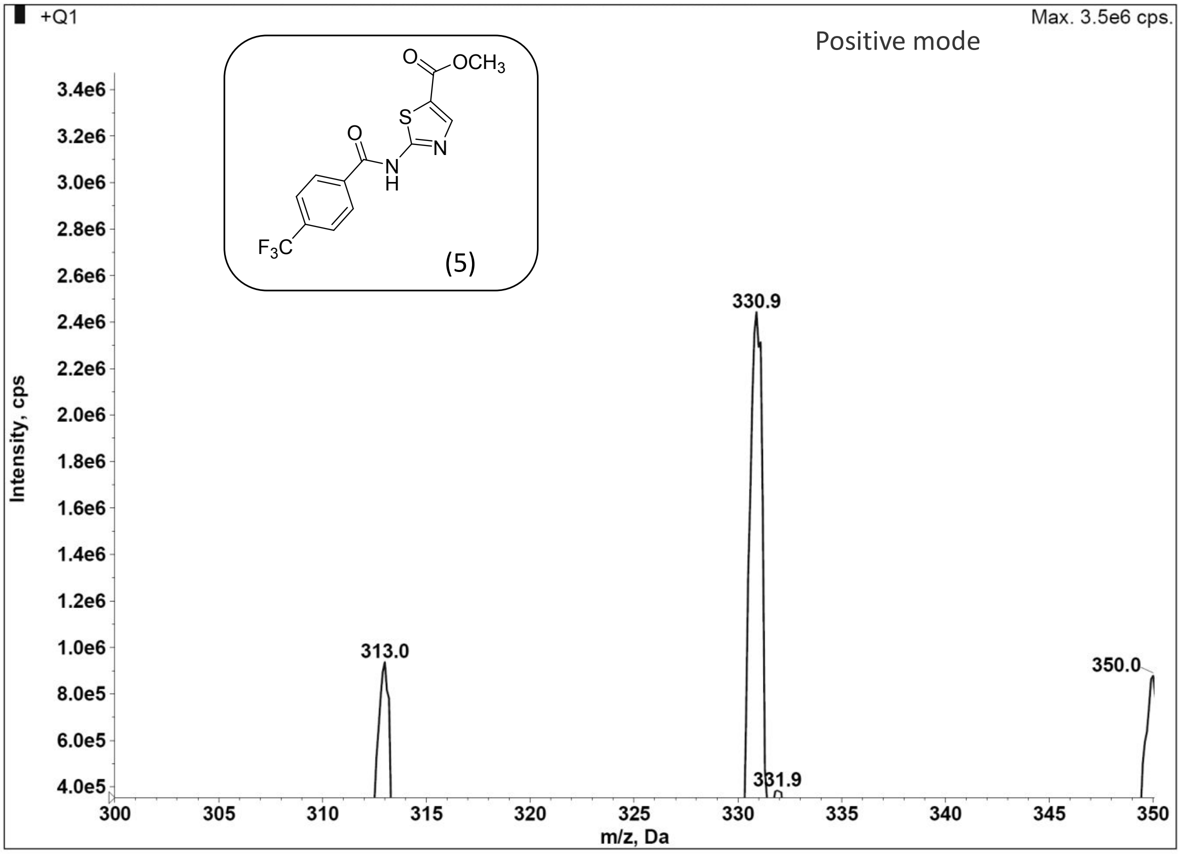

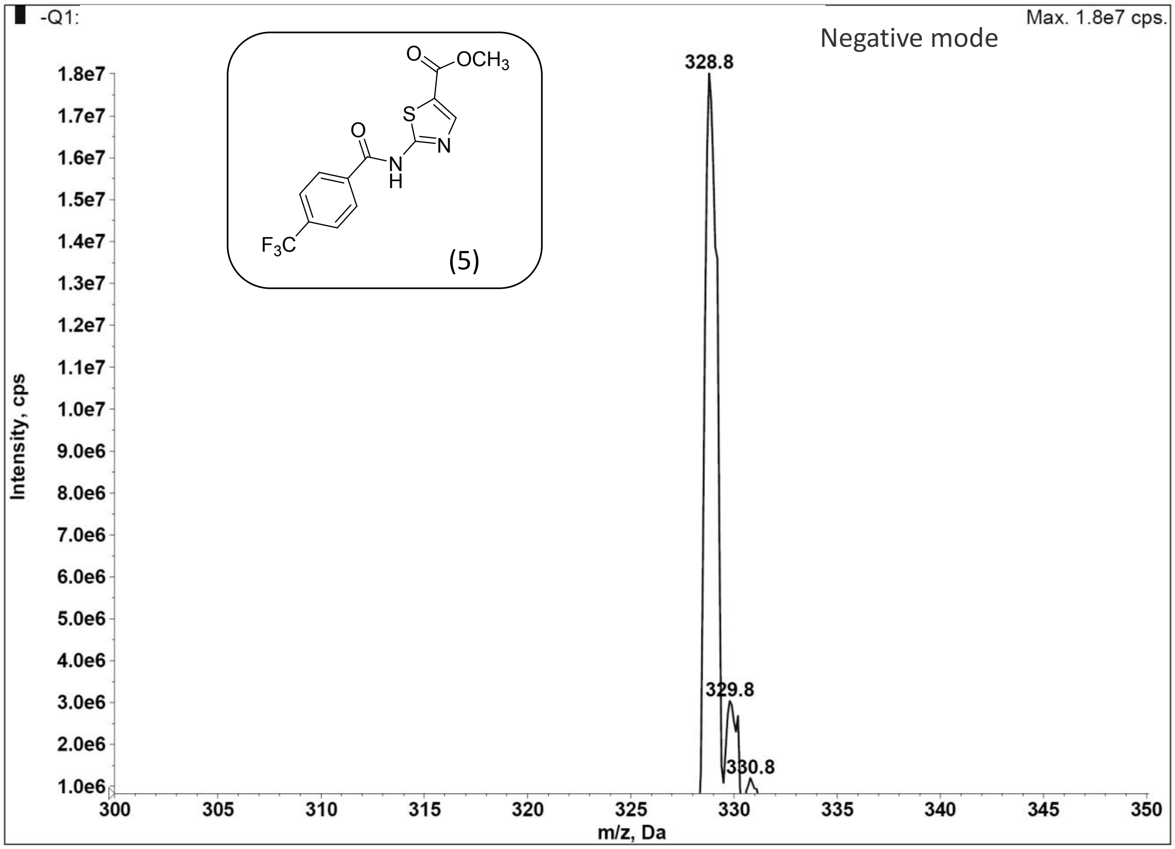

Figure S6. Mass Spectroscopy of Molecule (5).

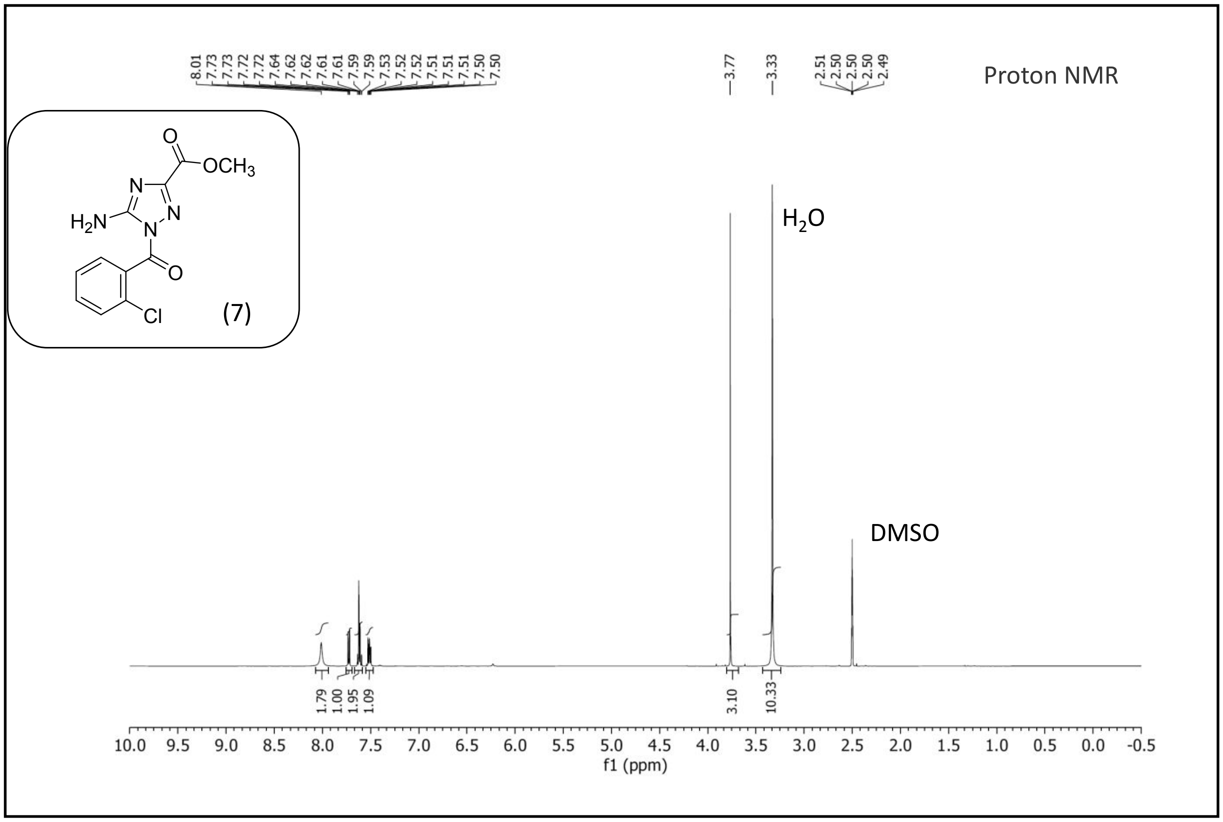
Proton NMR
H_2O
DMSO
O
OCH_3
N
N
N
H_2N
O
Cl
(7)
8.01 7.73 7.73 7.72 7.72 7.64 7.62 7.61 7.61 7.59 7.59 7.53 7.52 7.52 7.51 7.51 7.50 7.50
3.77
3.33
2.51 2.50 2.50 2.50 2.49
1.79 1.00 1.95 1.09
3.10
10.33
10.0 9.5 9.0 8.5 8.0 7.5 7.0 6.5 6.0 5.5 5.0 4.5 4.0 3.5 3.0 2.5 2.0 1.5 1.0 0.5 0.0 -0.5
f1 (ppm)

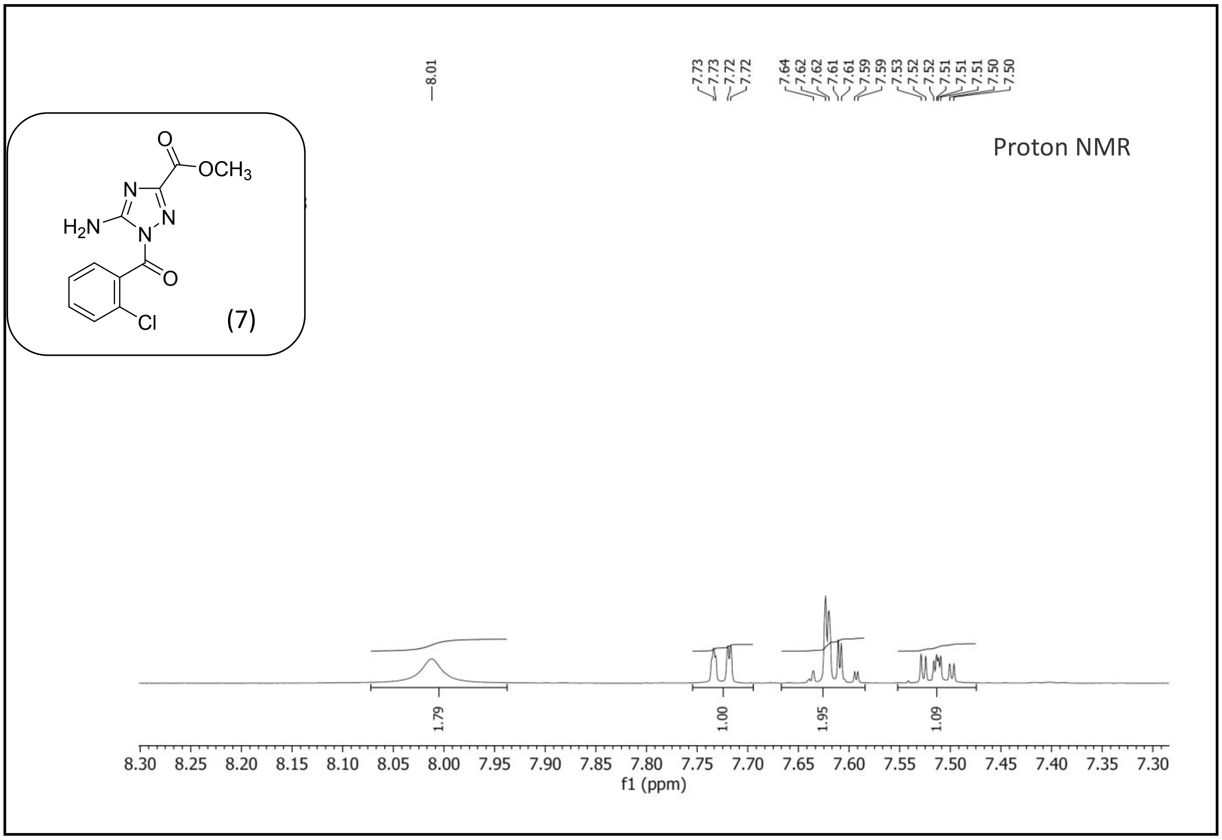
Proton NMR
O
OCH_3
N
N
N
H_2N
O
Cl
(7)
8.01
7.73 7.73 7.72 7.72
7.64 7.62 7.62 7.61 7.61 7.59 7.59 7.53 7.52 7.52 7.51 7.51 7.50 7.50
1.79
1.00
1.95
1.09
8.30 8.25 8.20 8.15 8.10 8.05 8.00 7.95 7.90 7.85 7.80 7.75 7.70 7.65 7.60 7.55 7.50 7.45 7.40 7.35 7.30
f1 (ppm)

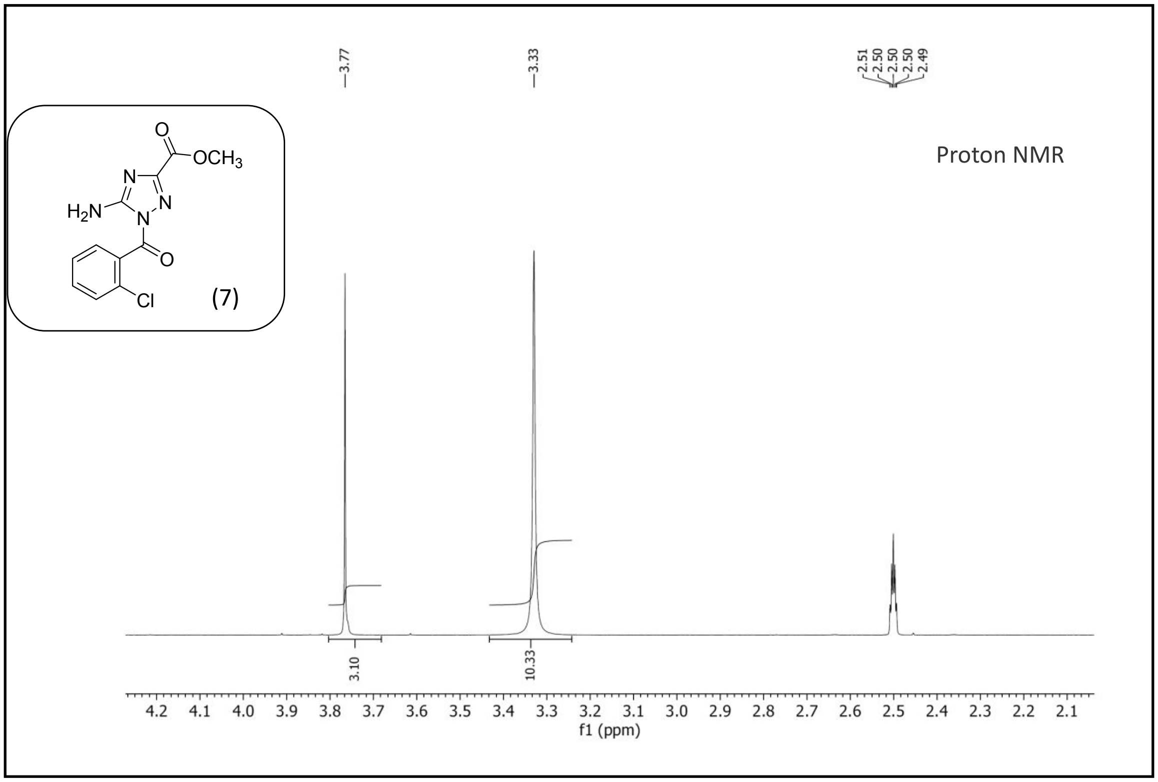

Figure S7. ^{1}H NMR of Molecule (7).

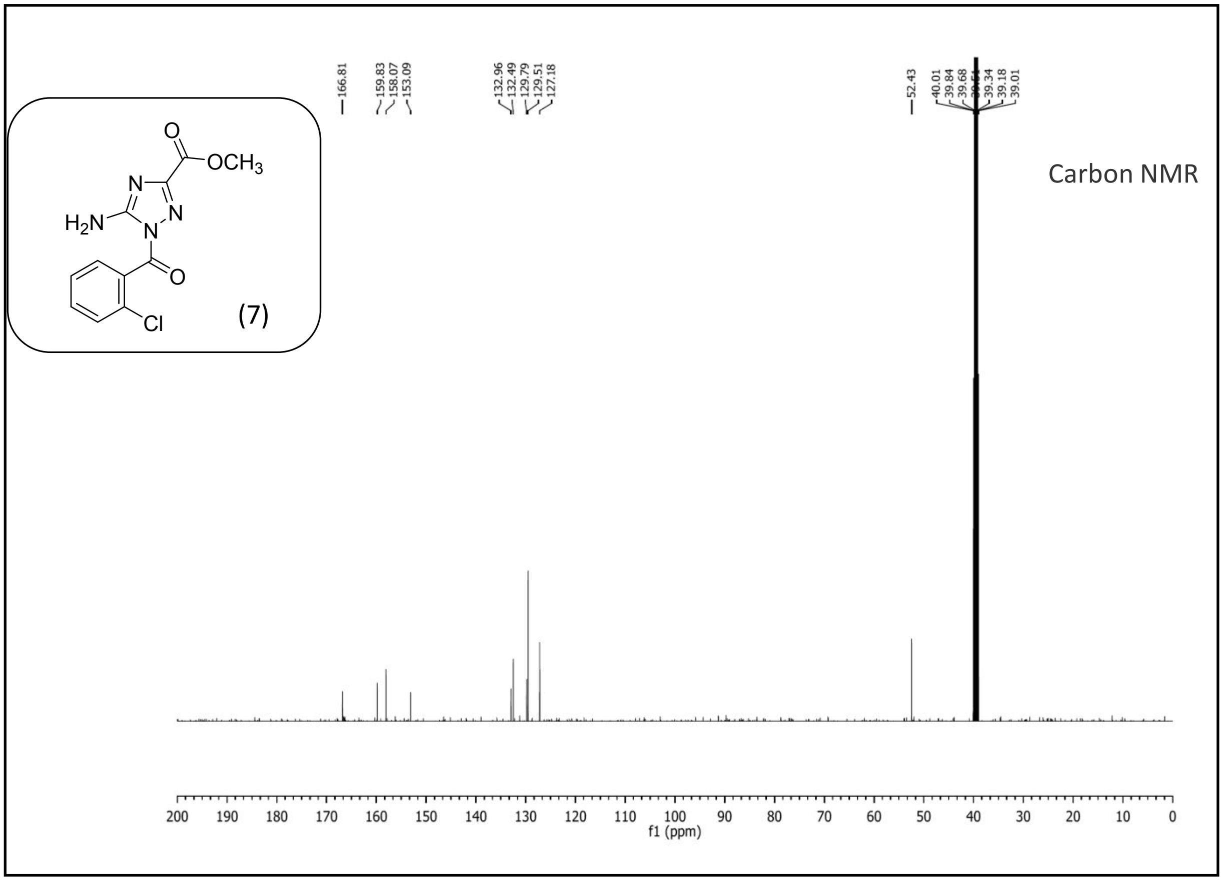
Carbon NMR
(7)
f1 (ppm)

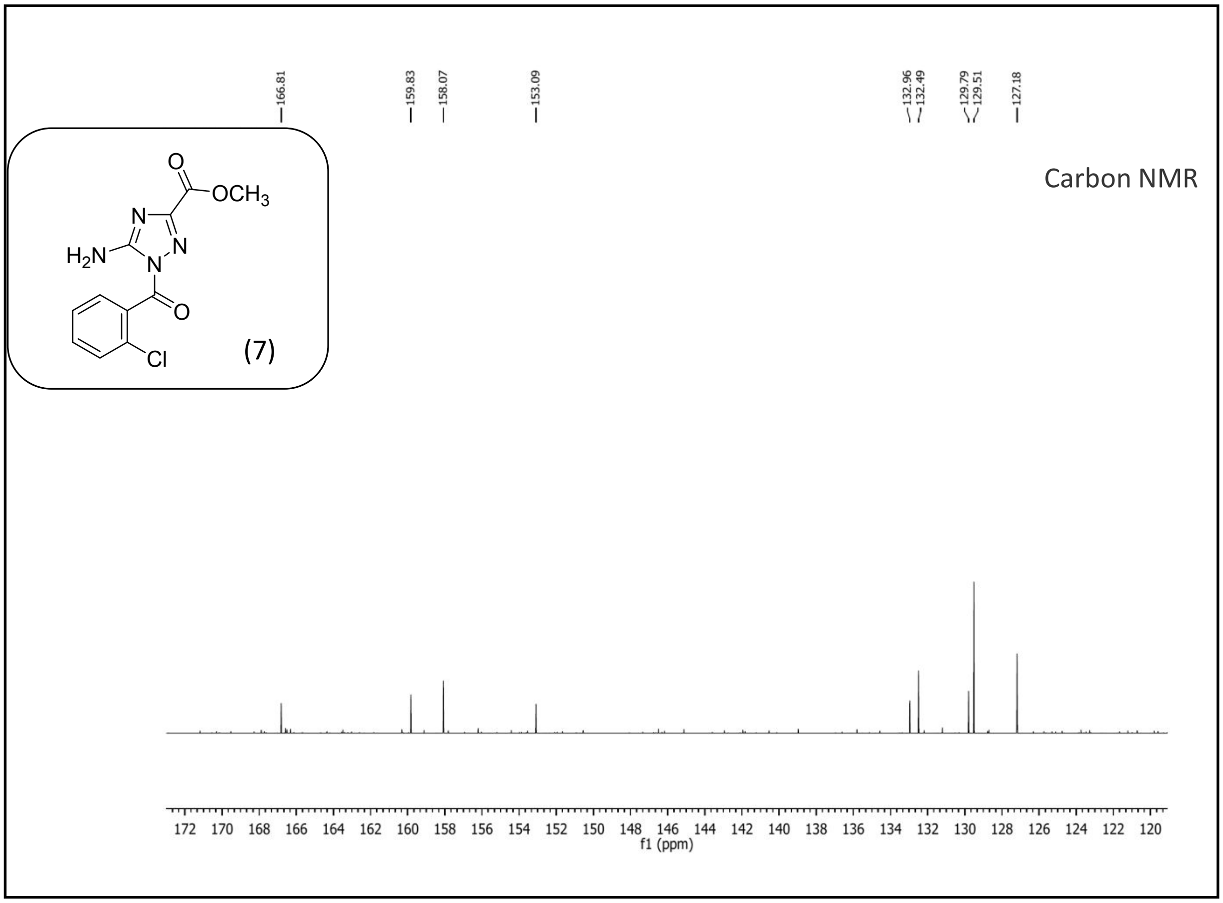
Carbon NMR
(7)
f1 (ppm)

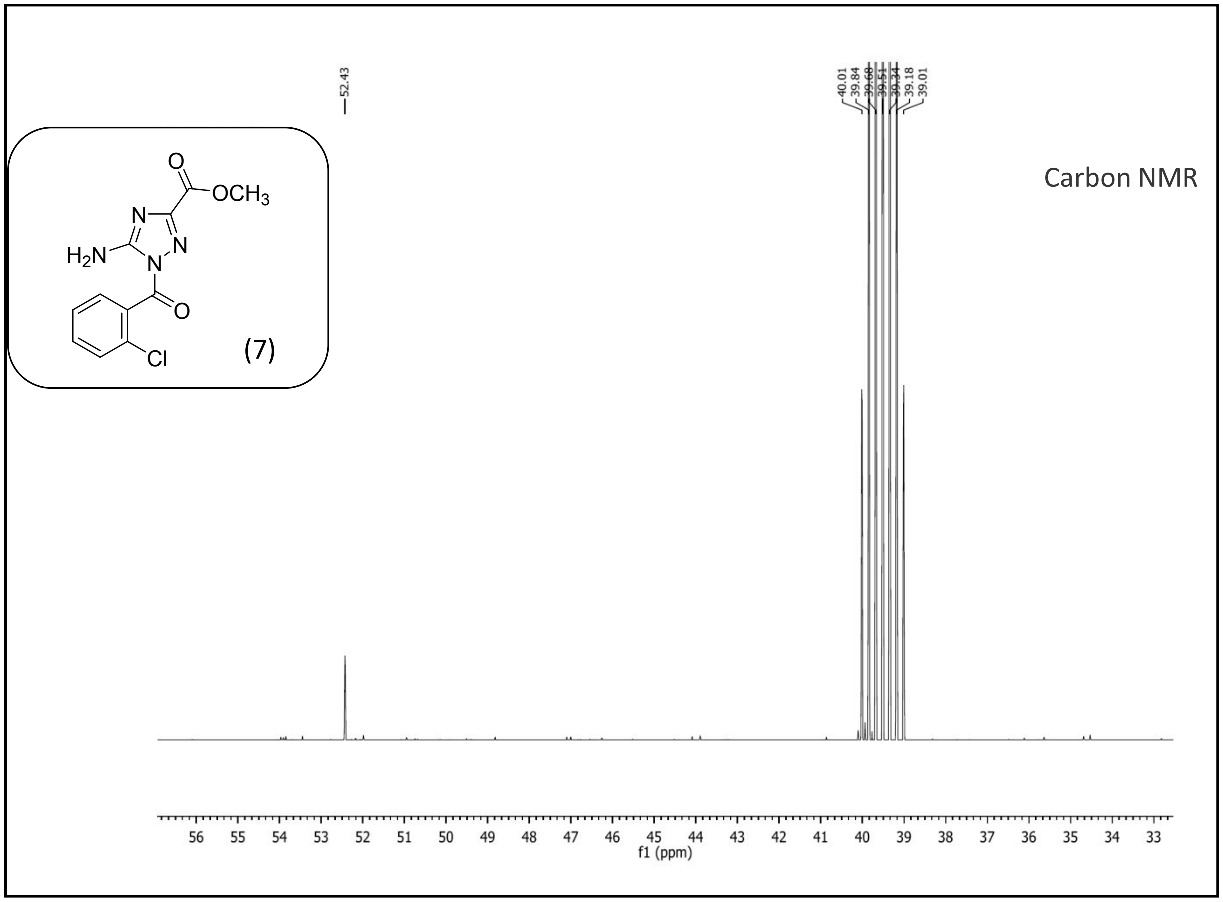

Figure S8. ^{13}C NMR of Molecule (7).

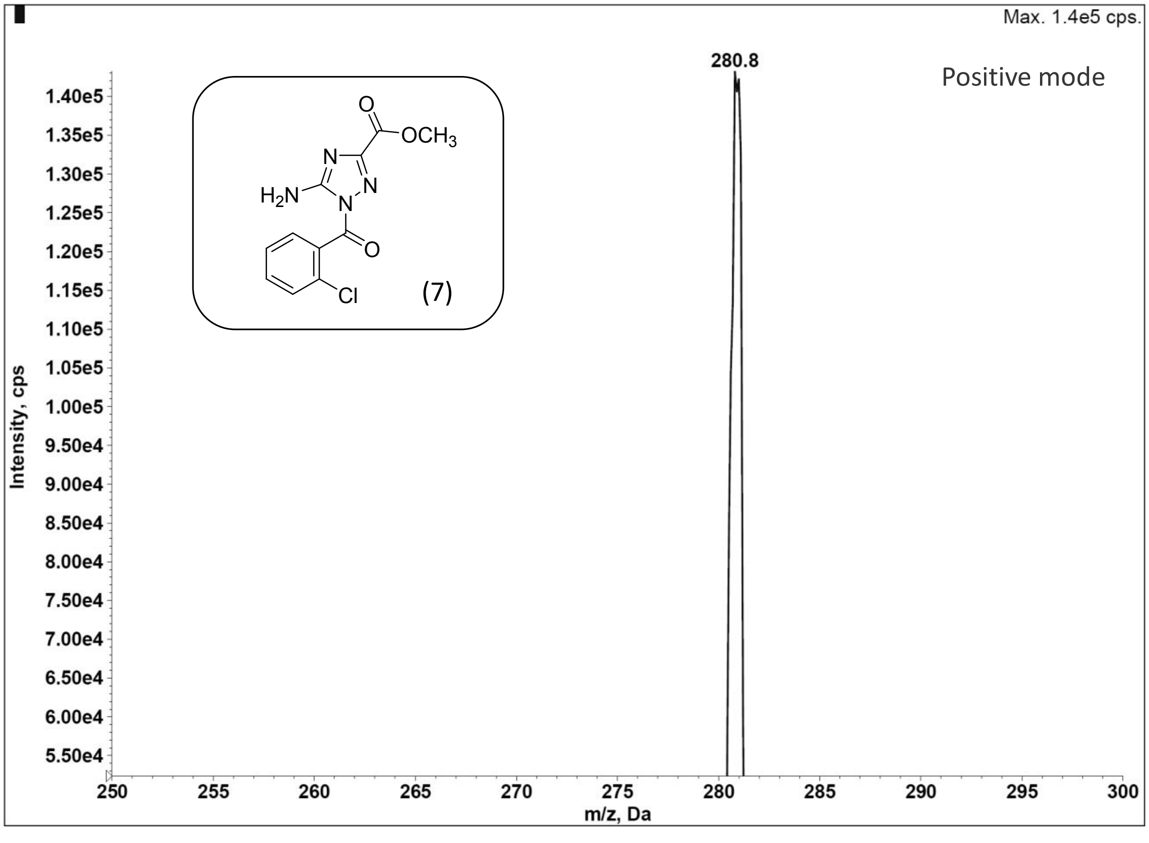

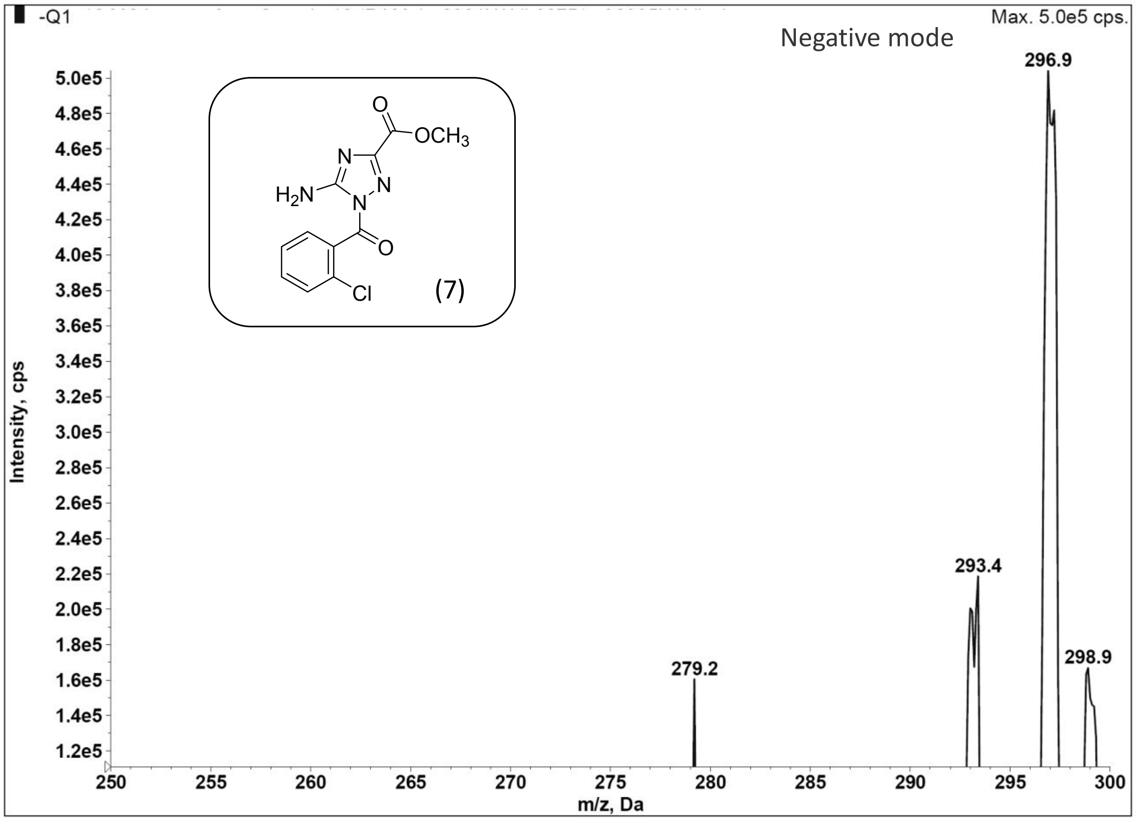

Figure S9. Mass Spectroscopy of Molecule (7).

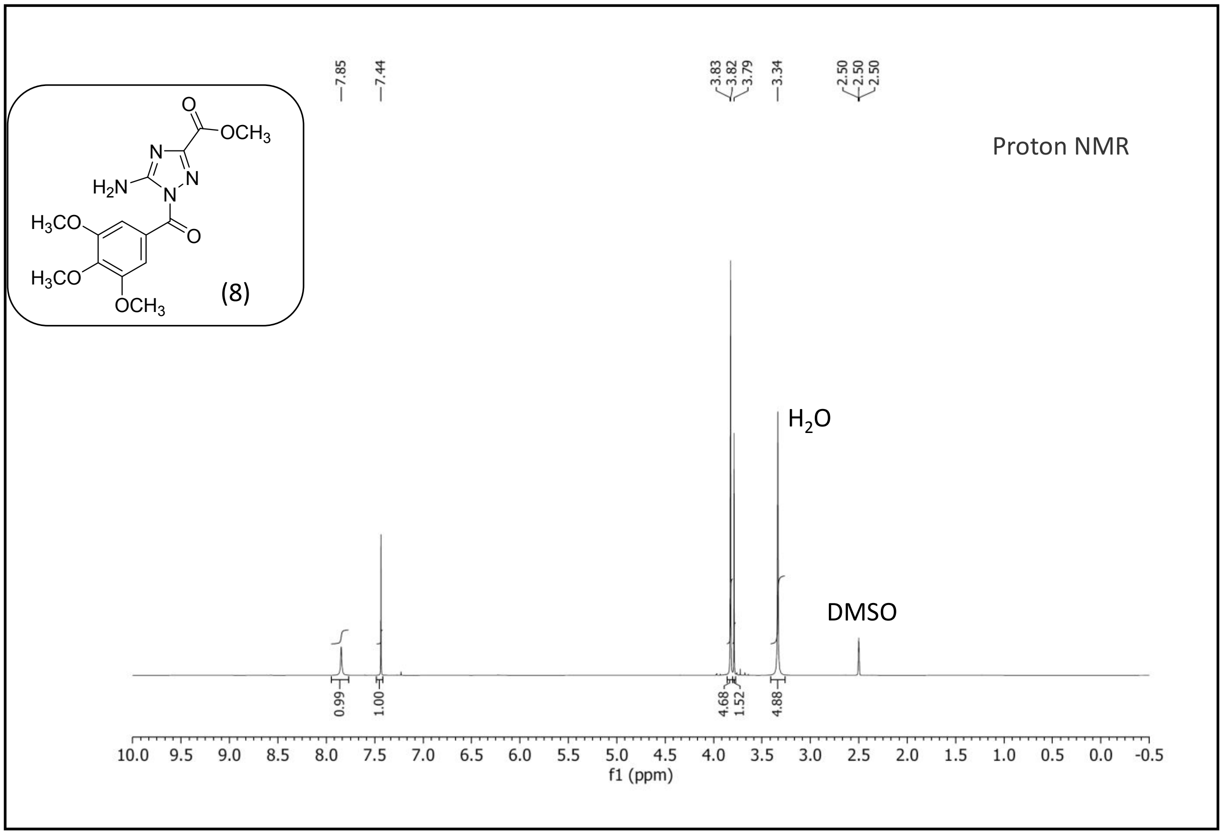

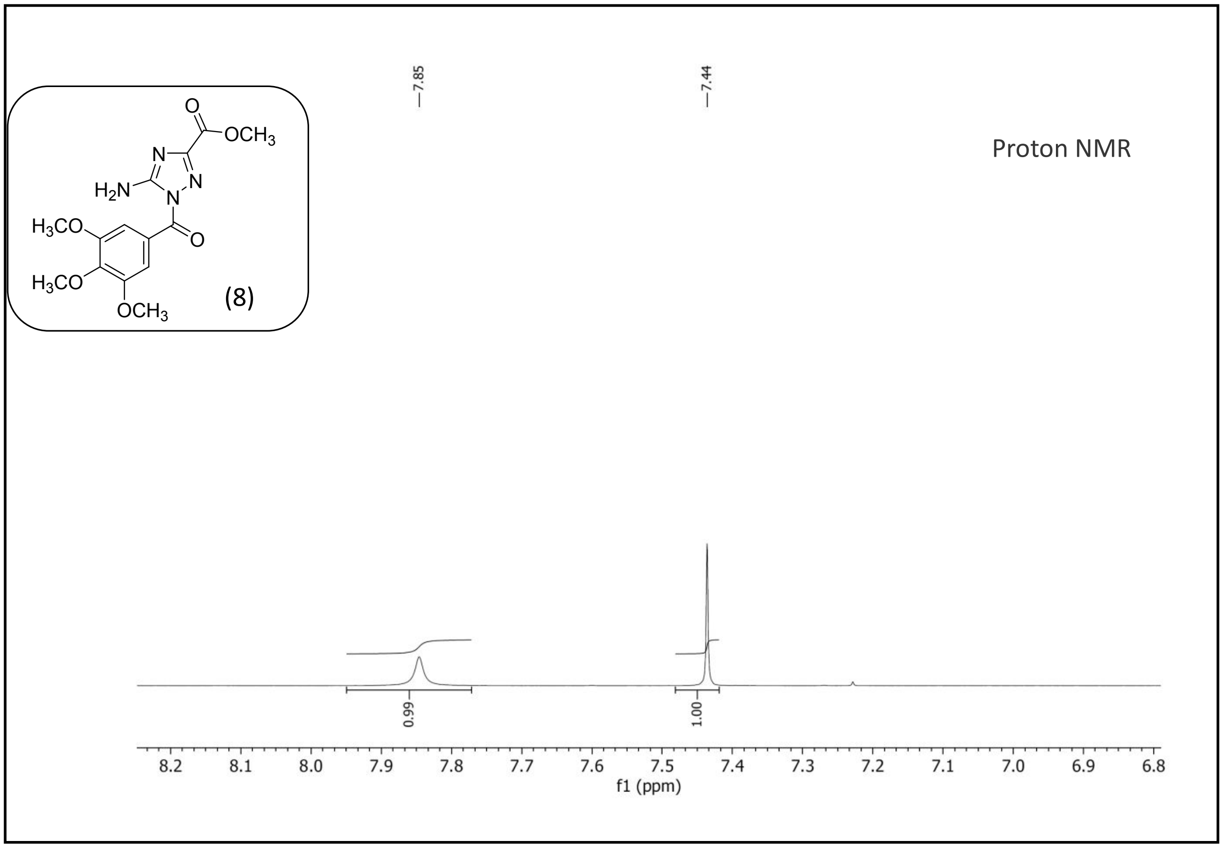

Proton NMR
(8)
7.85
7.44
0.99
1.00
8.2
8.1
8.0
7.9
7.8
7.7
7.6
7.5
7.4
7.3
7.2
7.1
7.0
6.9
6.8
f1 (ppm)

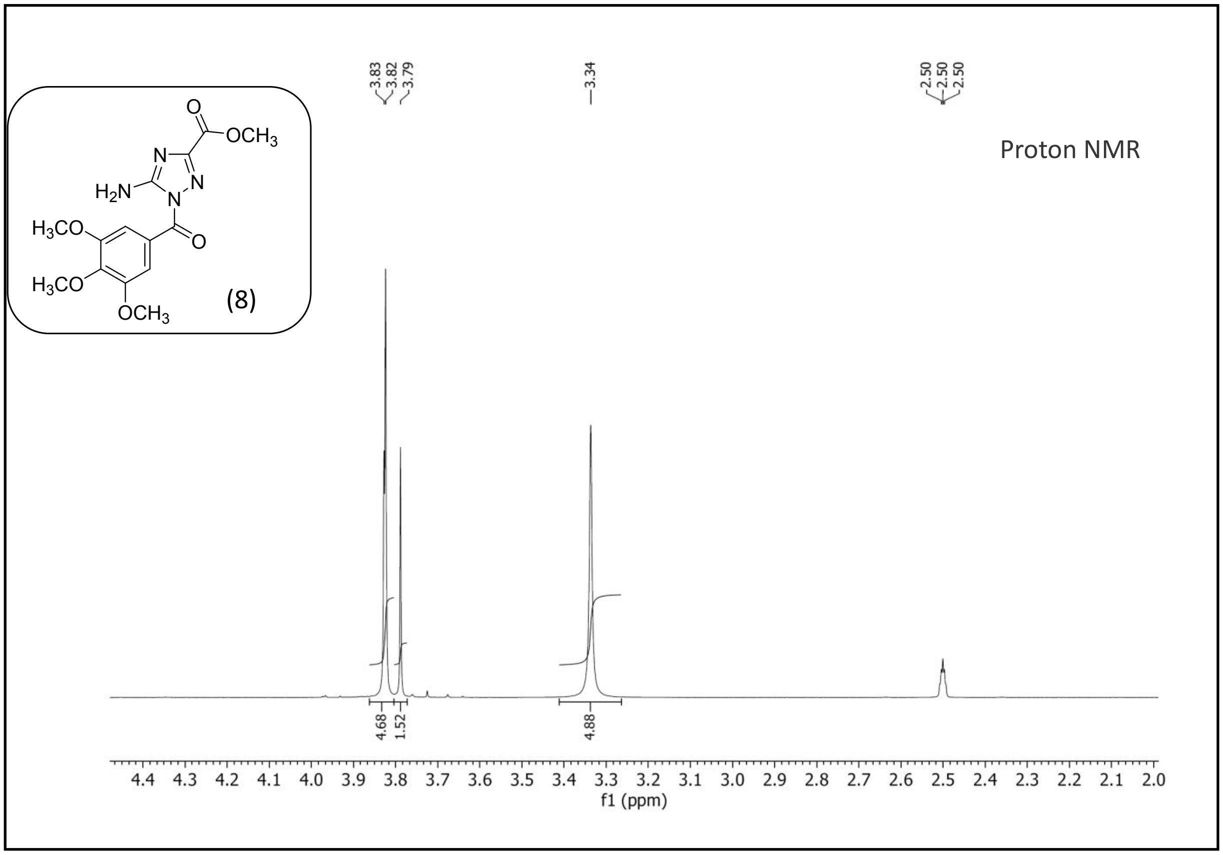

Figure S10. ^{1}H NMR of Molecule (8).

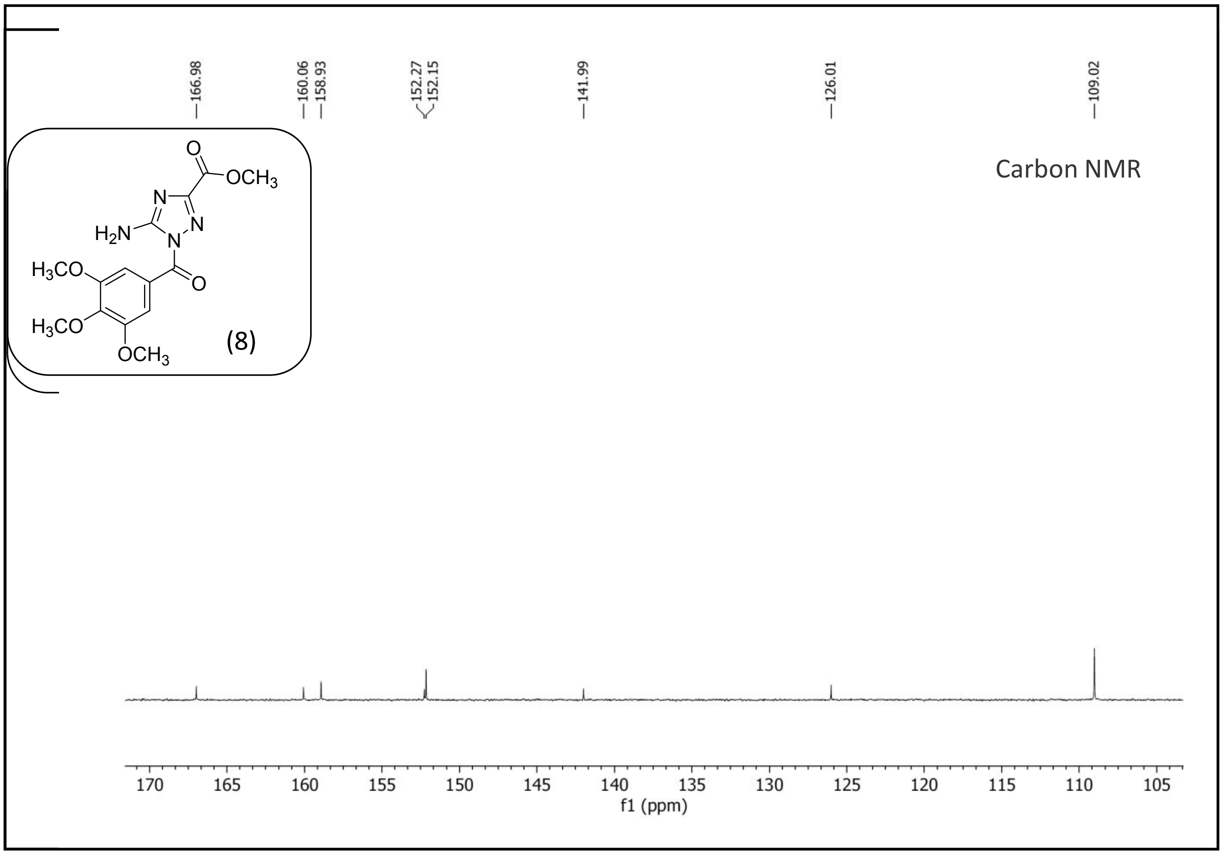

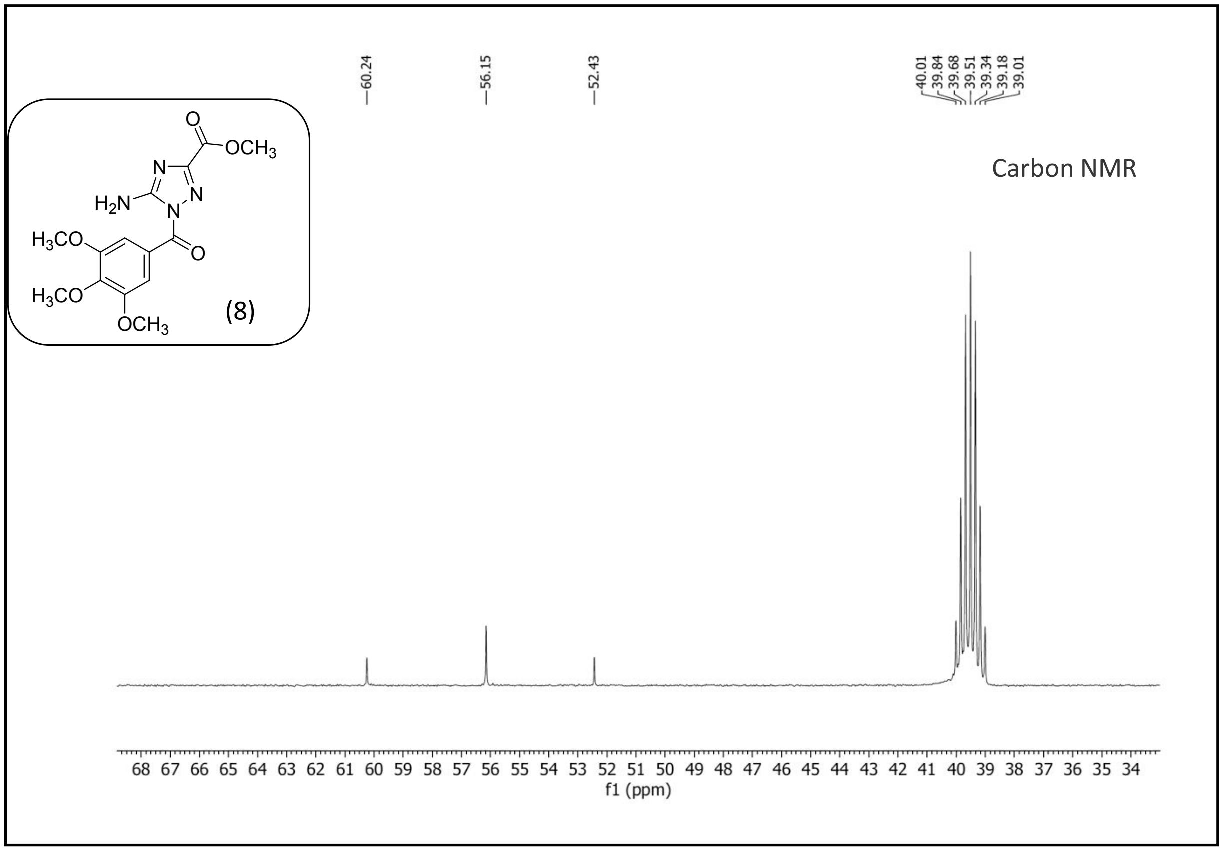

Figure S11. ^{13}C NMR of Molecule (8).

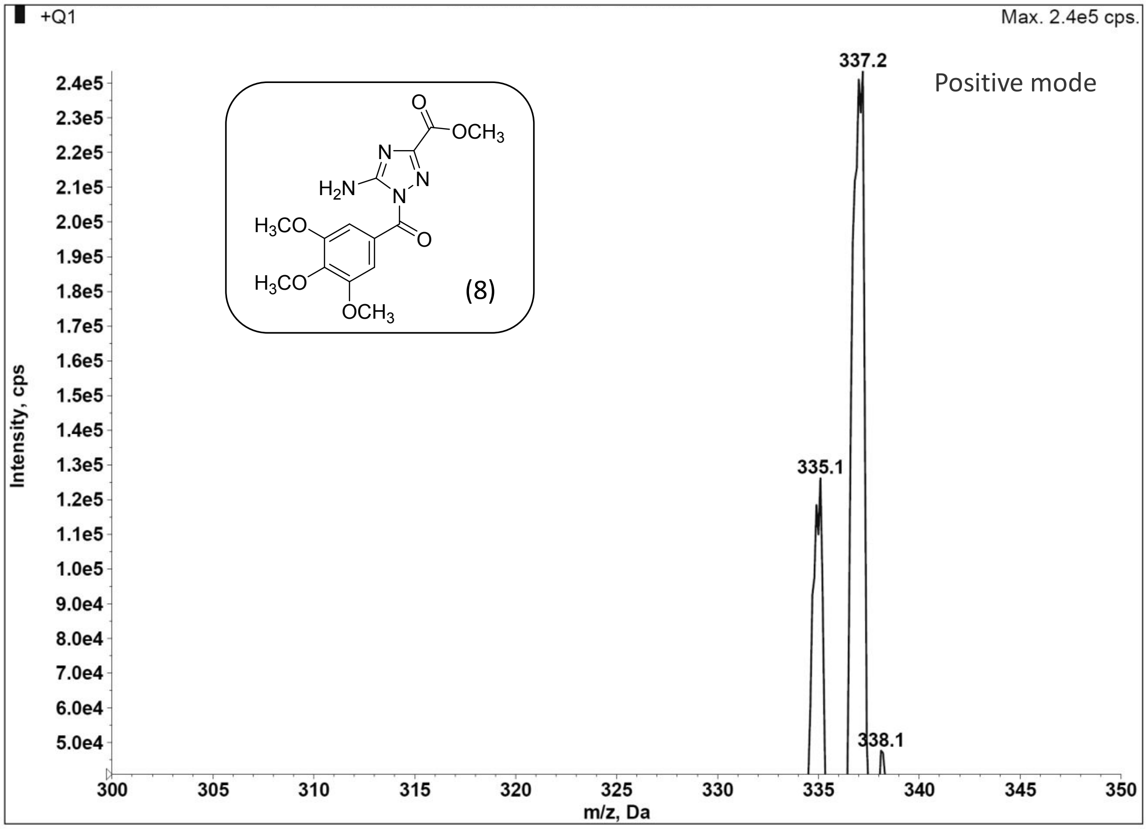

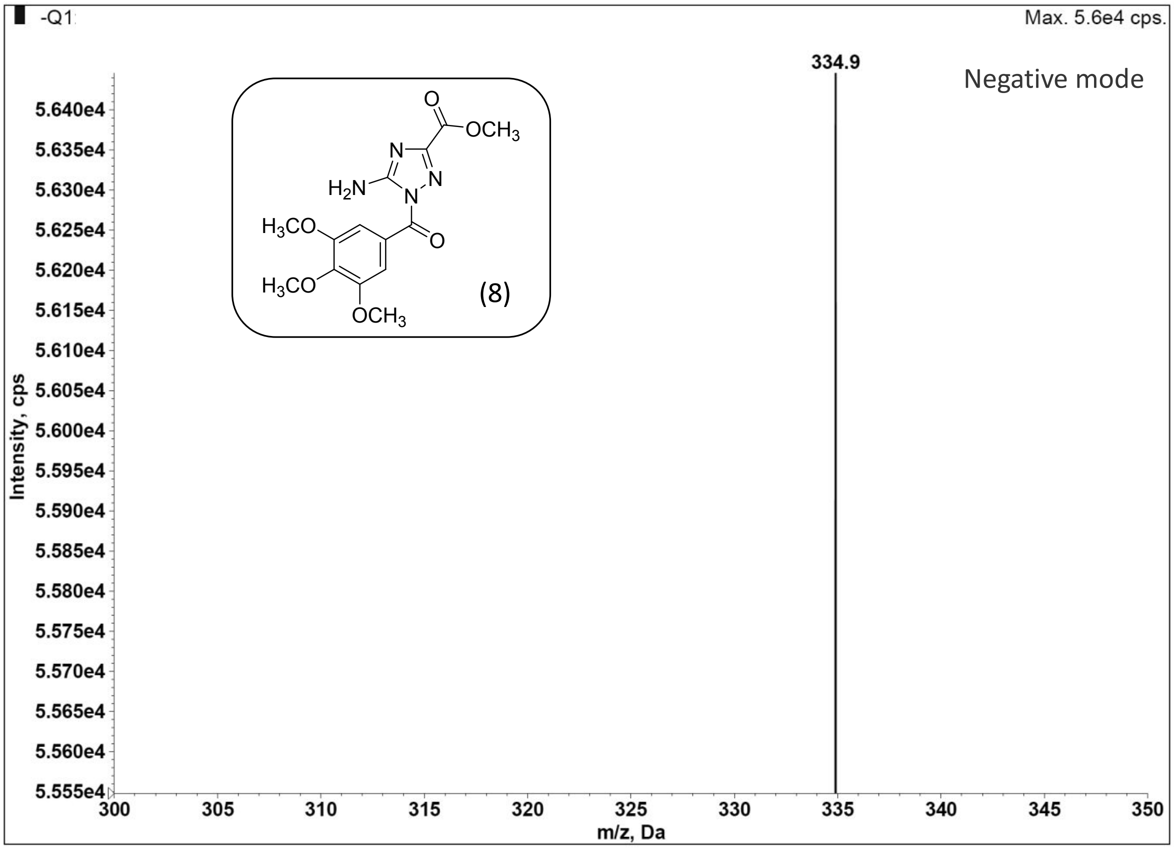

Figure S12. Mass Spectroscopy of Molecule (8).

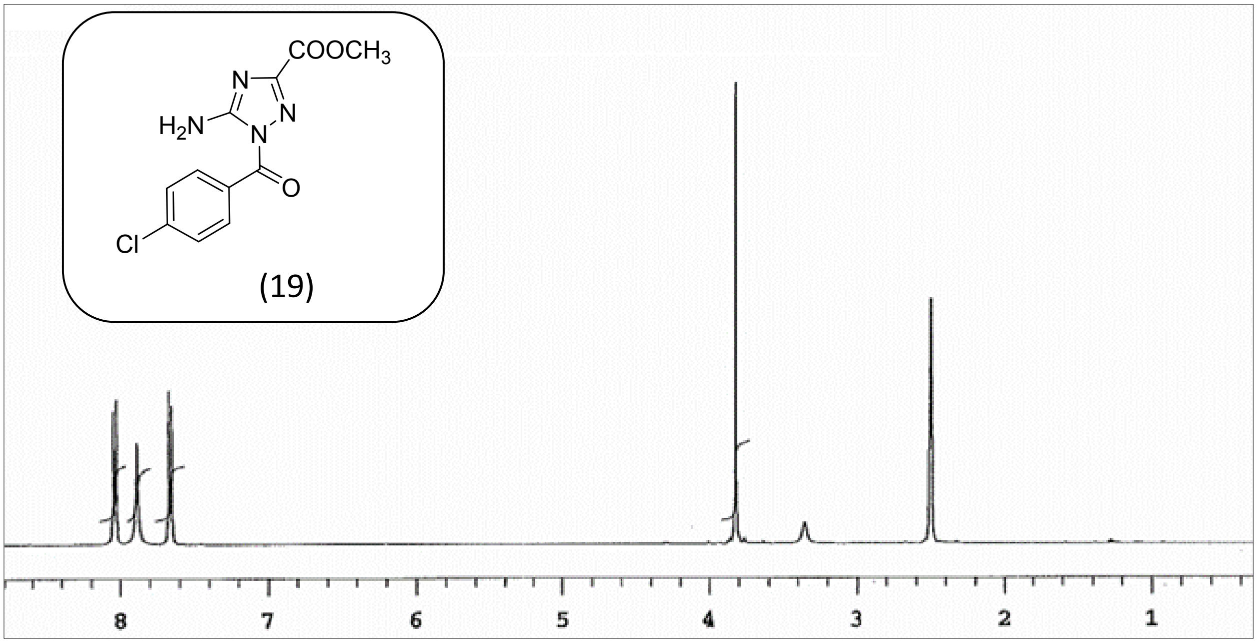

Figure S13. ^{1}H NMR of Molecule (19).

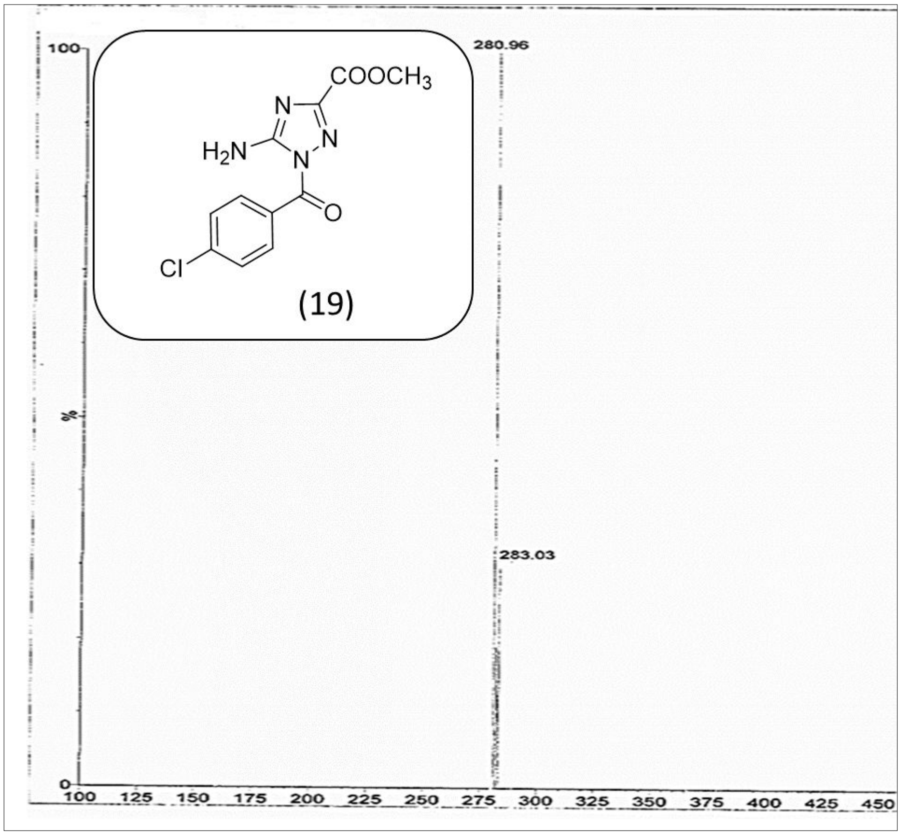

Figure S14. Mass Spectroscopy of Molecule (19).

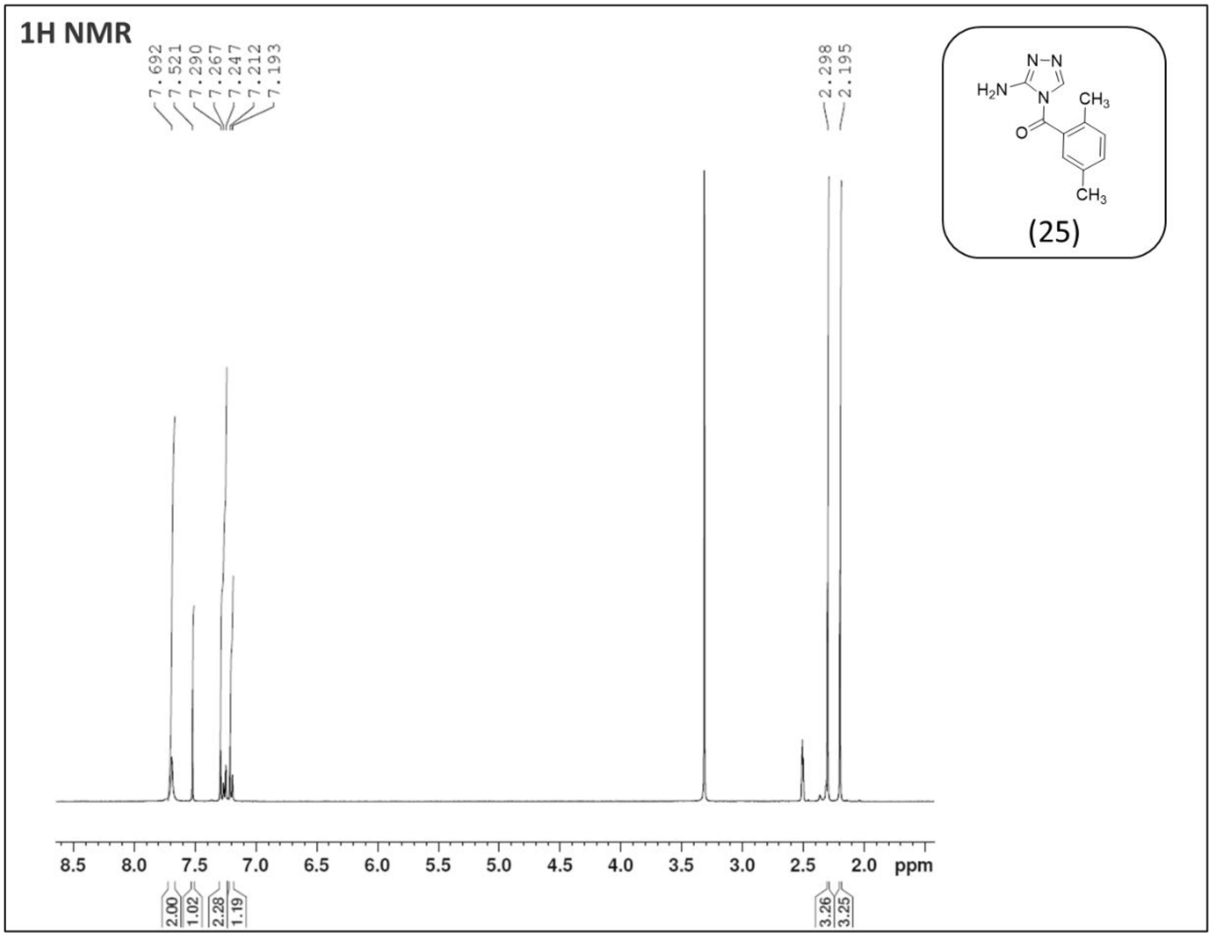

Figure S15. ^{1}H NMR of Molecule (25).

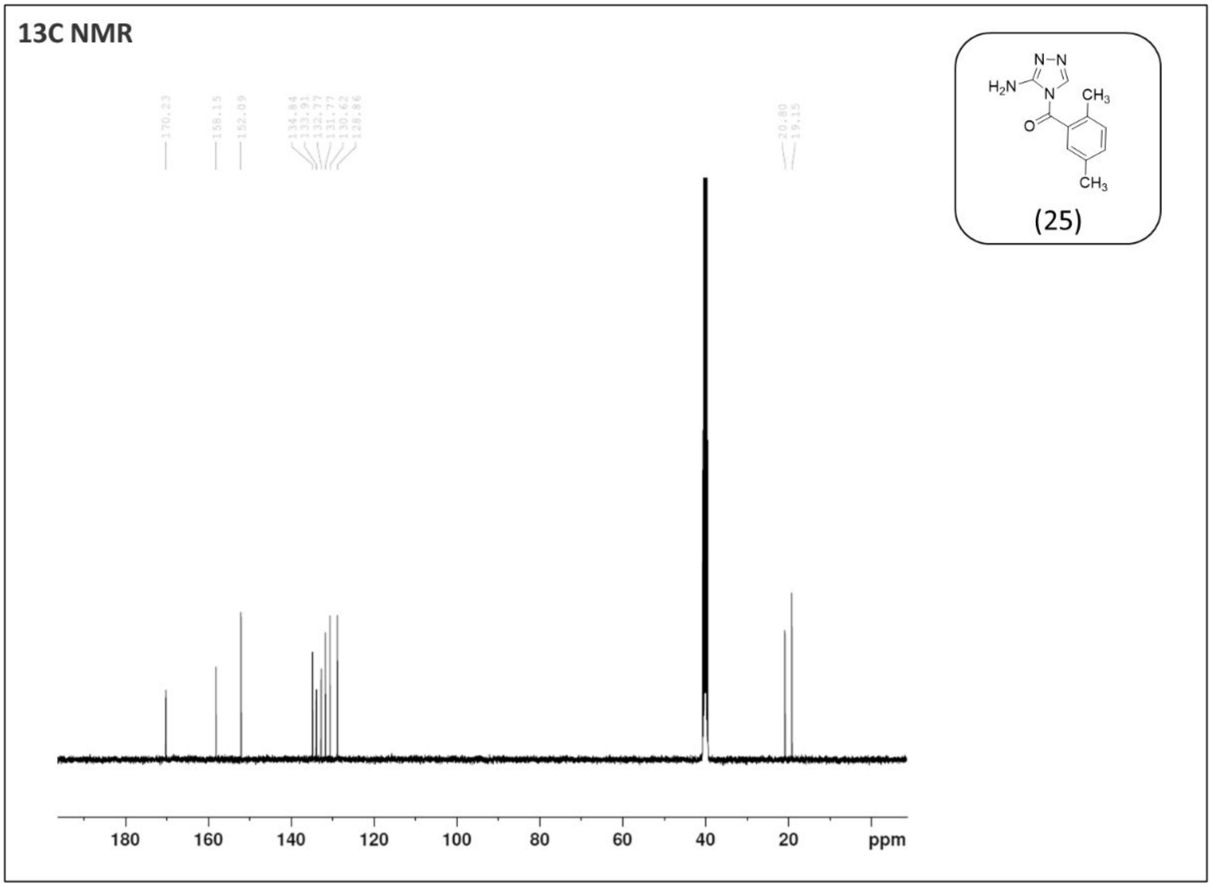

Figure S16. ^{13}C NMR of Molecule (25).

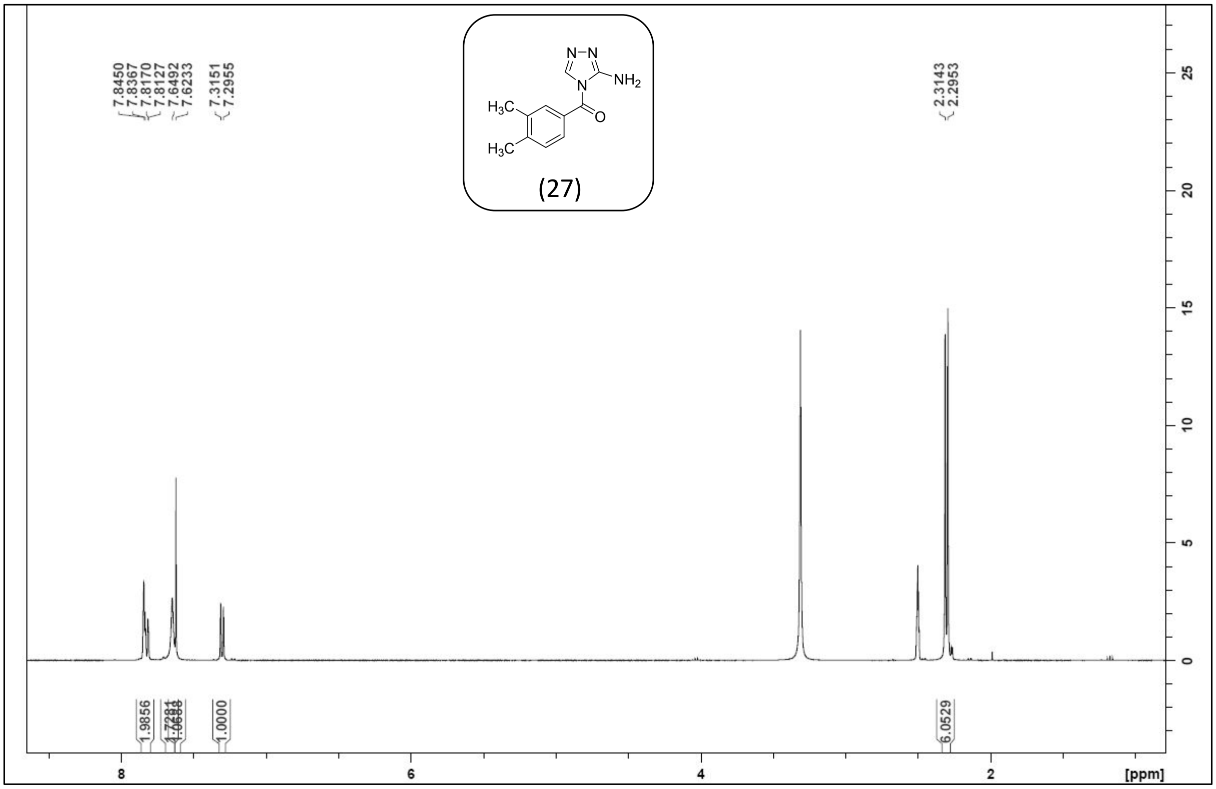

Figure S17. ^{1}H NMR of Molecule (27).

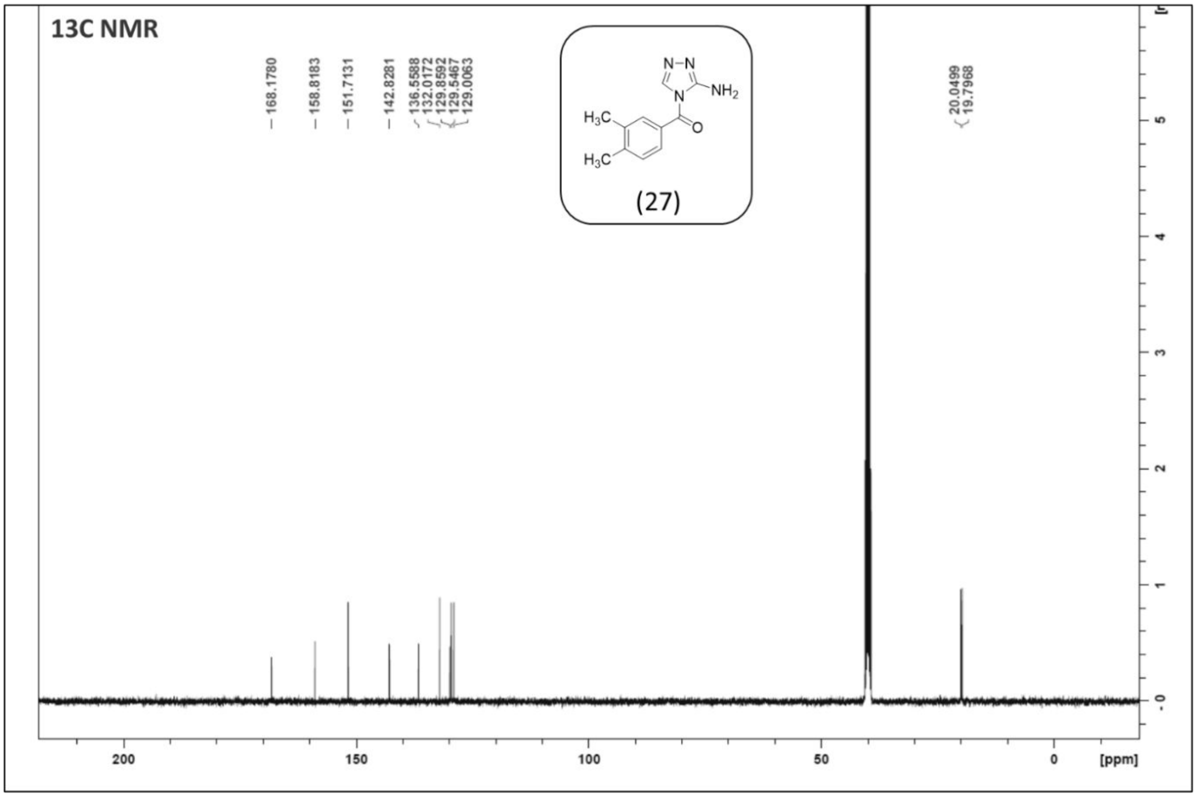

Figure S18. ^{13}C NMR of Molecule (27).
